# Supplementary figures and images for: N-terminus of Drosophila melanogaster MSL1 is critical for dosage compensation
Source: eLife. 2024 Dec 19;13:RP93241. doi: 10.7554/eLife.93241 (PMC11658772; doi:10.7554/eLife.93241)

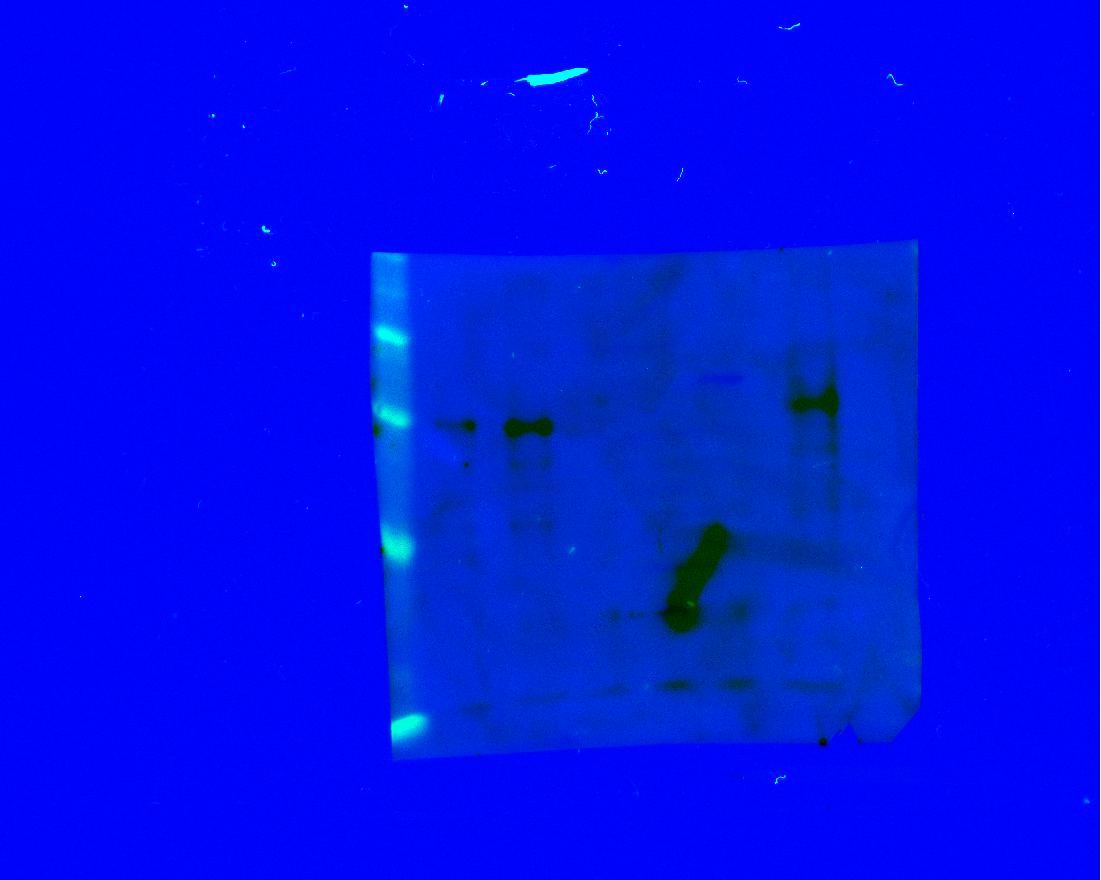

Supplement: Figure 1—source data 1. [file elife-93241-fig1-data1.zip › B _ msl2(Composite).tif]

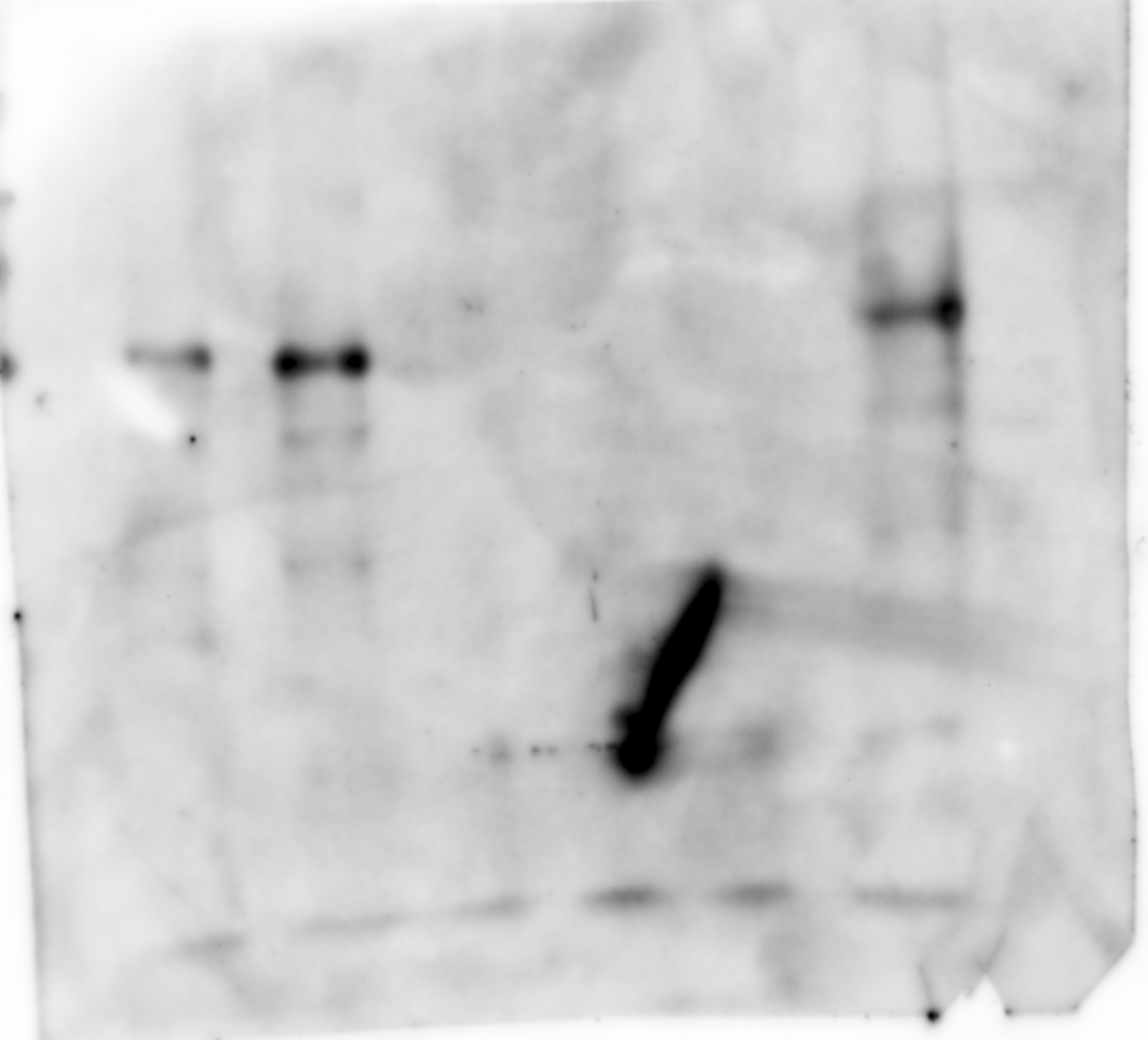

Supplement: Figure 1—source data 1. [file elife-93241-fig1-data1.zip › B _ msl2.tif]

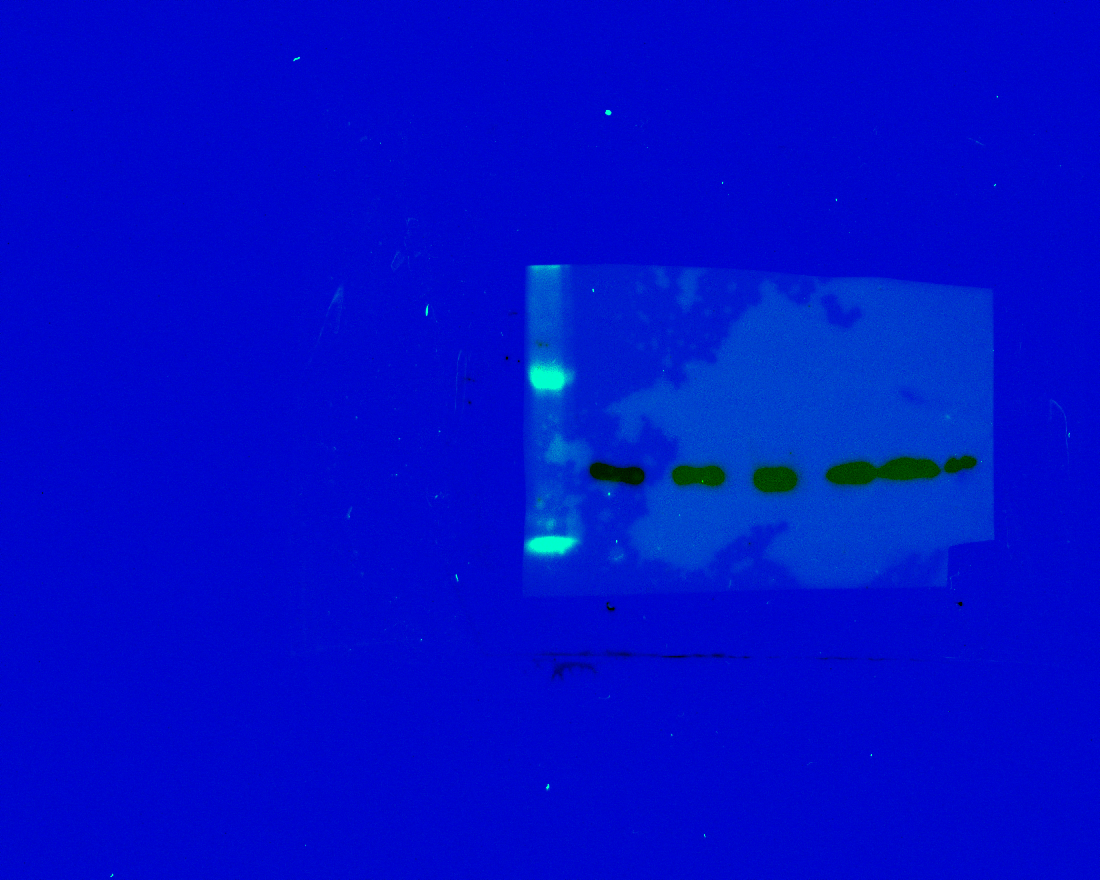

Supplement: Figure 1—source data 1. [file elife-93241-fig1-data1.zip › B_ lamin(Composite).tif]

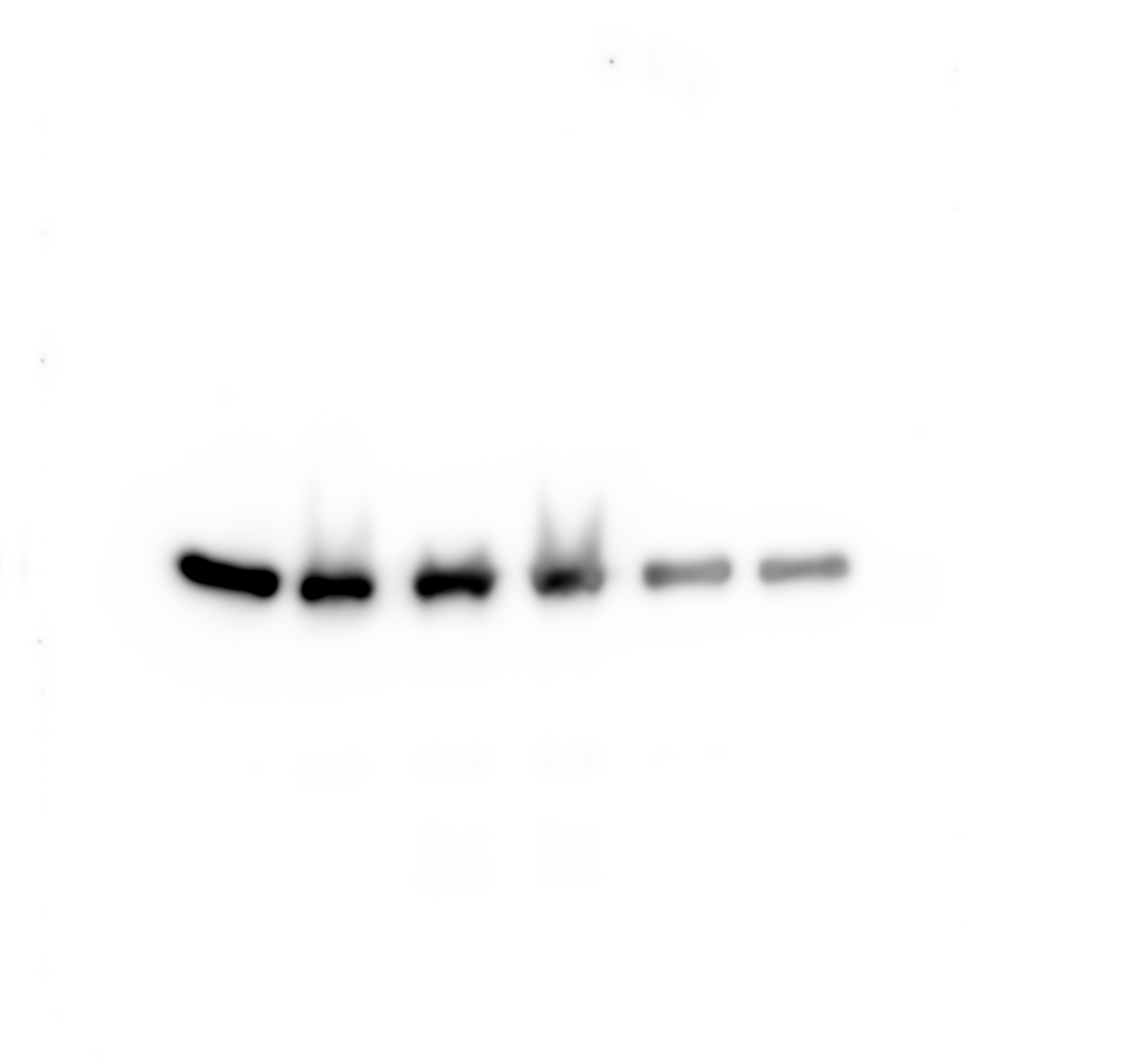

Supplement: Figure 1—source data 1. [file elife-93241-fig1-data1.zip › B_ lamin.tif]

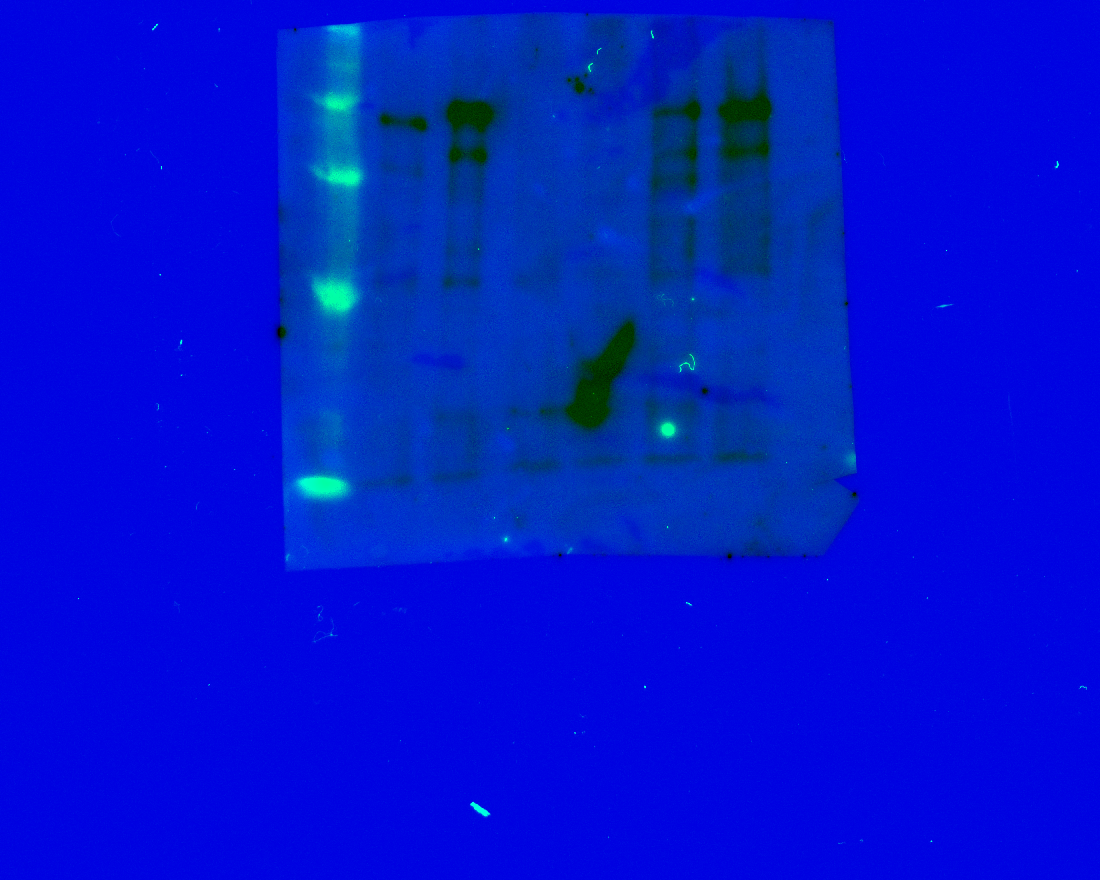

Supplement: Figure 1—source data 1. [file elife-93241-fig1-data1.zip › B_ msl1(Composite).tif]

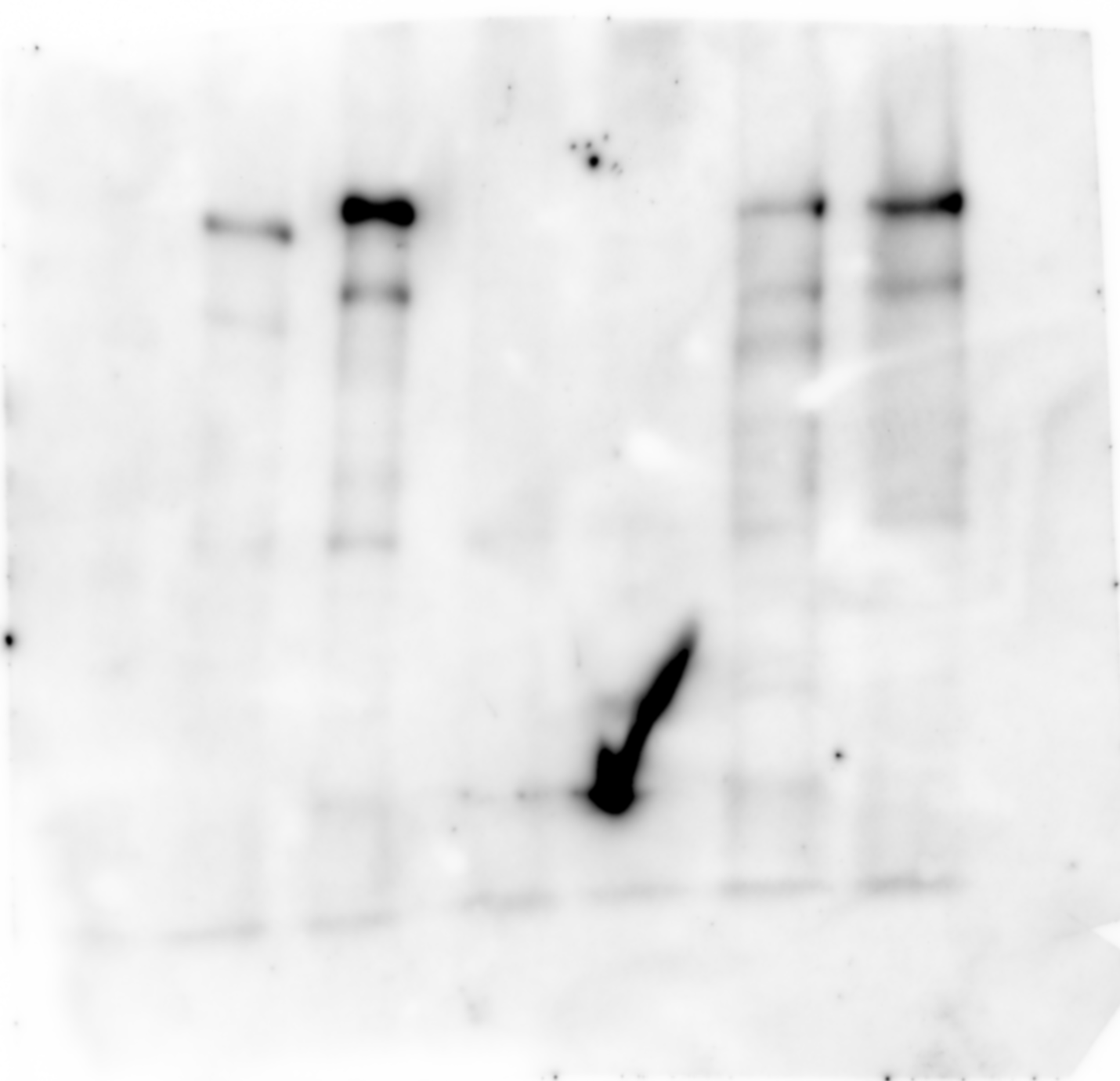

Supplement: Figure 1—source data 1. [file elife-93241-fig1-data1.zip › B_ msl1.tif]

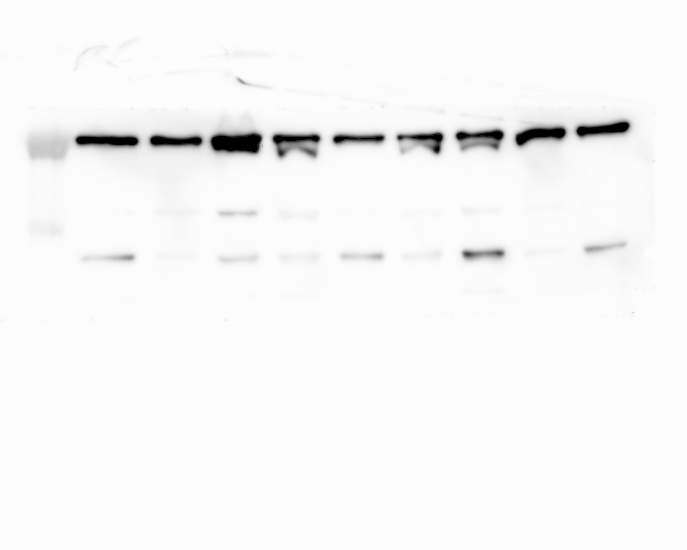

Supplement: Figure 1—source data 1. [file elife-93241-fig1-data1.zip › C_ lamin.tif]

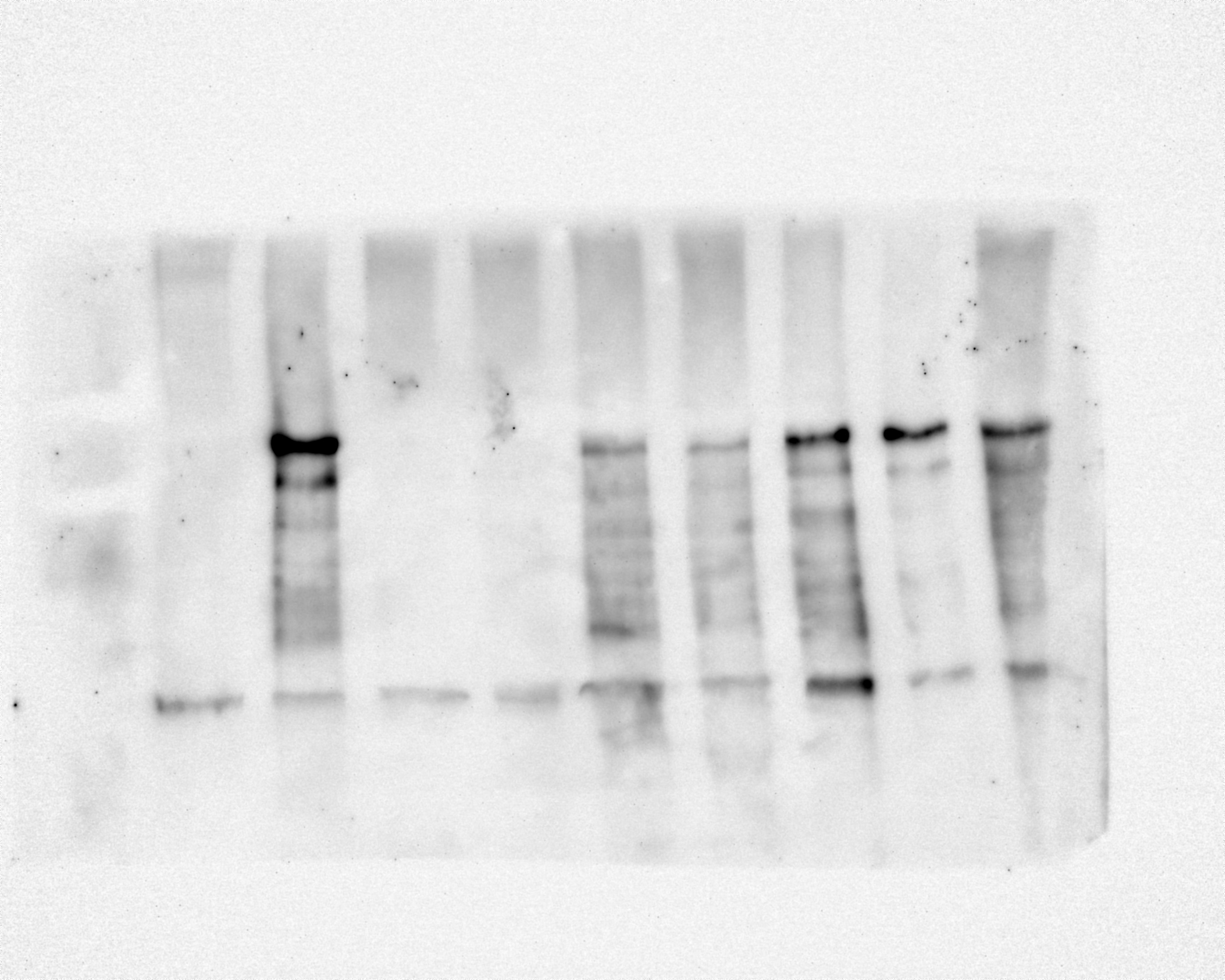

Supplement: Figure 1—source data 1. [file elife-93241-fig1-data1.zip › C_msl1.tif]

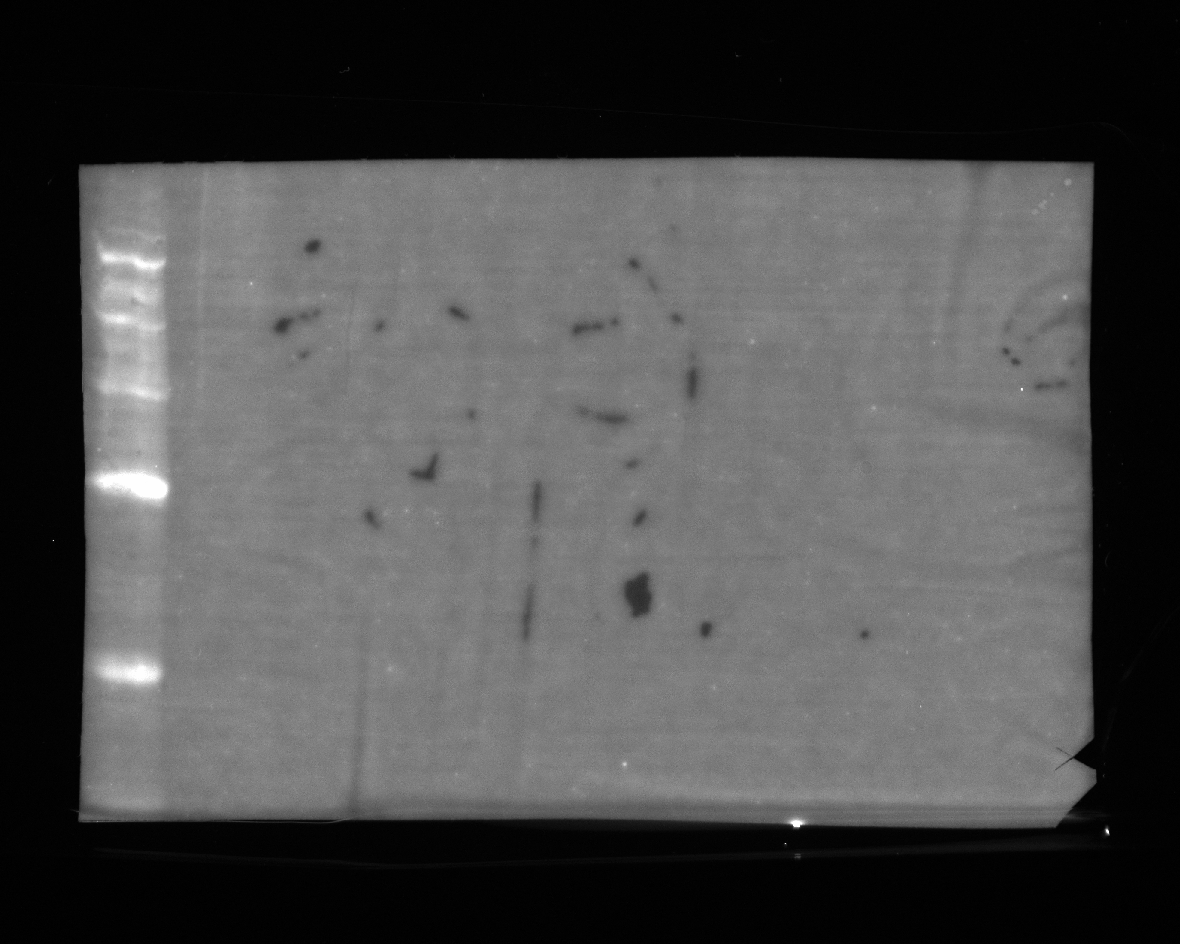

Supplement: Figure 1—source data 1. [file elife-93241-fig1-data1.zip › E_lamin(Alexa 647).tif]

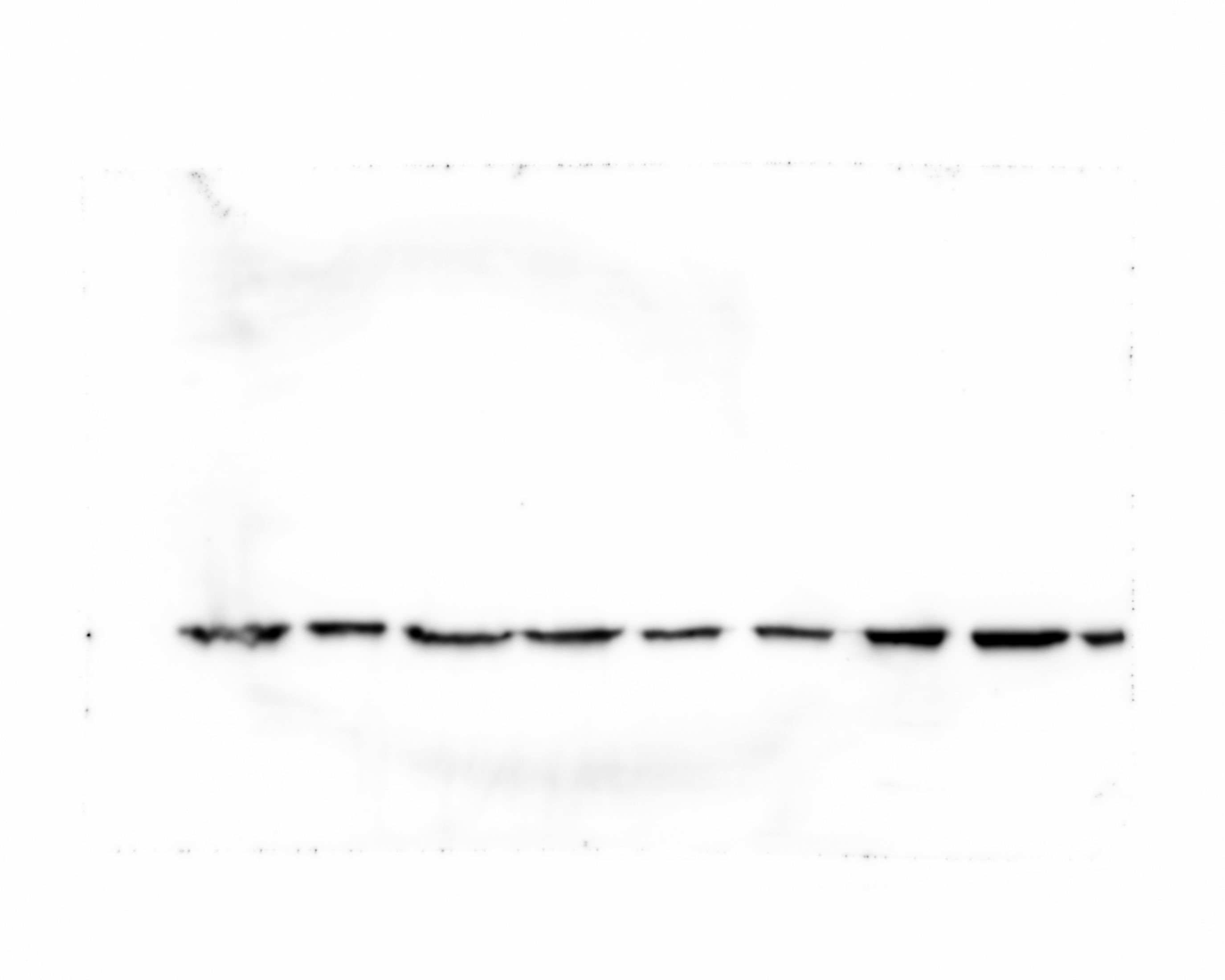

Supplement: Figure 1—source data 1. [file elife-93241-fig1-data1.zip › E_lamin.tif]

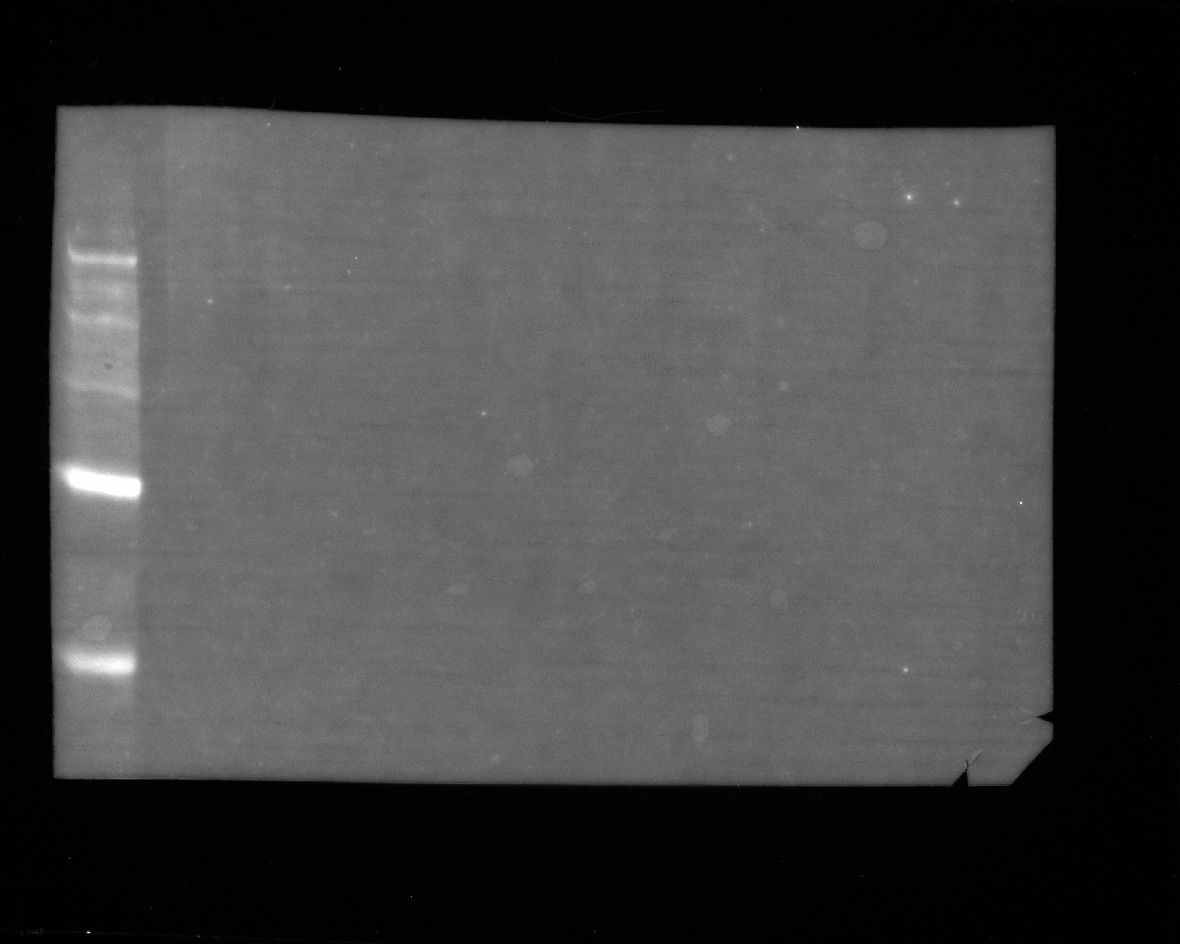

Supplement: Figure 1—source data 1. [file elife-93241-fig1-data1.zip › E_msl1(Alexa 647).tif]

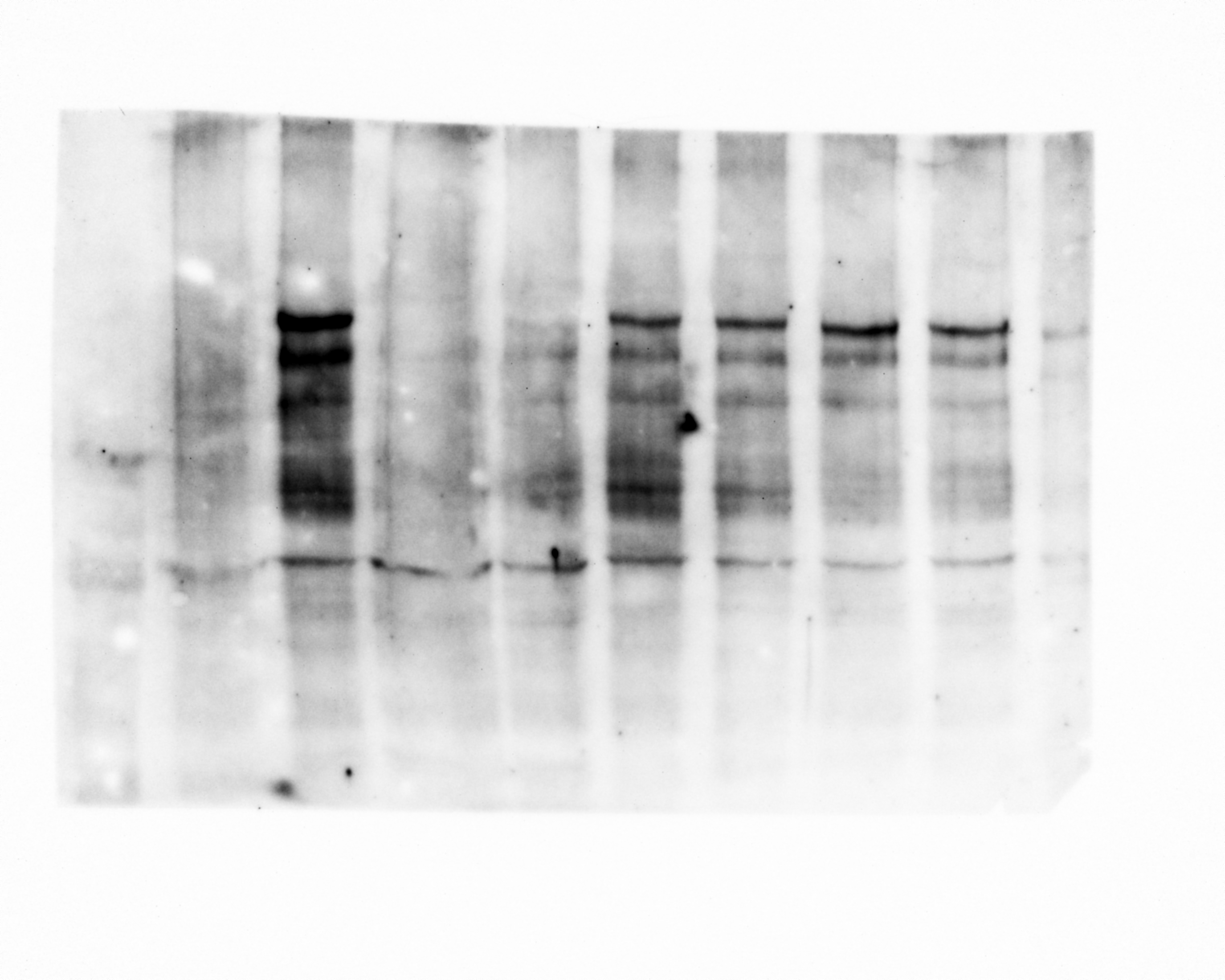

Supplement: Figure 1—source data 1. [file elife-93241-fig1-data1.zip › E_msl1.tif]

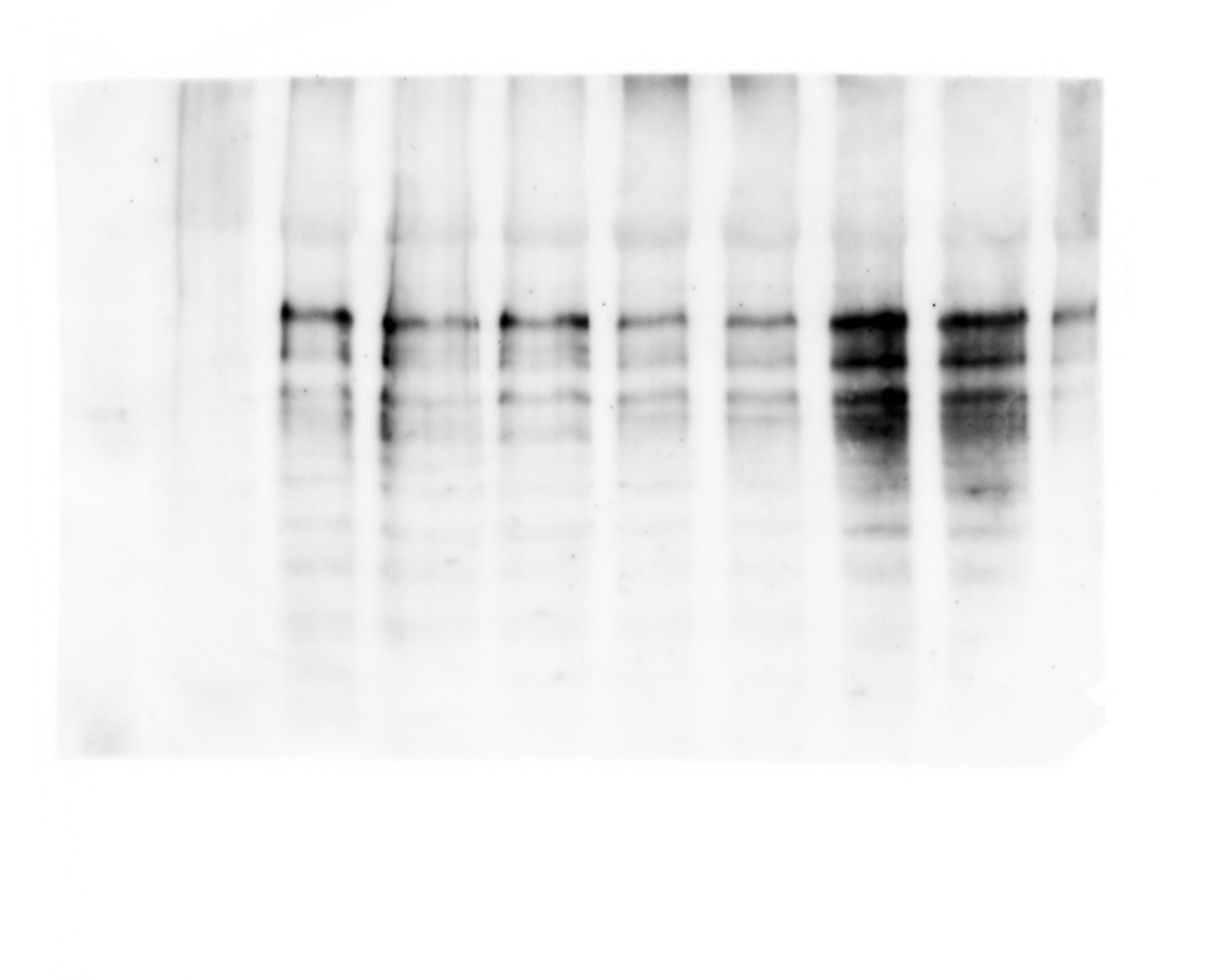

Supplement: Figure 1—source data 1. [file elife-93241-fig1-data1.zip › E_msl2.tif]

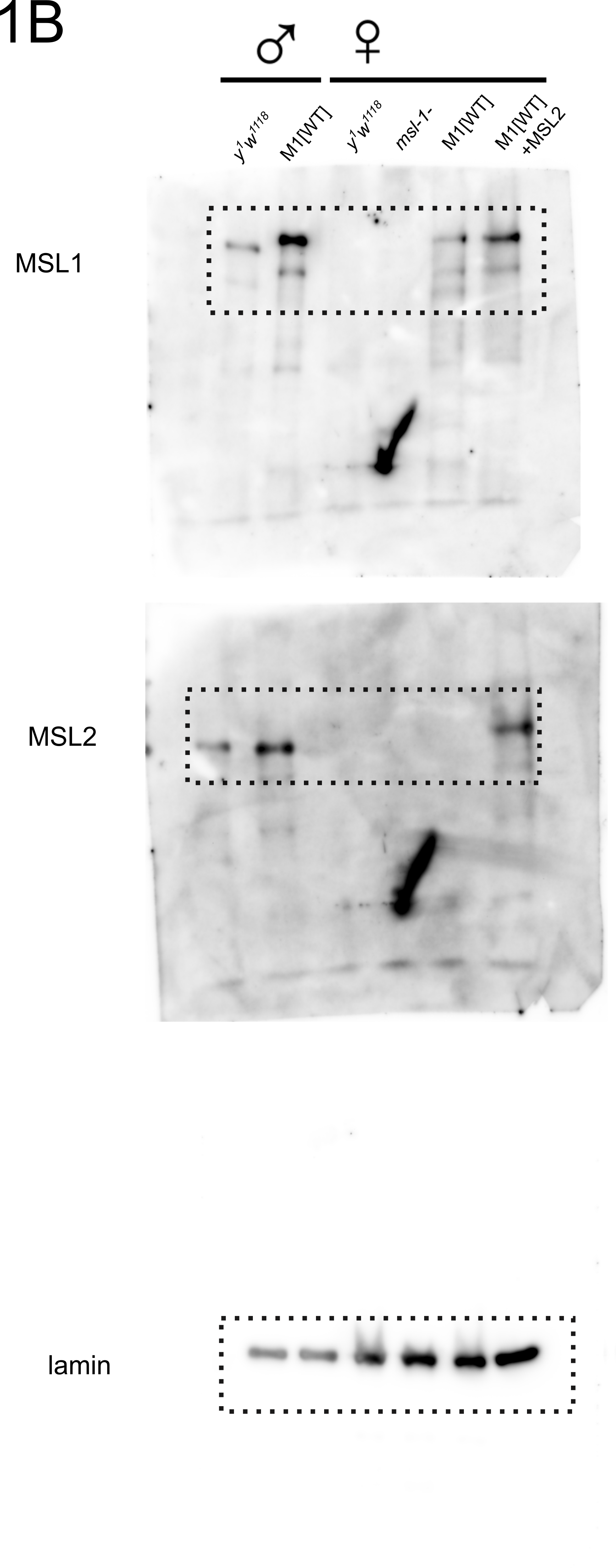

Supplement: Figure 1—source data 2. [file elife-93241-fig1-data2.zip › 1B.png]

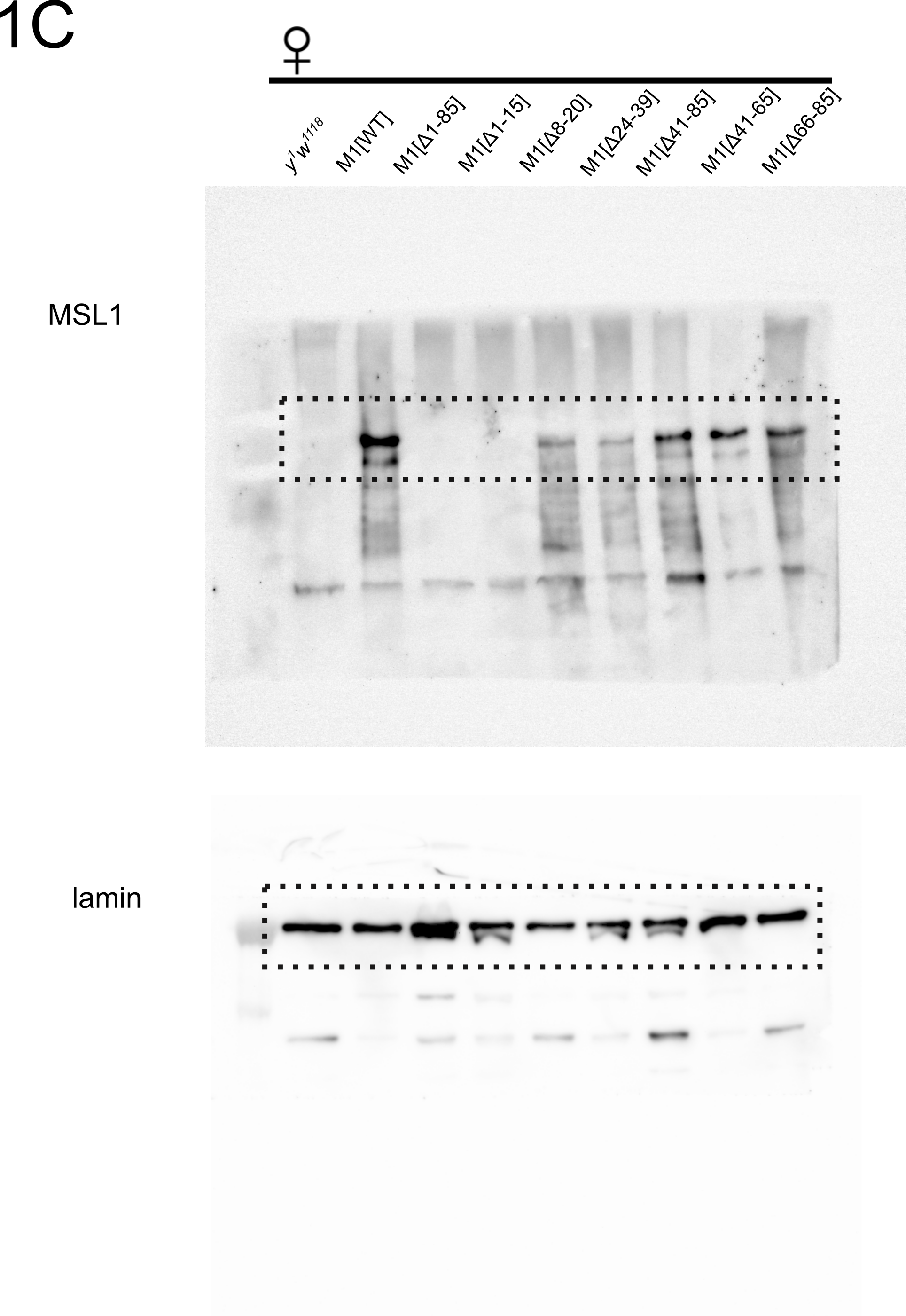

Supplement: Figure 1—source data 2. [file elife-93241-fig1-data2.zip › 1C.png]

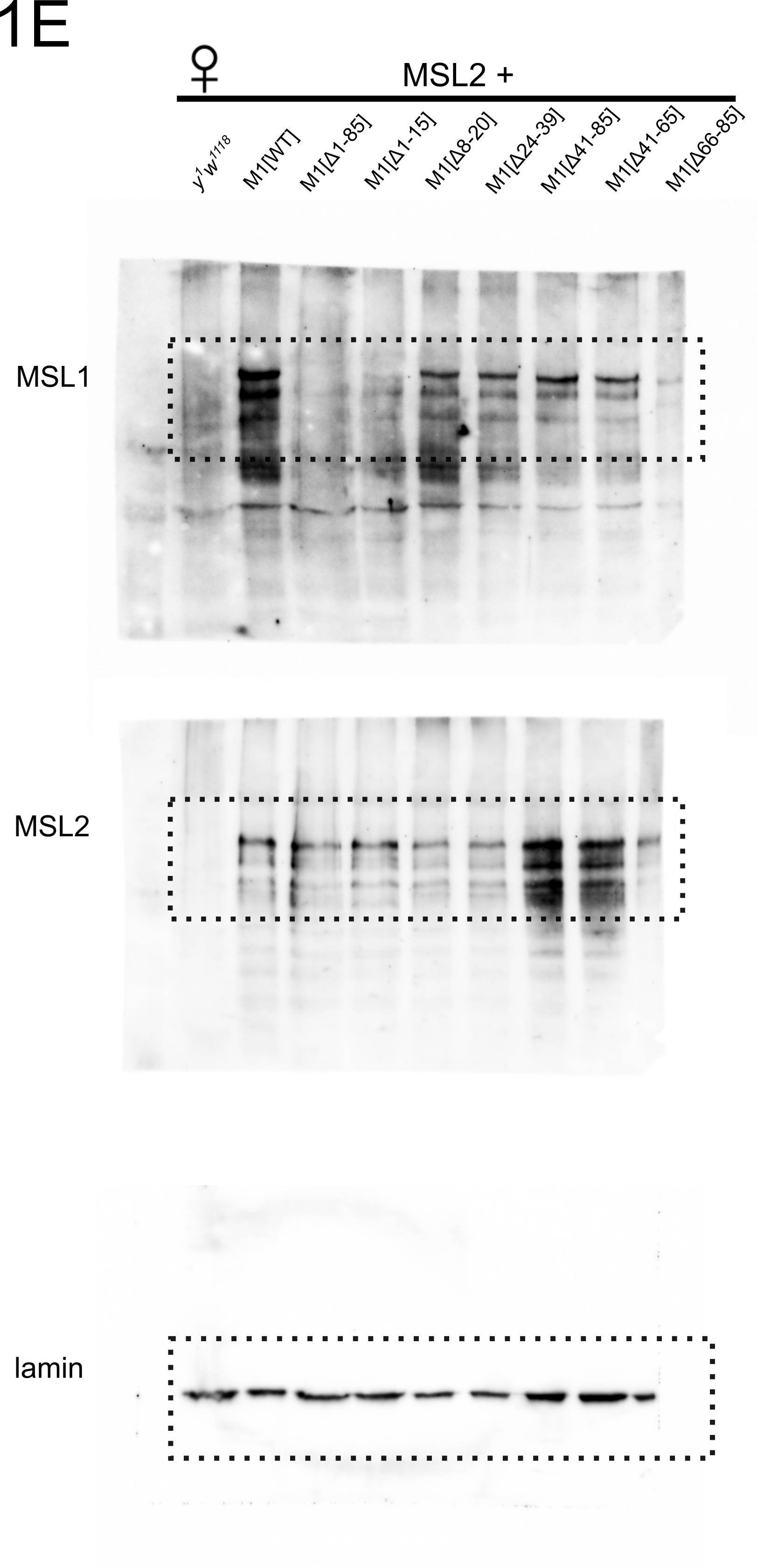

Supplement: Figure 1—source data 2. [file elife-93241-fig1-data2.zip › 1E.png]

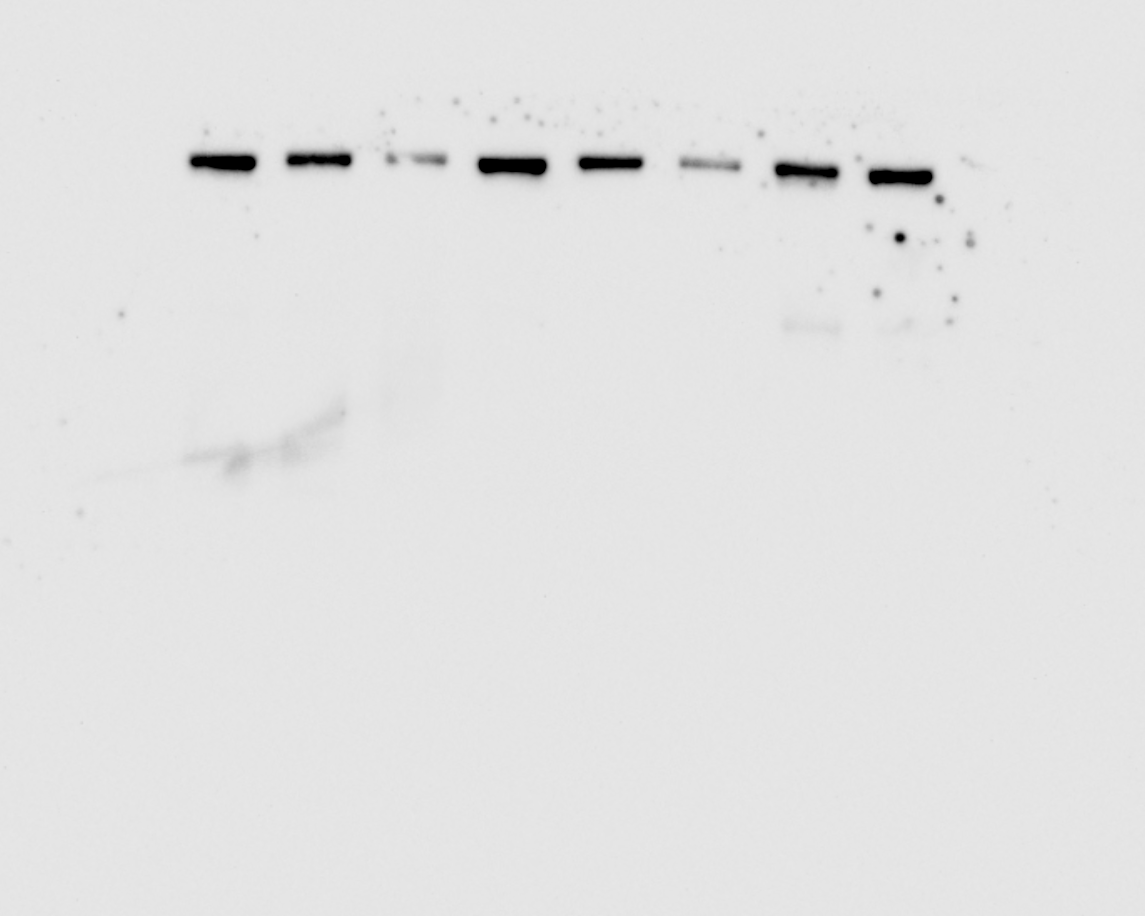

Supplement: Figure 1—figure supplement 3—source data 1. [file elife-93241-fig1-figsupp3-data1.zip › Figure 1 - supplement 3 - source data 1/lamin.tif]

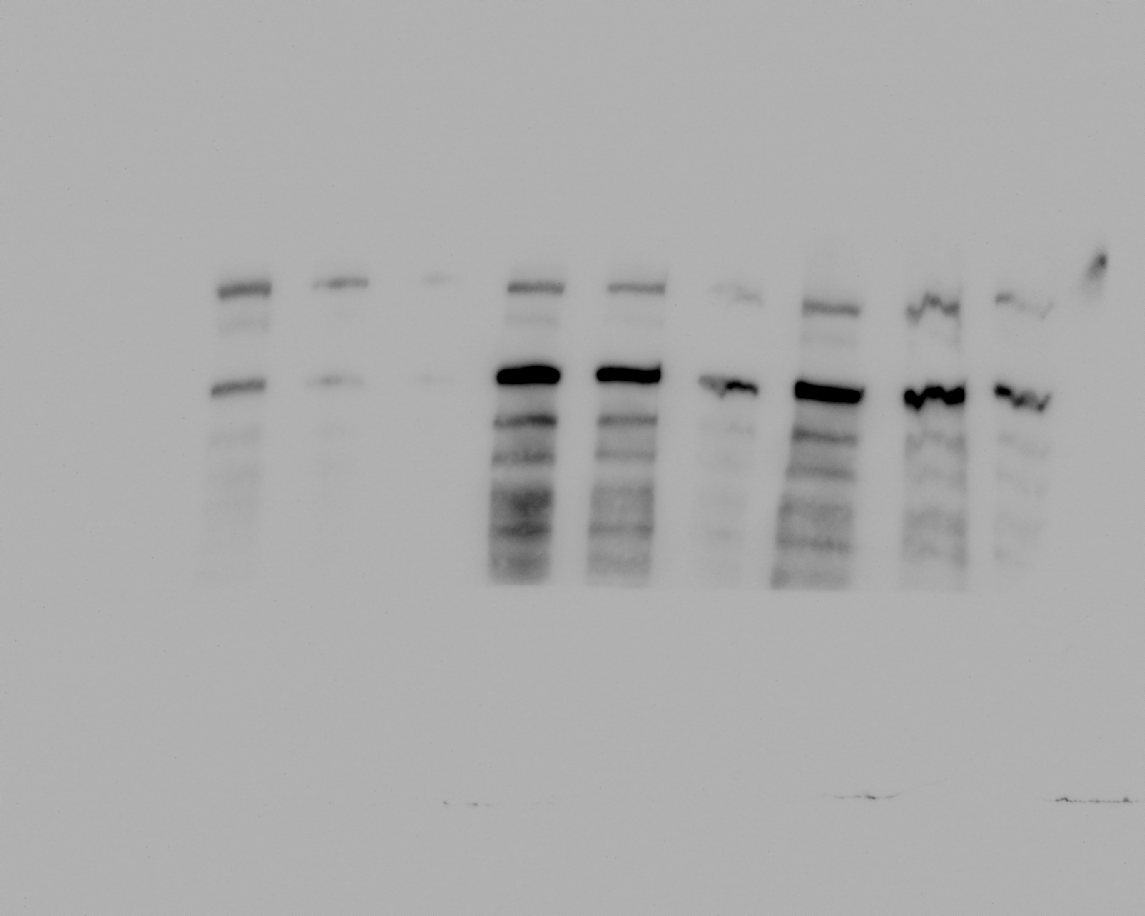

Supplement: Figure 1—figure supplement 3—source data 1. [file elife-93241-fig1-figsupp3-data1.zip › Figure 1 - supplement 3 - source data 1/msl1.tif]

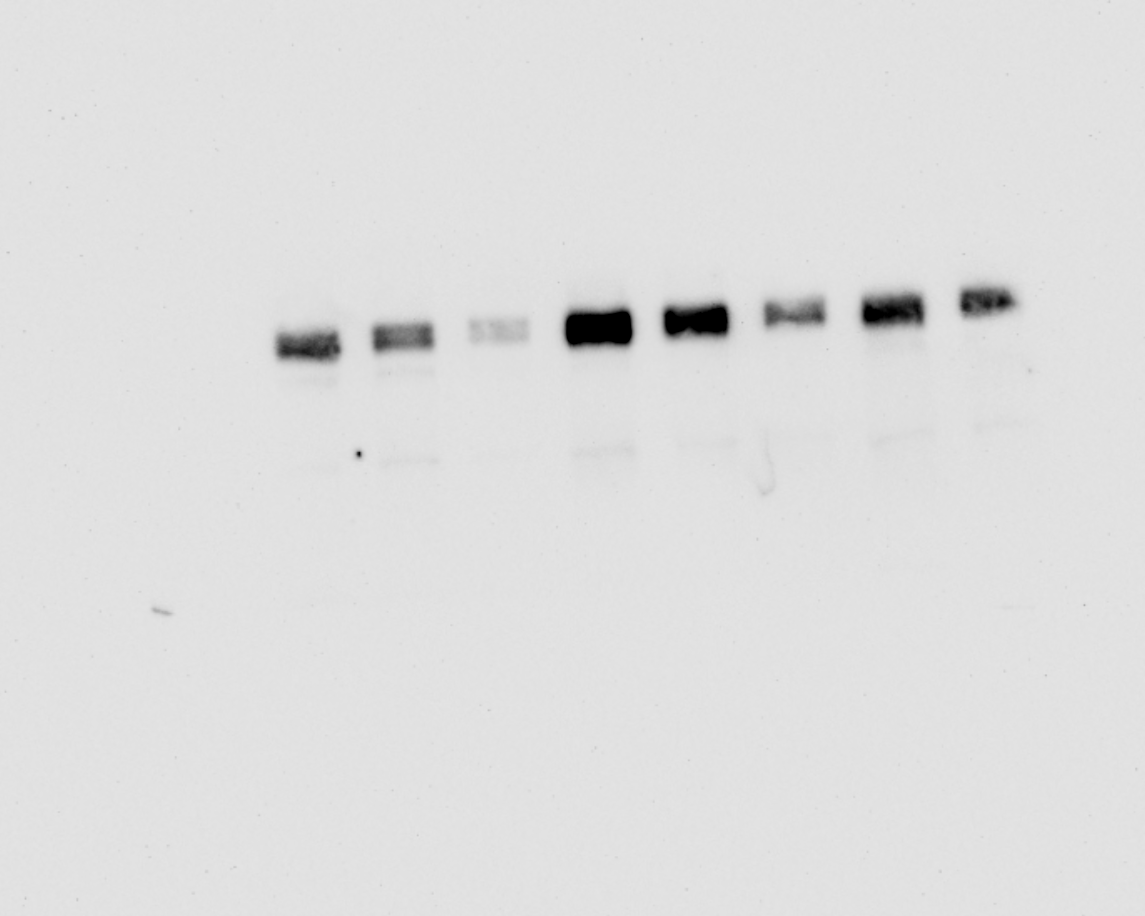

Supplement: Figure 1—figure supplement 3—source data 1. [file elife-93241-fig1-figsupp3-data1.zip › Figure 1 - supplement 3 - source data 1/msl2.tif]

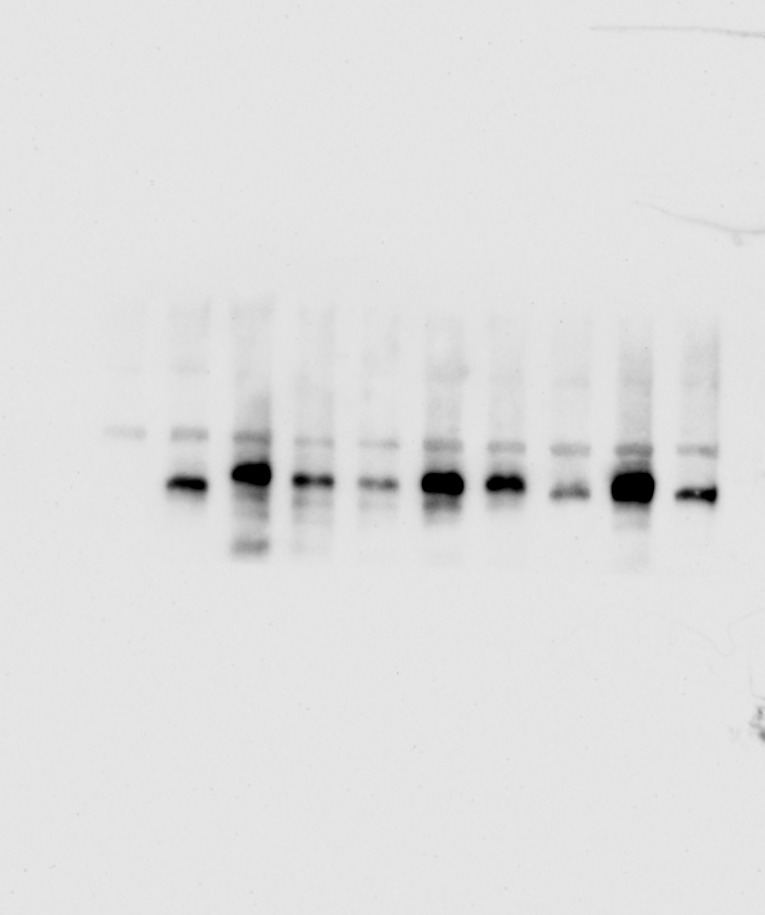

Supplement: Figure 1—figure supplement 3—source data 1. [file elife-93241-fig1-figsupp3-data1.zip › Figure 1 - supplement 3 - source data 1/msl2f.tif]

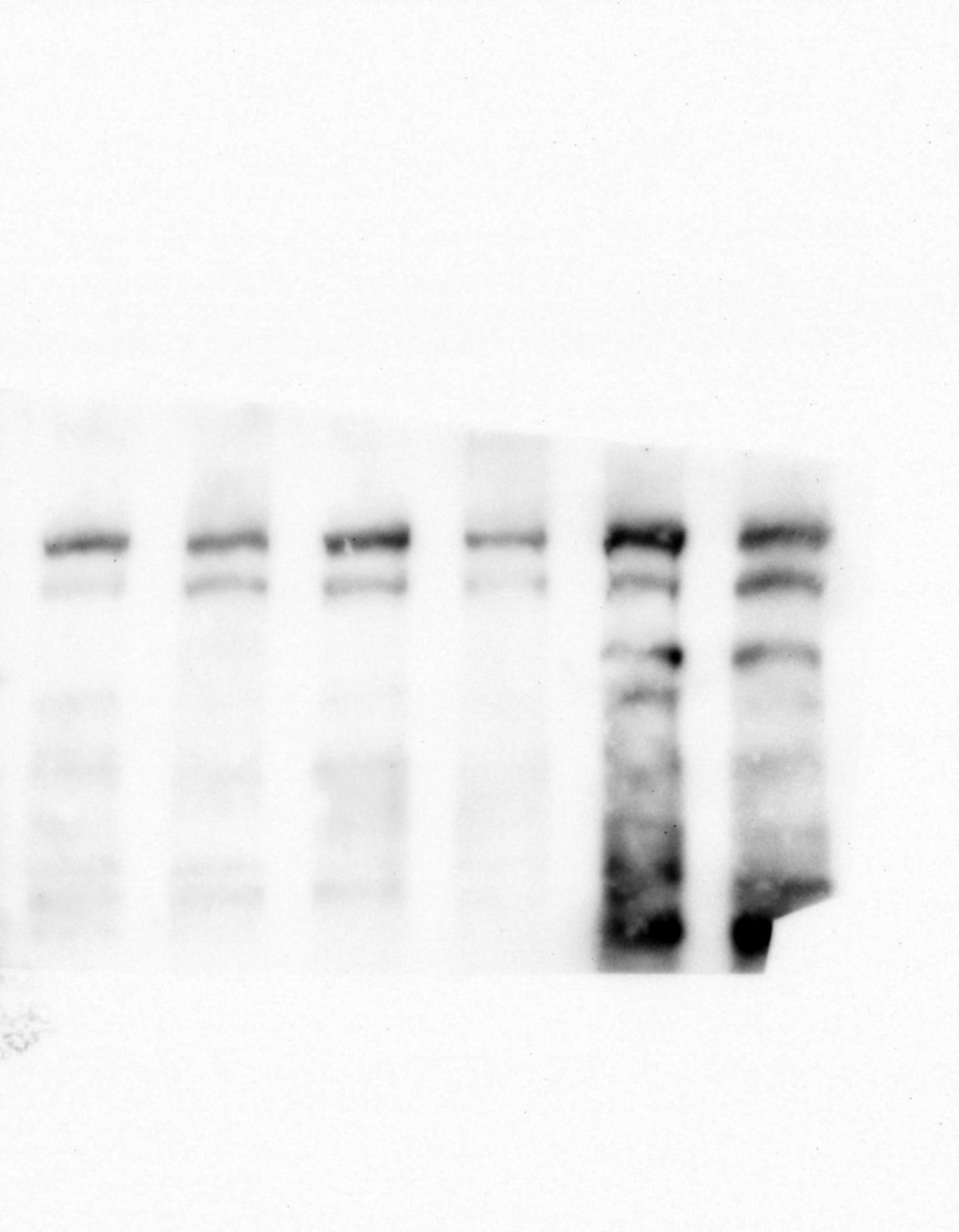

Supplement: Figure 1—figure supplement 3—source data 1. [file elife-93241-fig1-figsupp3-data1.zip › Figure 1 - supplement 3 - source data 1/msl1f.tif]

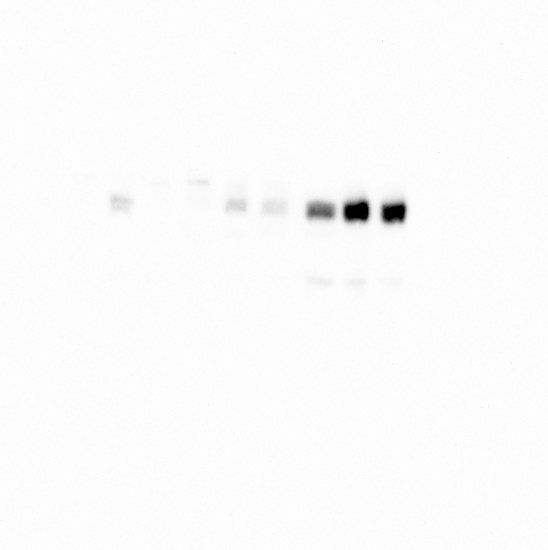

Supplement: Figure 1—figure supplement 3—source data 1. [file elife-93241-fig1-figsupp3-data1.zip › Figure 1 - supplement 3 - source data 1/msl3.tif]

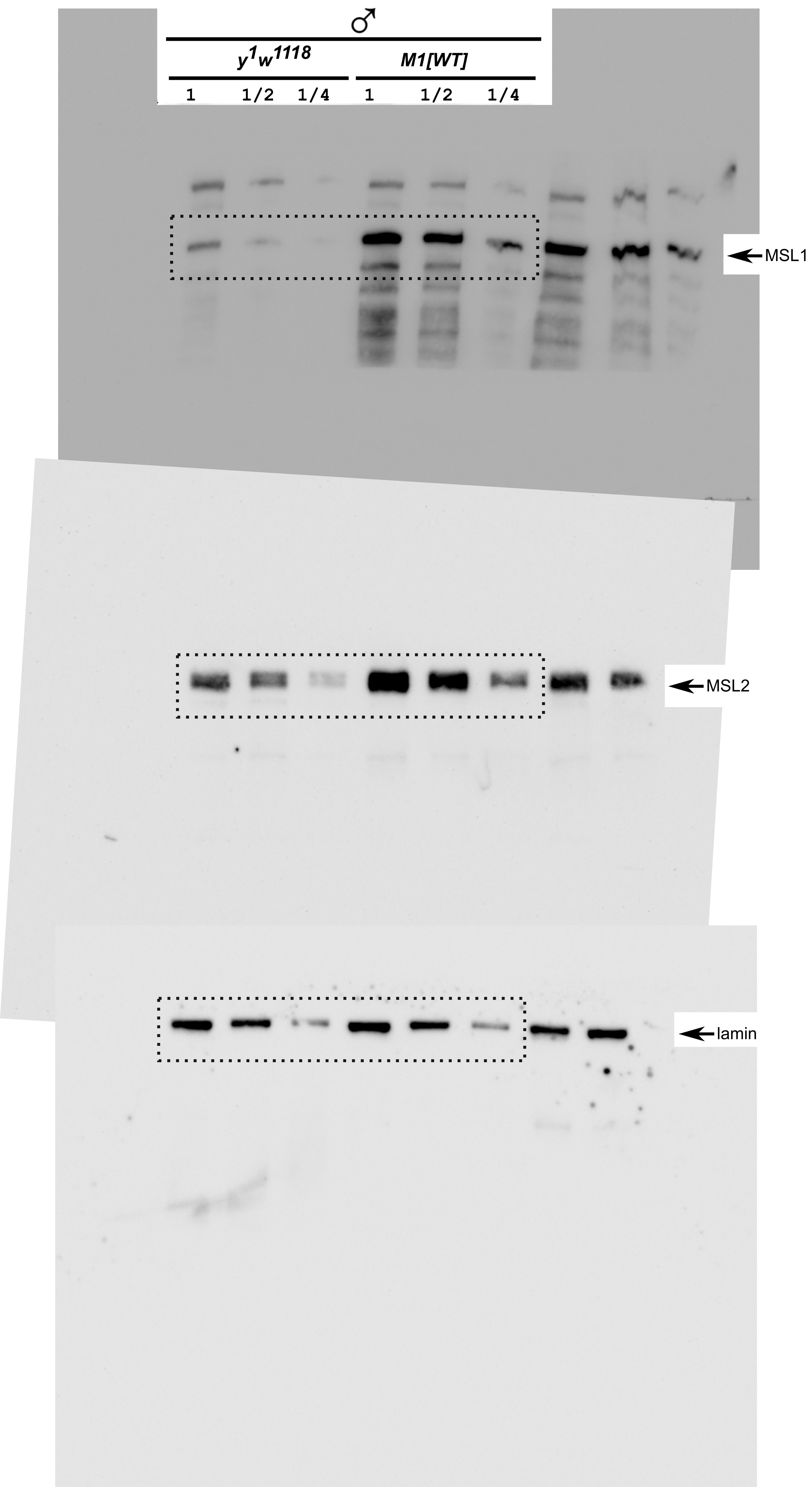

Supplement: Figure 1—figure supplement 3—source data 2. [file elife-93241-fig1-figsupp3-data2.zip › Figure 1 - supplement 3 - source data 2/Figure 1 - supplement 3A.png]

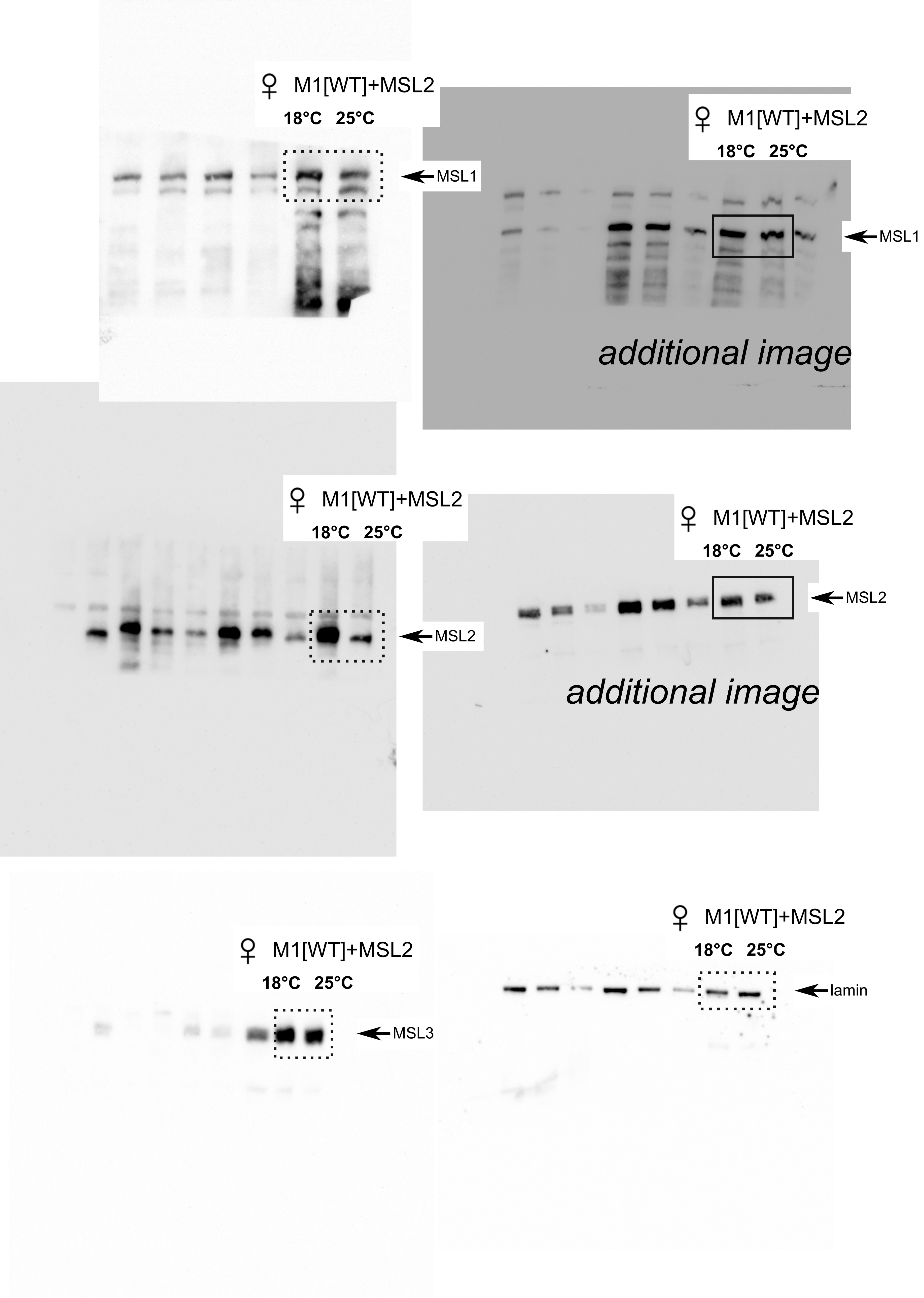

Supplement: Figure 1—figure supplement 3—source data 2. [file elife-93241-fig1-figsupp3-data2.zip › Figure 1 - supplement 3 - source data 2/Figure 1 - supplement 3B.png]

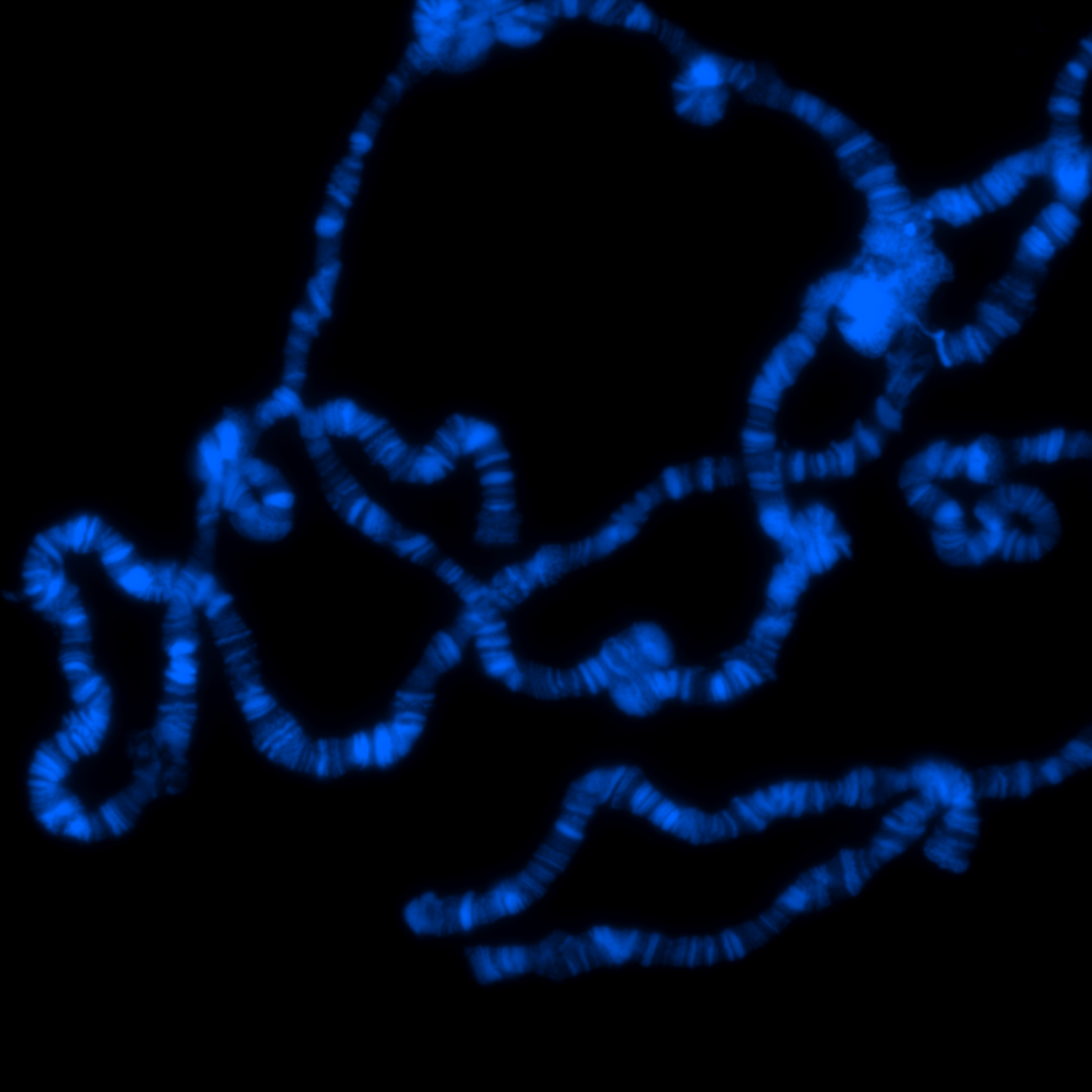

Supplement: Figure 2—source data 1. [file elife-93241-fig2-data1.zip › d(24-39)/2021-02-15_d24-39_6-2 D.tif]

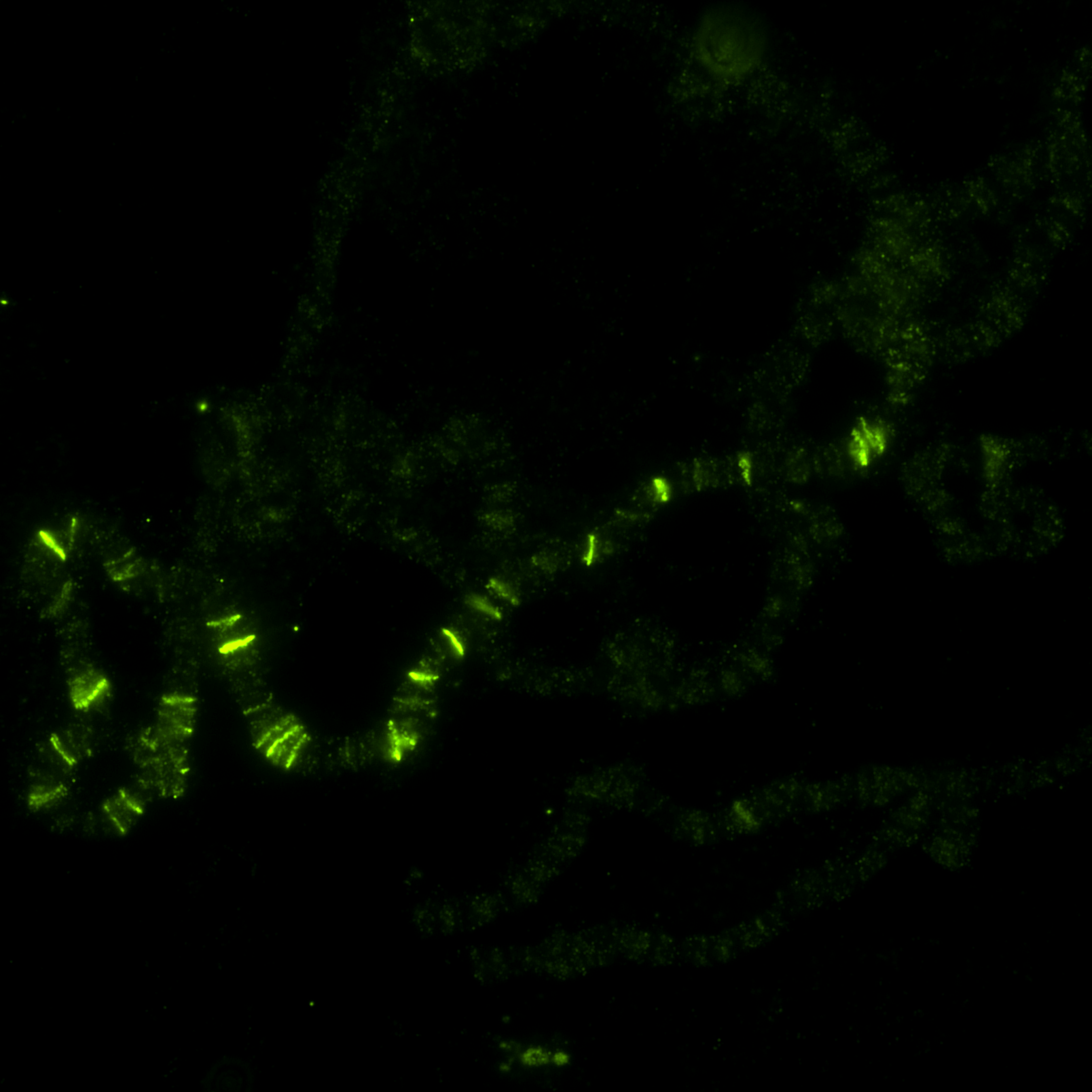

Supplement: Figure 2—source data 1. [file elife-93241-fig2-data1.zip › d(24-39)/2021-02-15_d24-39_6-2 F.tif]

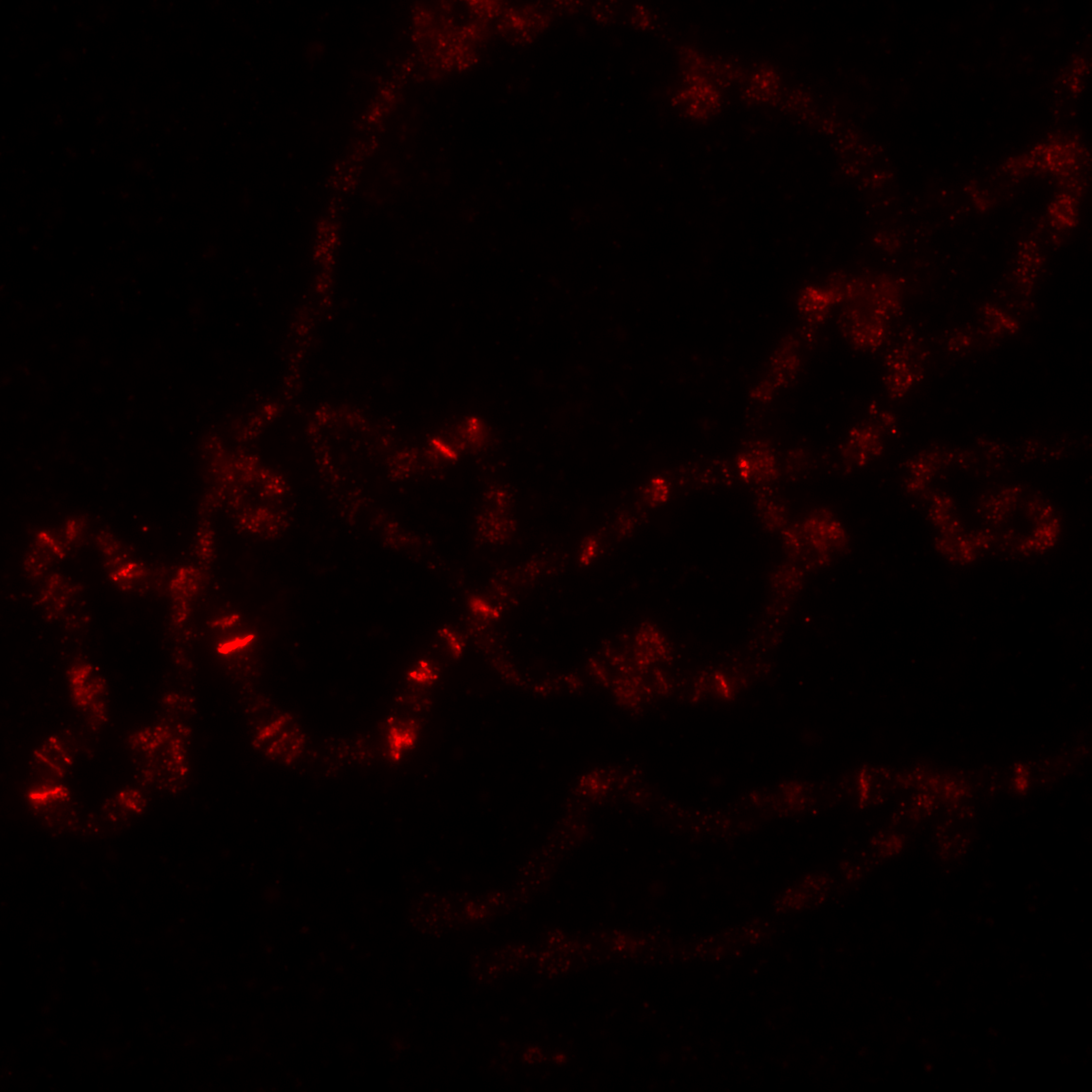

Supplement: Figure 2—source data 1. [file elife-93241-fig2-data1.zip › d(24-39)/2021-02-15_d24-39_6-2 T.tif]

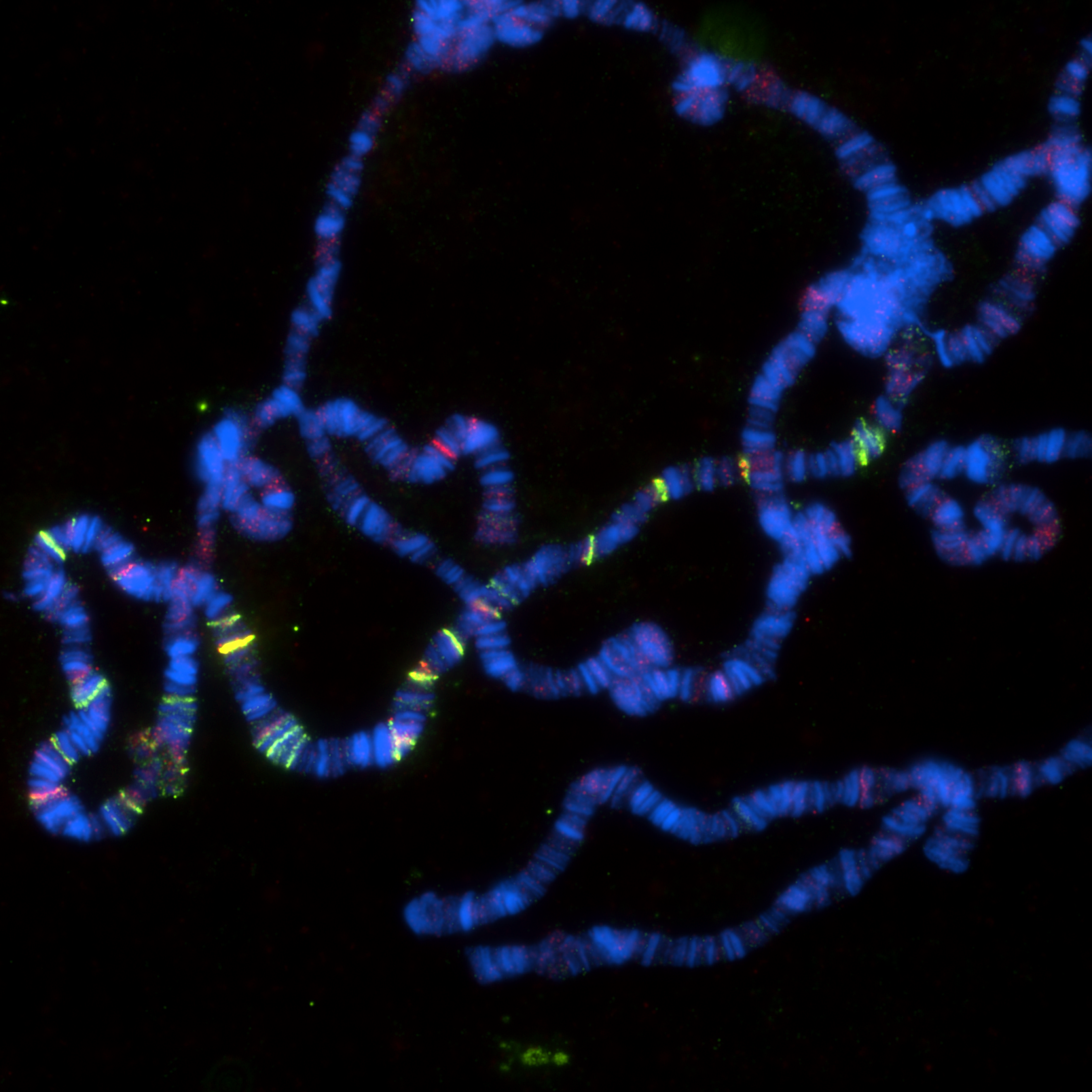

Supplement: Figure 2—source data 1. [file elife-93241-fig2-data1.zip › d(24-39)/Composite DFT.tif]

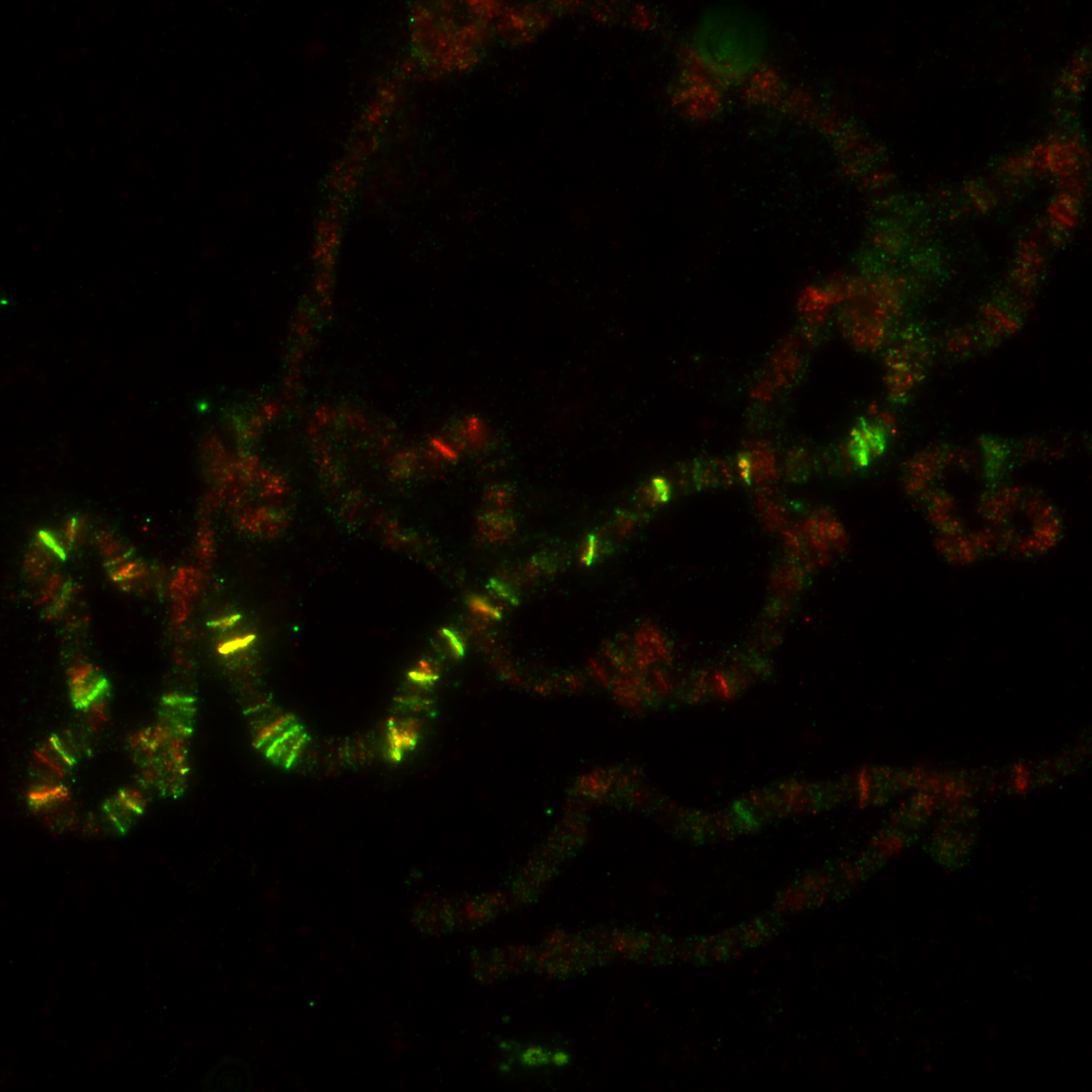

Supplement: Figure 2—source data 1. [file elife-93241-fig2-data1.zip › d(24-39)/Composite FT.tif]

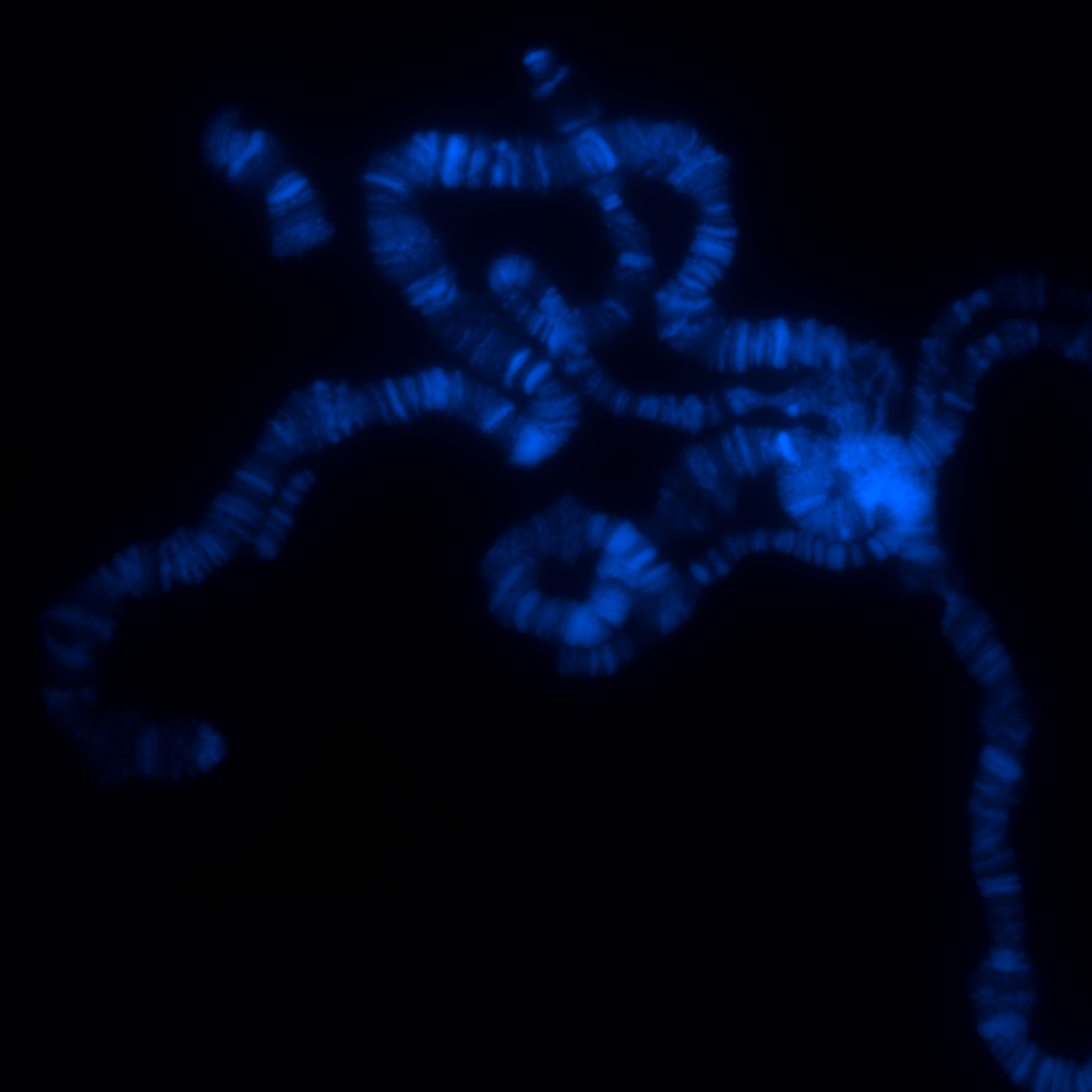

Supplement: Figure 2—source data 1. [file elife-93241-fig2-data1.zip › d(41-65)/DAPI.tif]

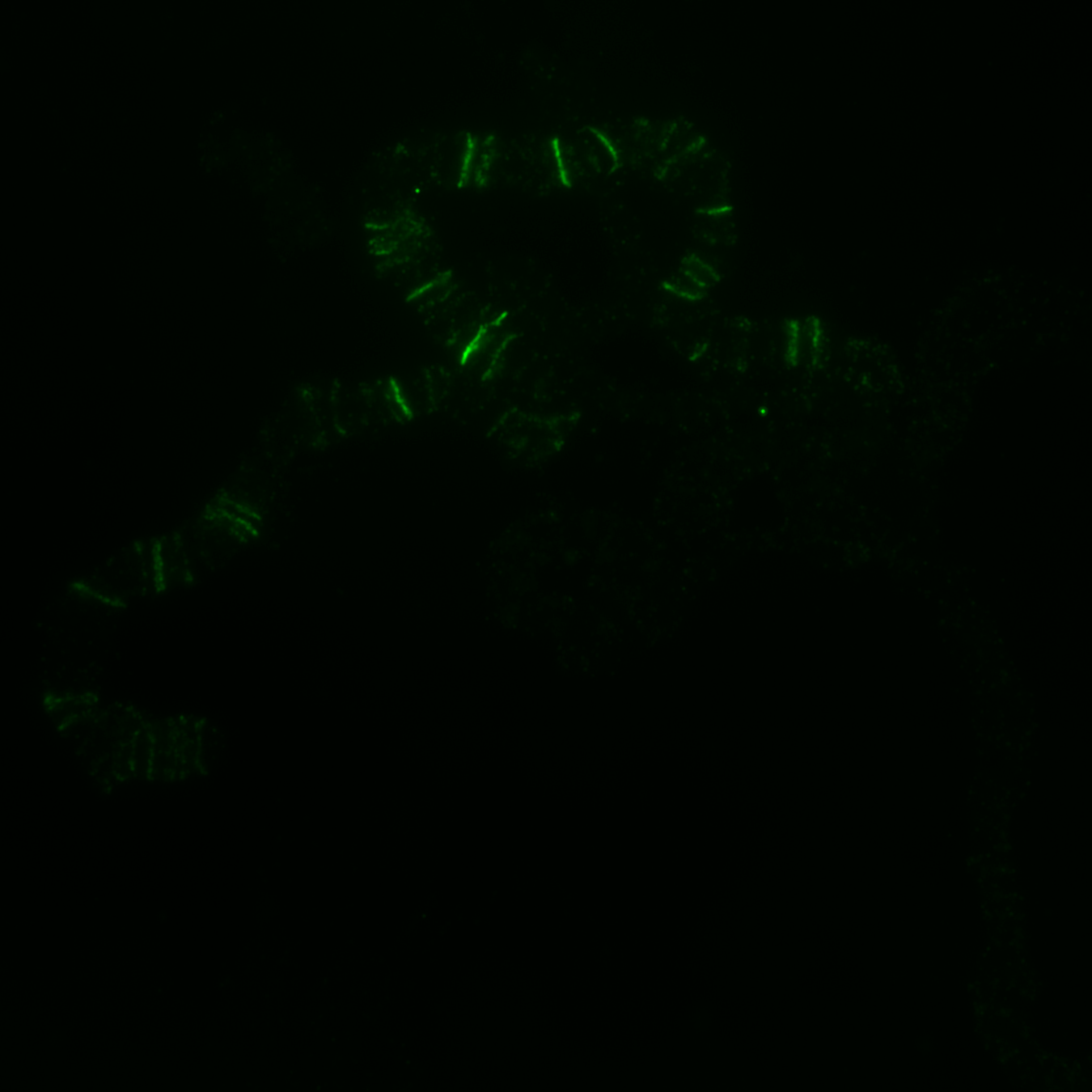

Supplement: Figure 2—source data 1. [file elife-93241-fig2-data1.zip › d(41-65)/MSL1.tif]

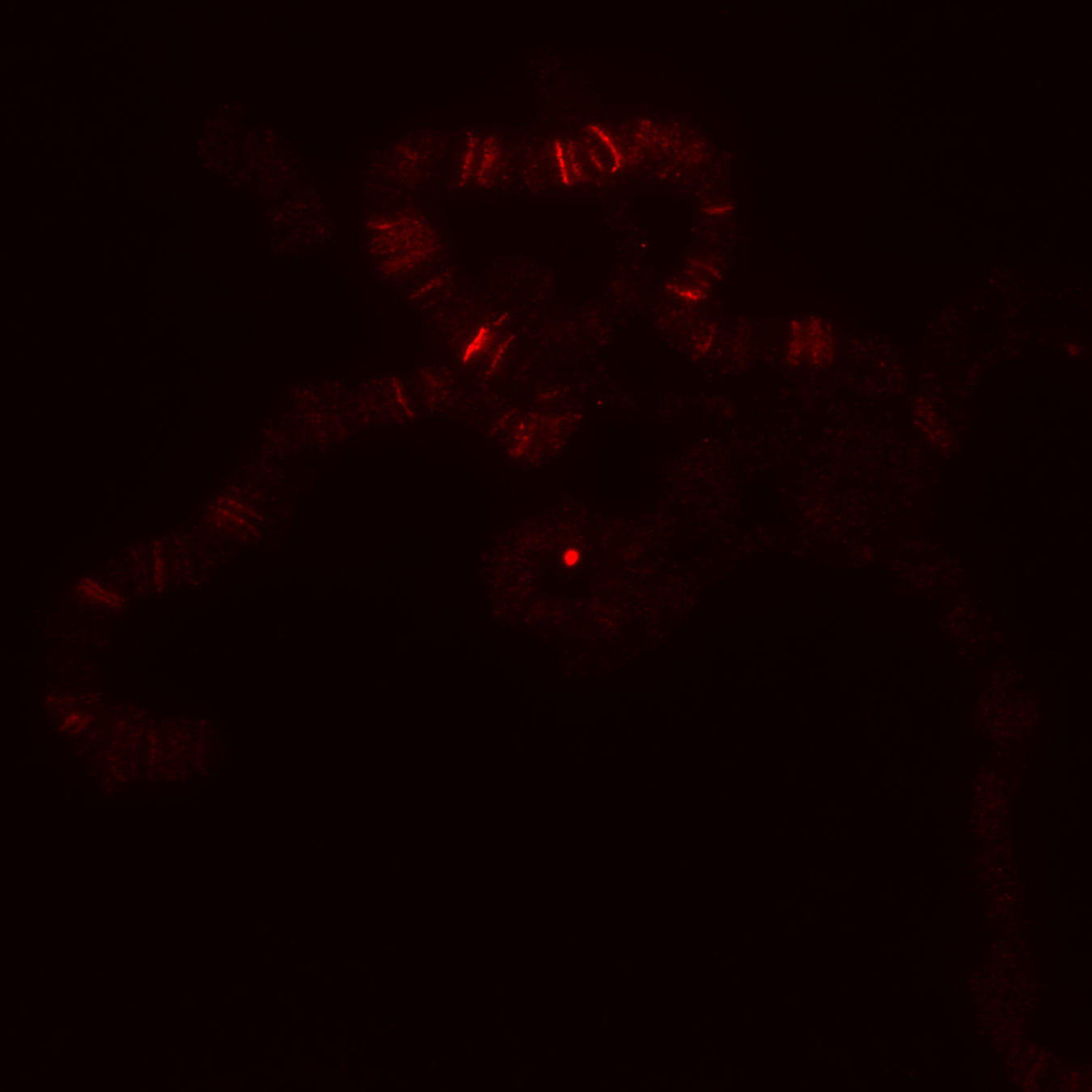

Supplement: Figure 2—source data 1. [file elife-93241-fig2-data1.zip › d(41-65)/MSL2.tif]

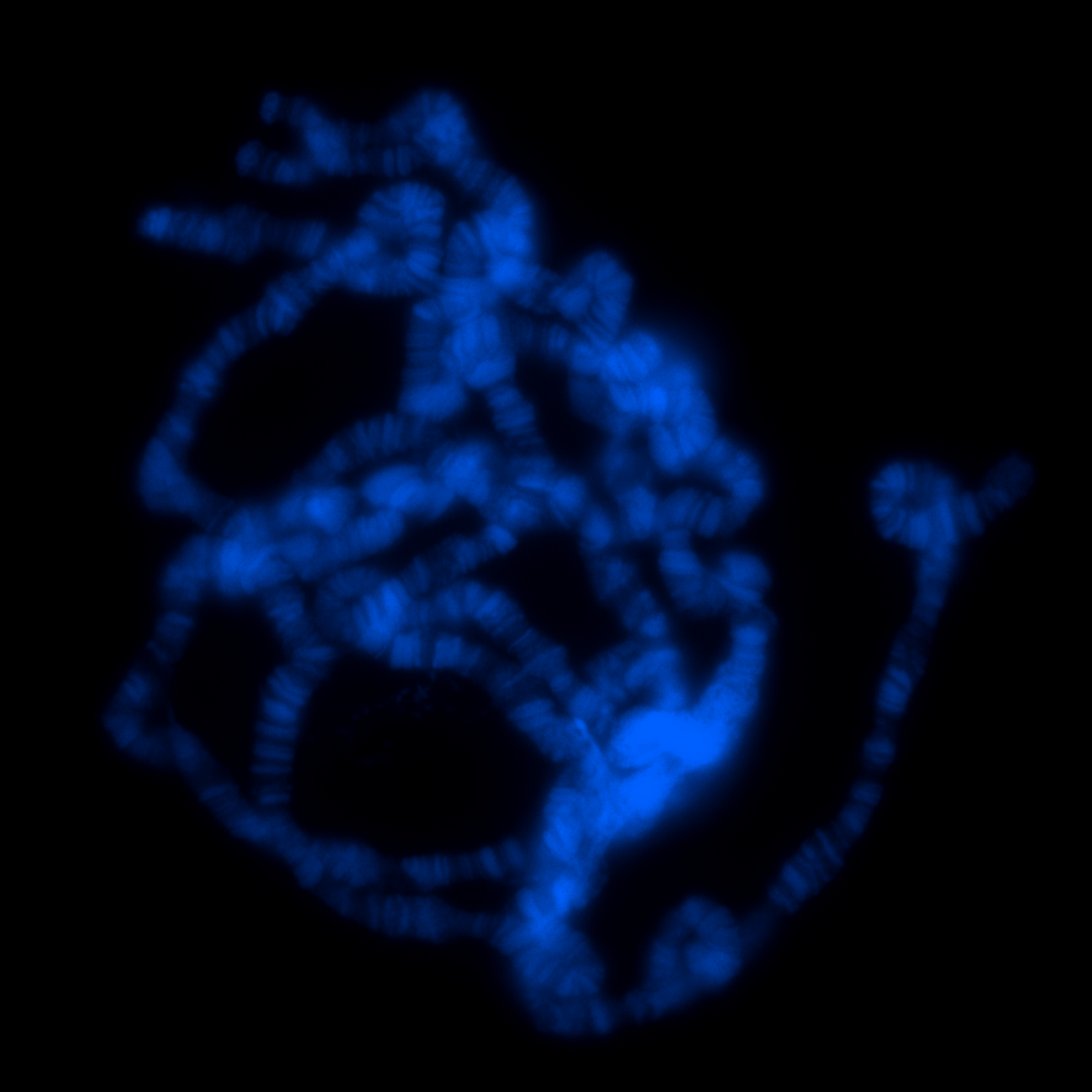

Supplement: Figure 2—source data 1. [file elife-93241-fig2-data1.zip › d(41-85)/20200310-msl1d41_85-6-1-D2.tif (RGB) (1).tif]

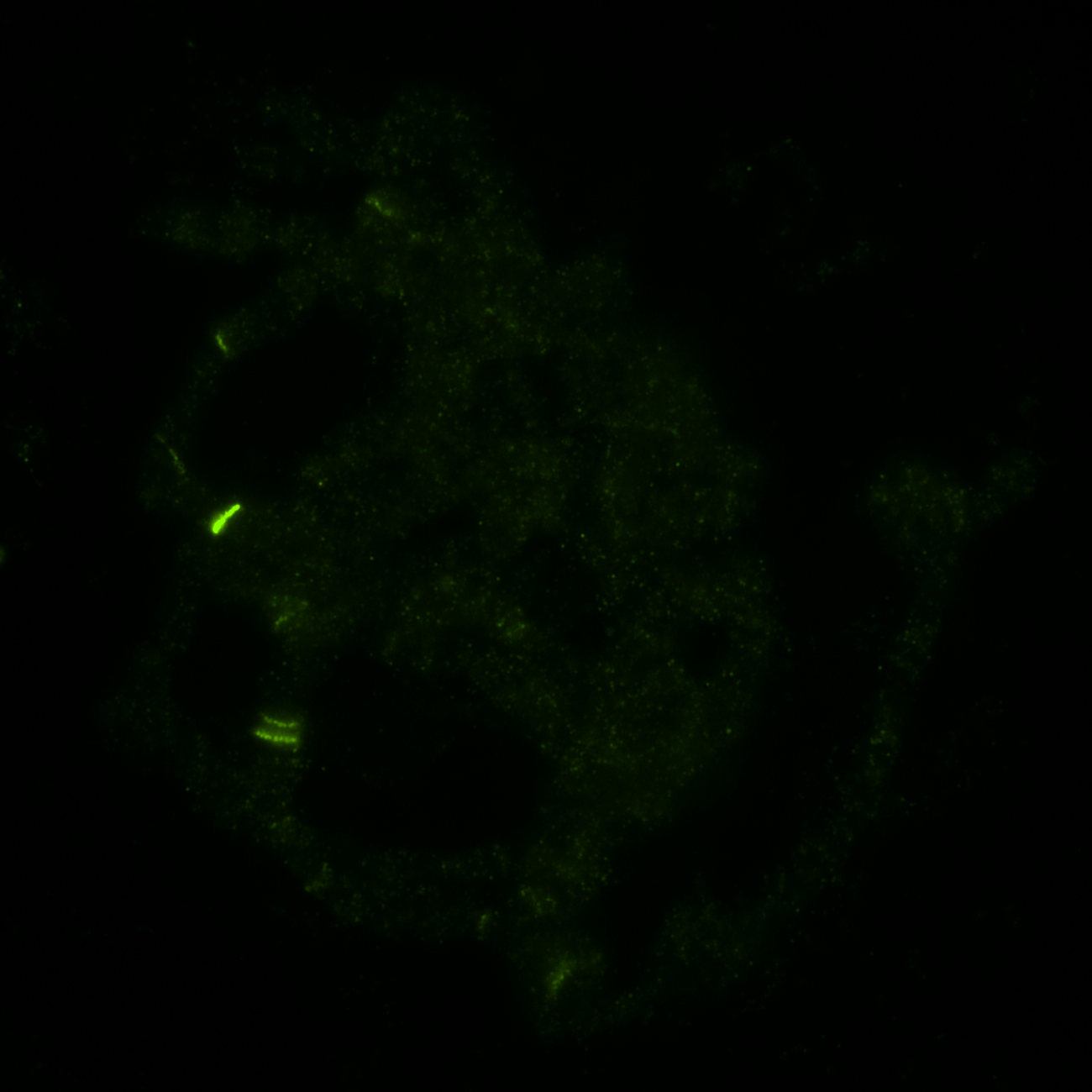

Supplement: Figure 2—source data 1. [file elife-93241-fig2-data1.zip › d(41-85)/20200310-msl1d41_85-6-1-F2.tif (RGB) (1).tif]

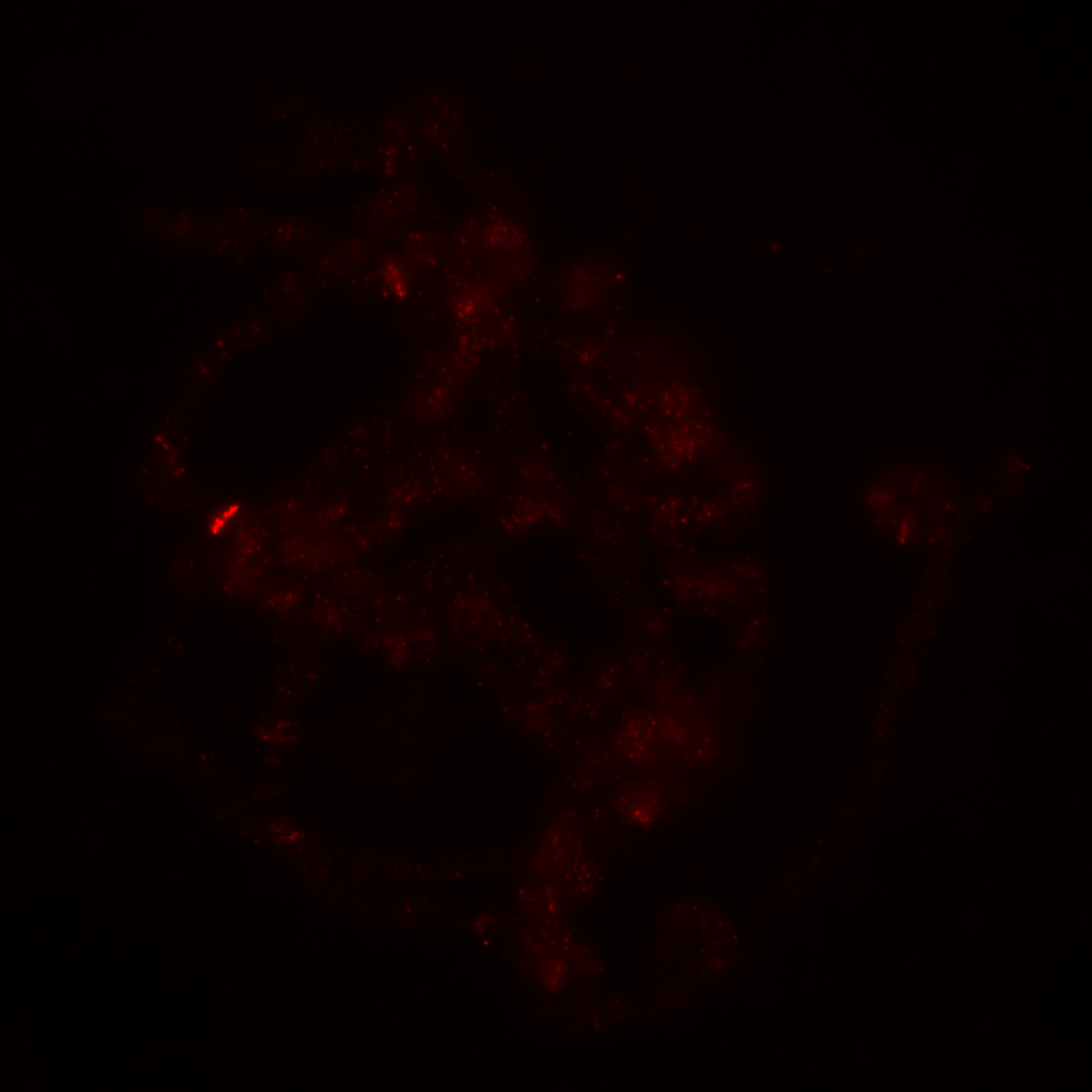

Supplement: Figure 2—source data 1. [file elife-93241-fig2-data1.zip › d(41-85)/20200310-msl1d41_85-6-1-T2.tif (RGB) (1).tif]

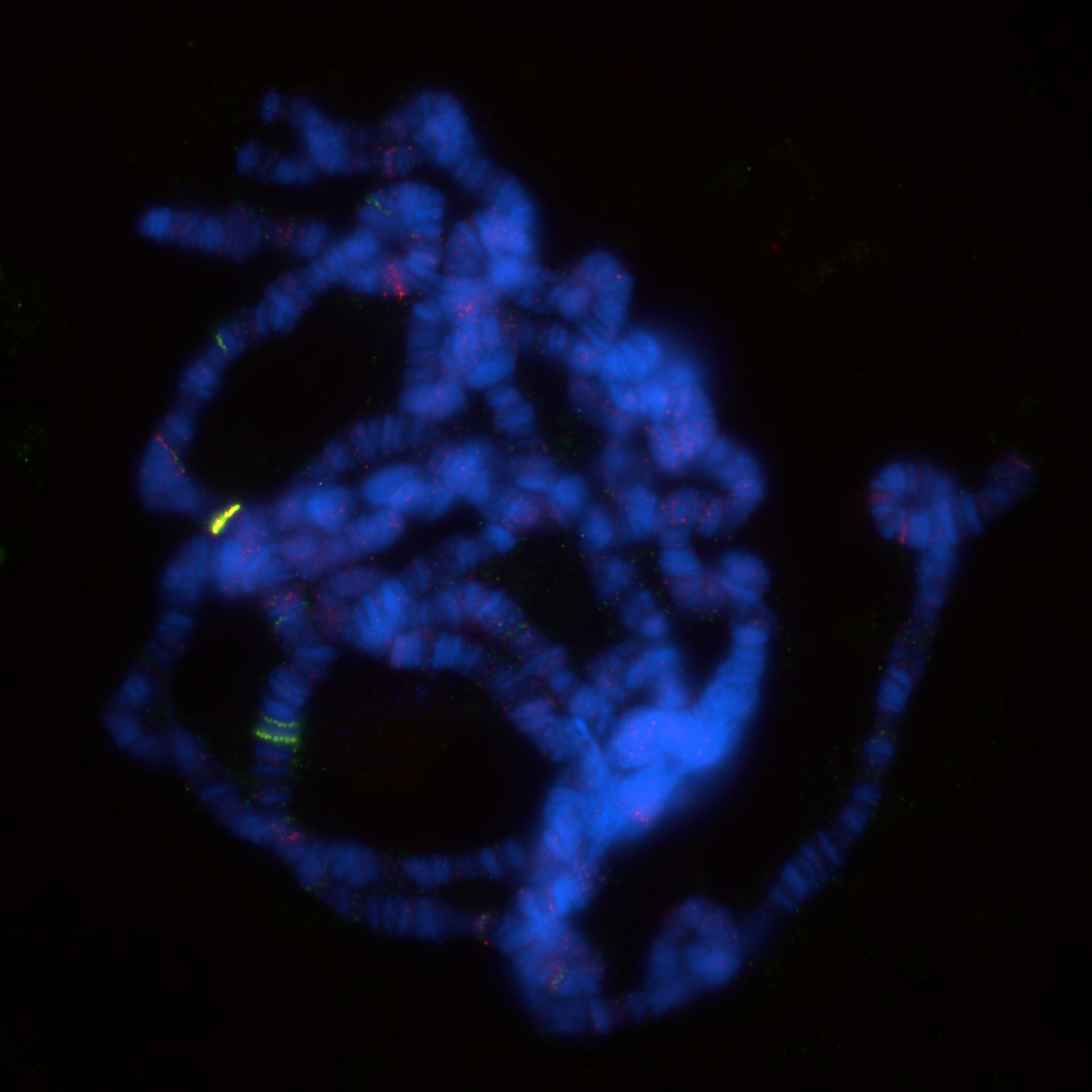

Supplement: Figure 2—source data 1. [file elife-93241-fig2-data1.zip › d(41-85)/Composite DFT.tif]

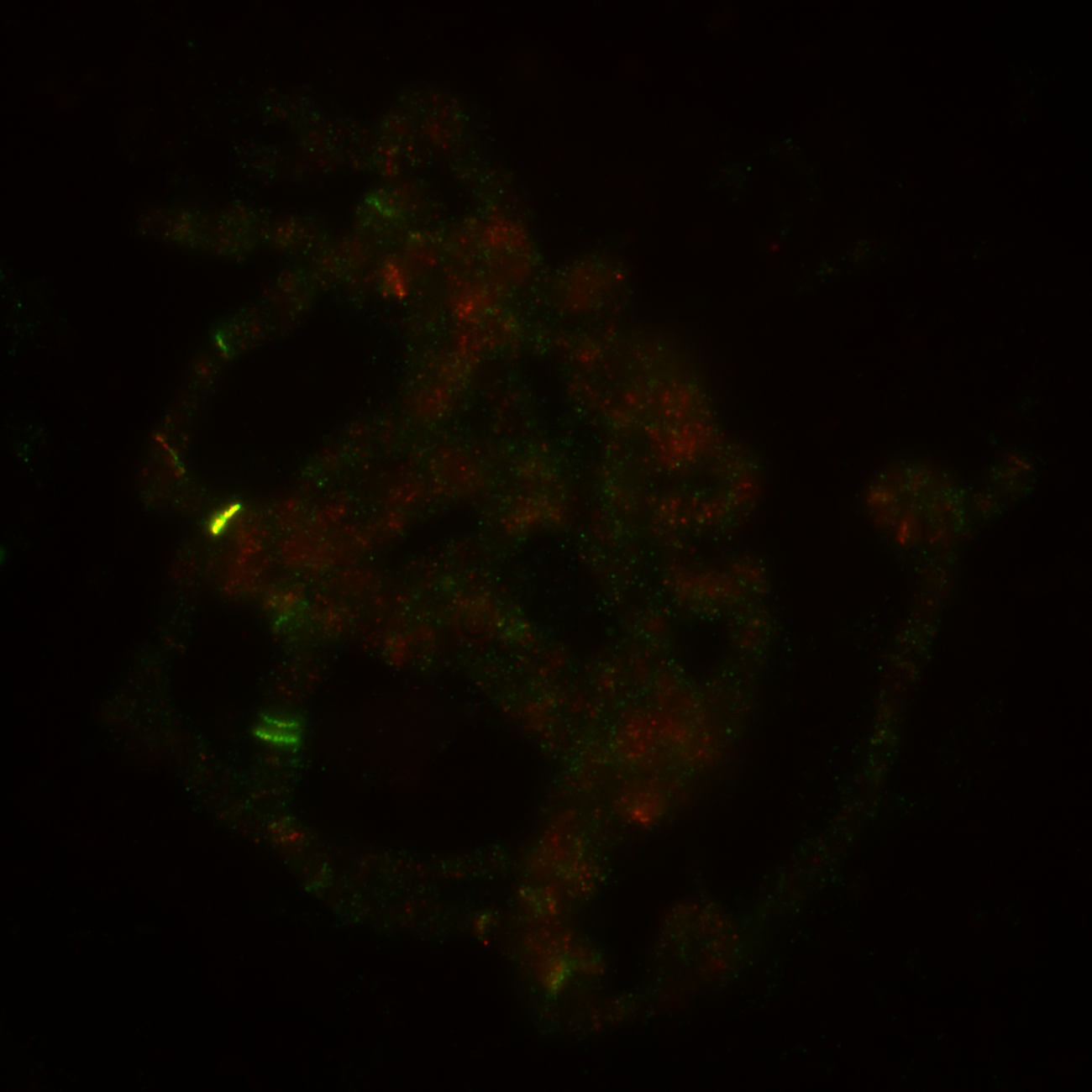

Supplement: Figure 2—source data 1. [file elife-93241-fig2-data1.zip › d(41-85)/Composite FT.tif]

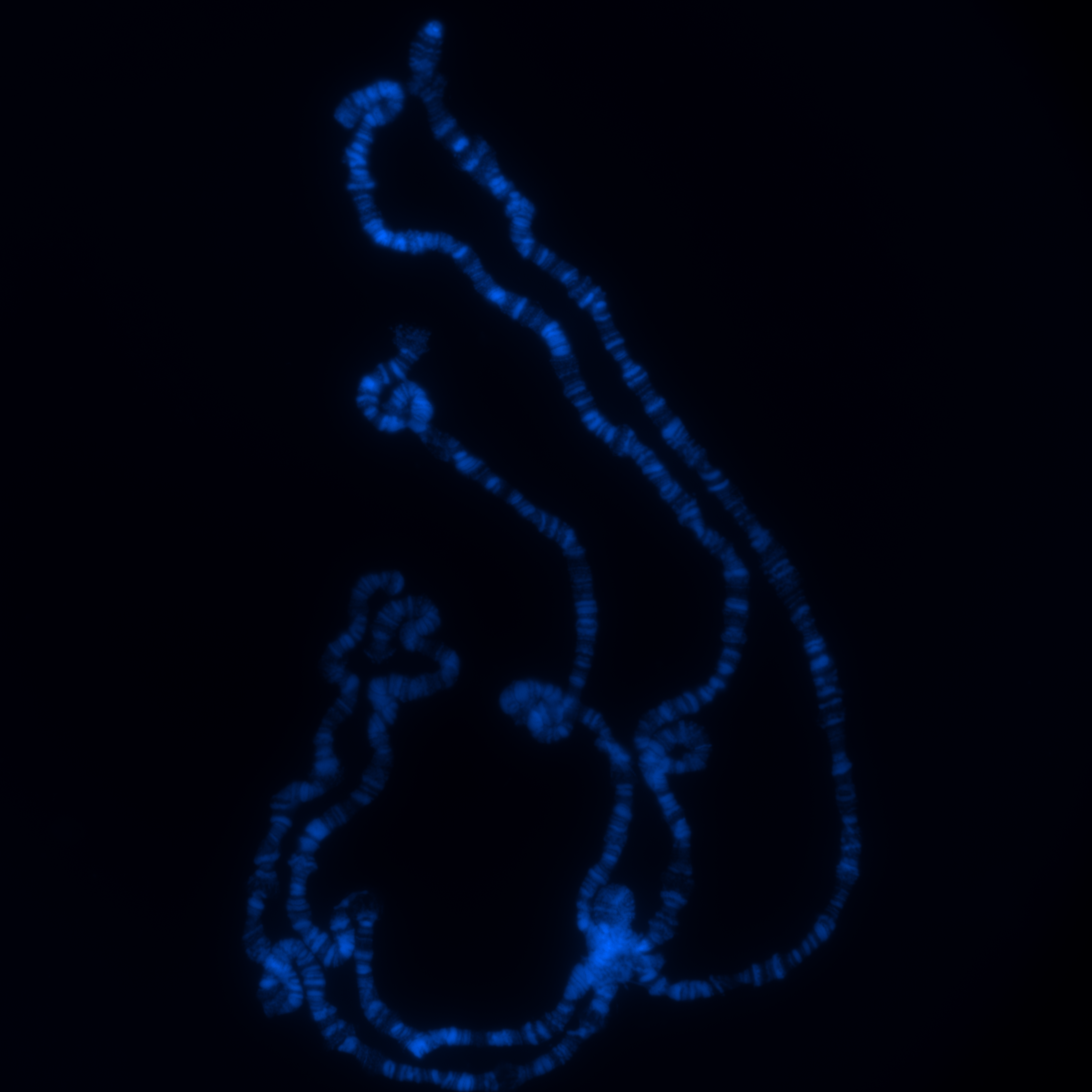

Supplement: Figure 2—source data 1. [file elife-93241-fig2-data1.zip › d(66-85)/2019-10-03_1-1_msl1_DAPI.tif (RGB).tif]

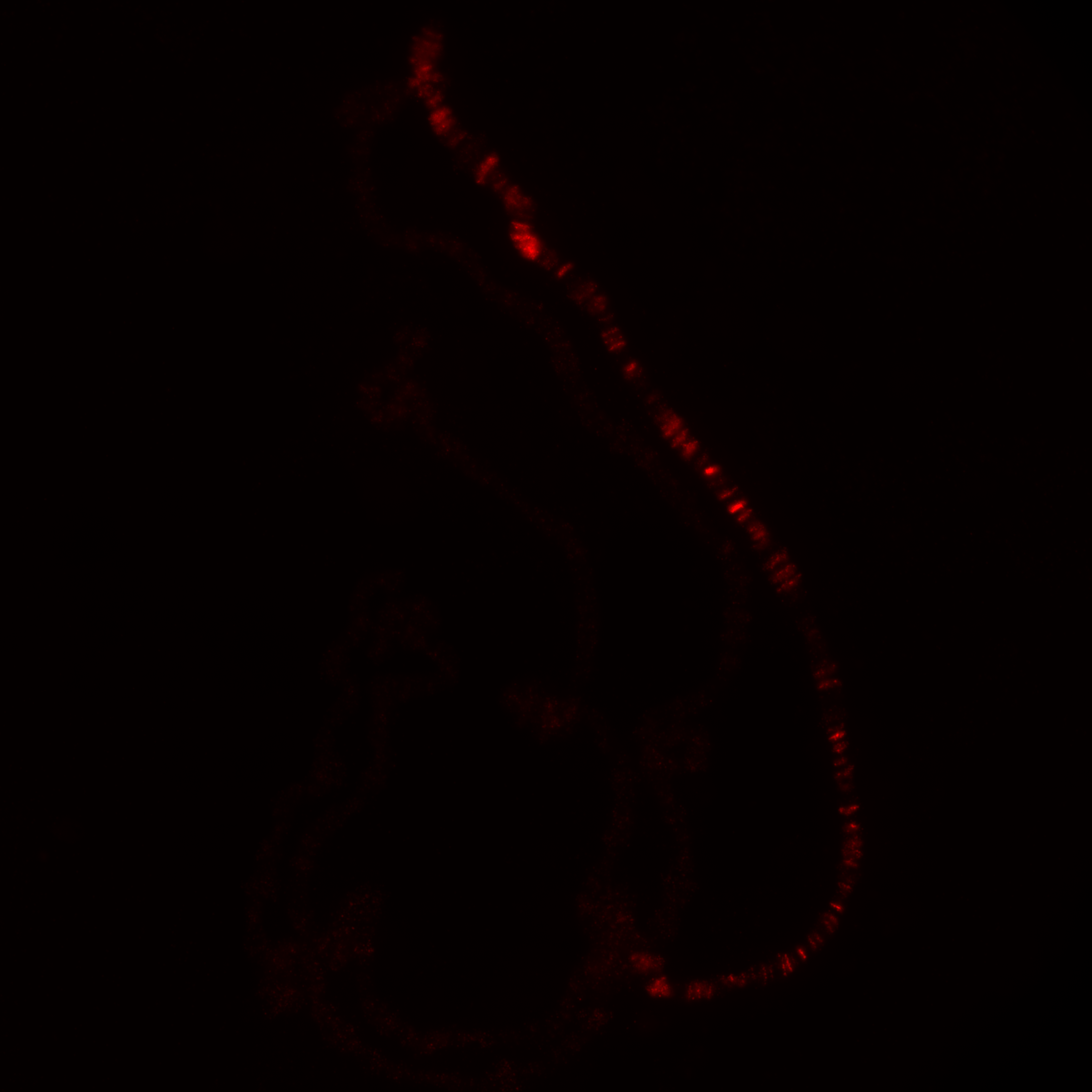

Supplement: Figure 2—source data 1. [file elife-93241-fig2-data1.zip › d(66-85)/2019-10-03_1-1_msl1_FLAG.tif]

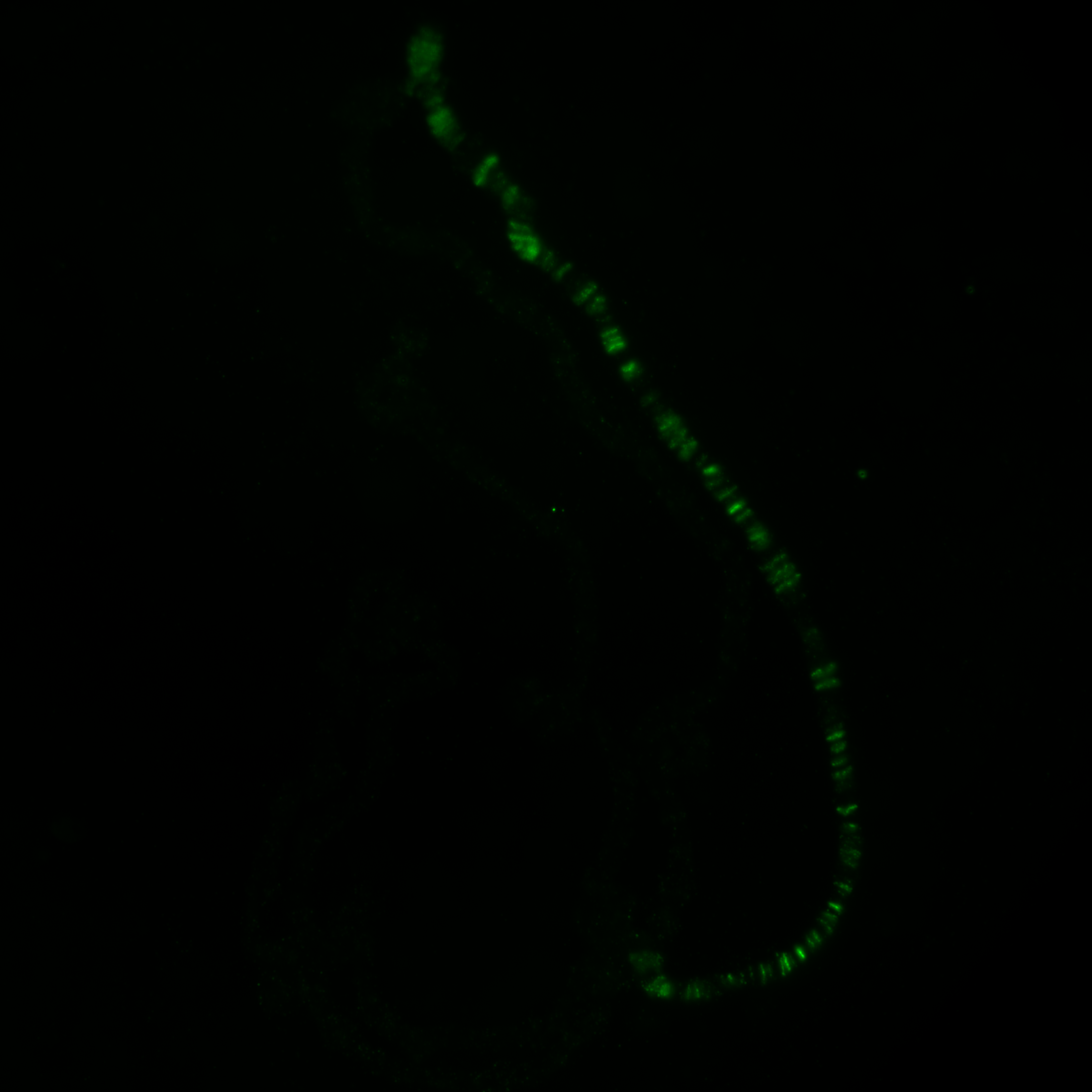

Supplement: Figure 2—source data 1. [file elife-93241-fig2-data1.zip › d(66-85)/2019-10-03_1-1_msl1_MSL1.tif]

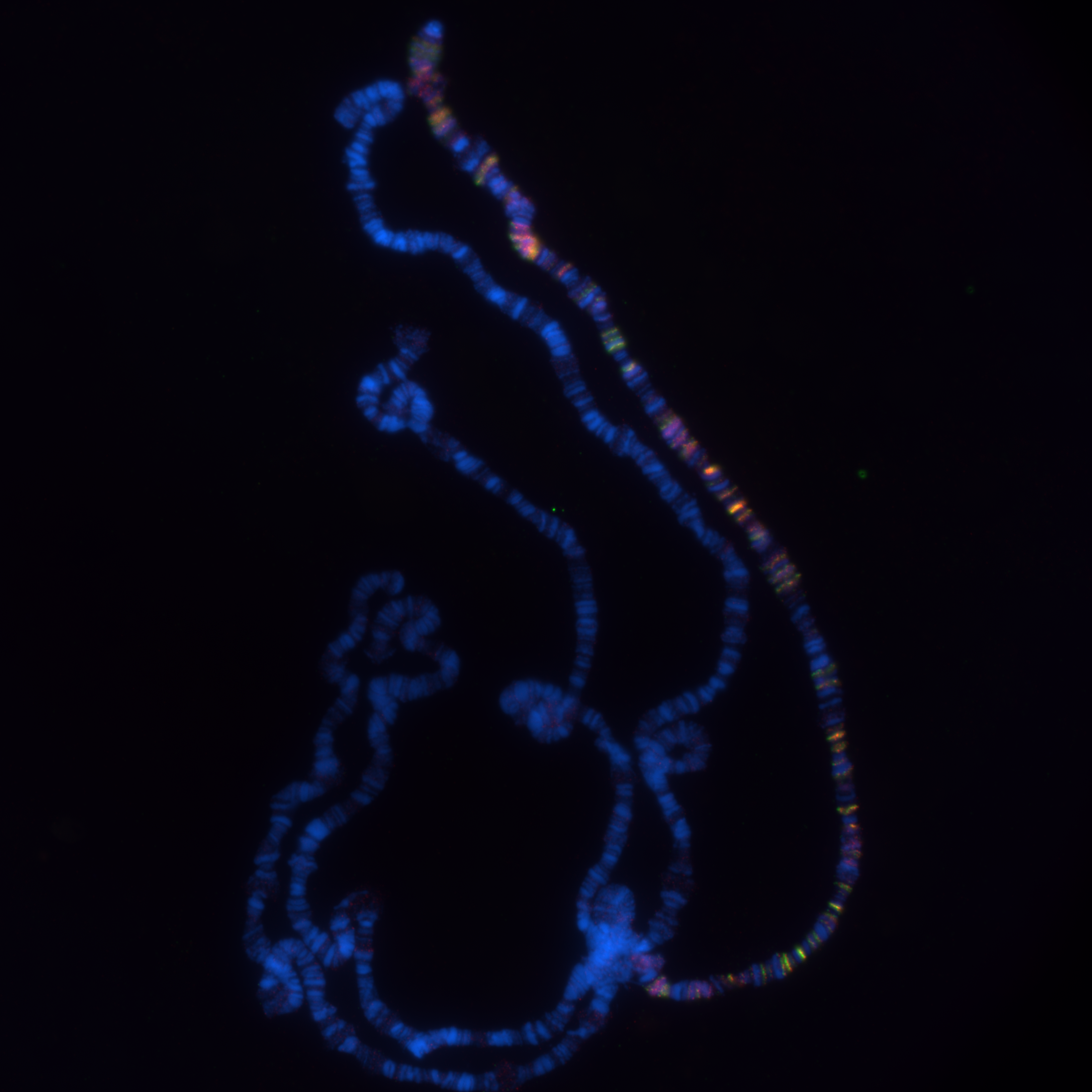

Supplement: Figure 2—source data 1. [file elife-93241-fig2-data1.zip › d(66-85)/Composite DFT.tif]

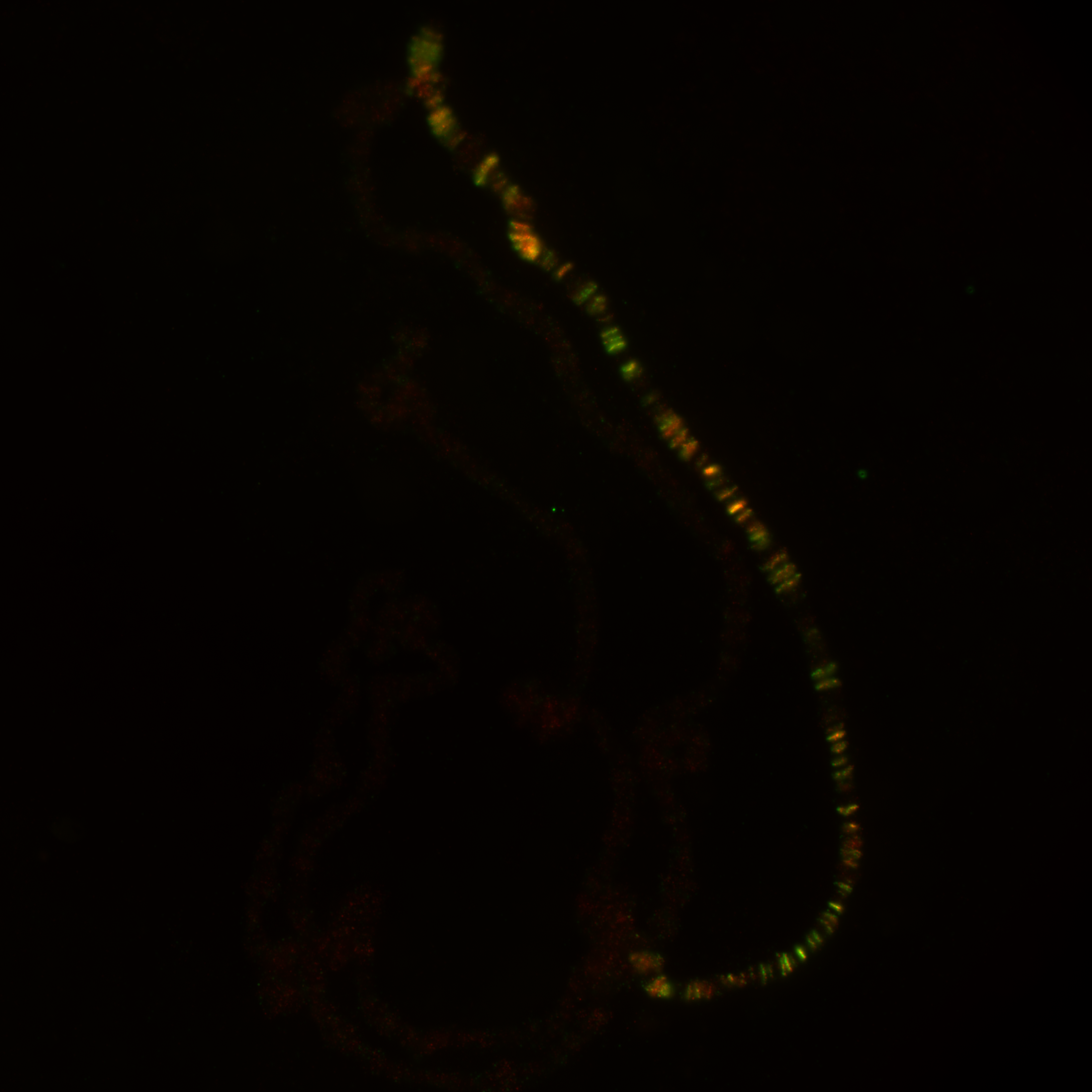

Supplement: Figure 2—source data 1. [file elife-93241-fig2-data1.zip › d(66-85)/Composite FT.tif]

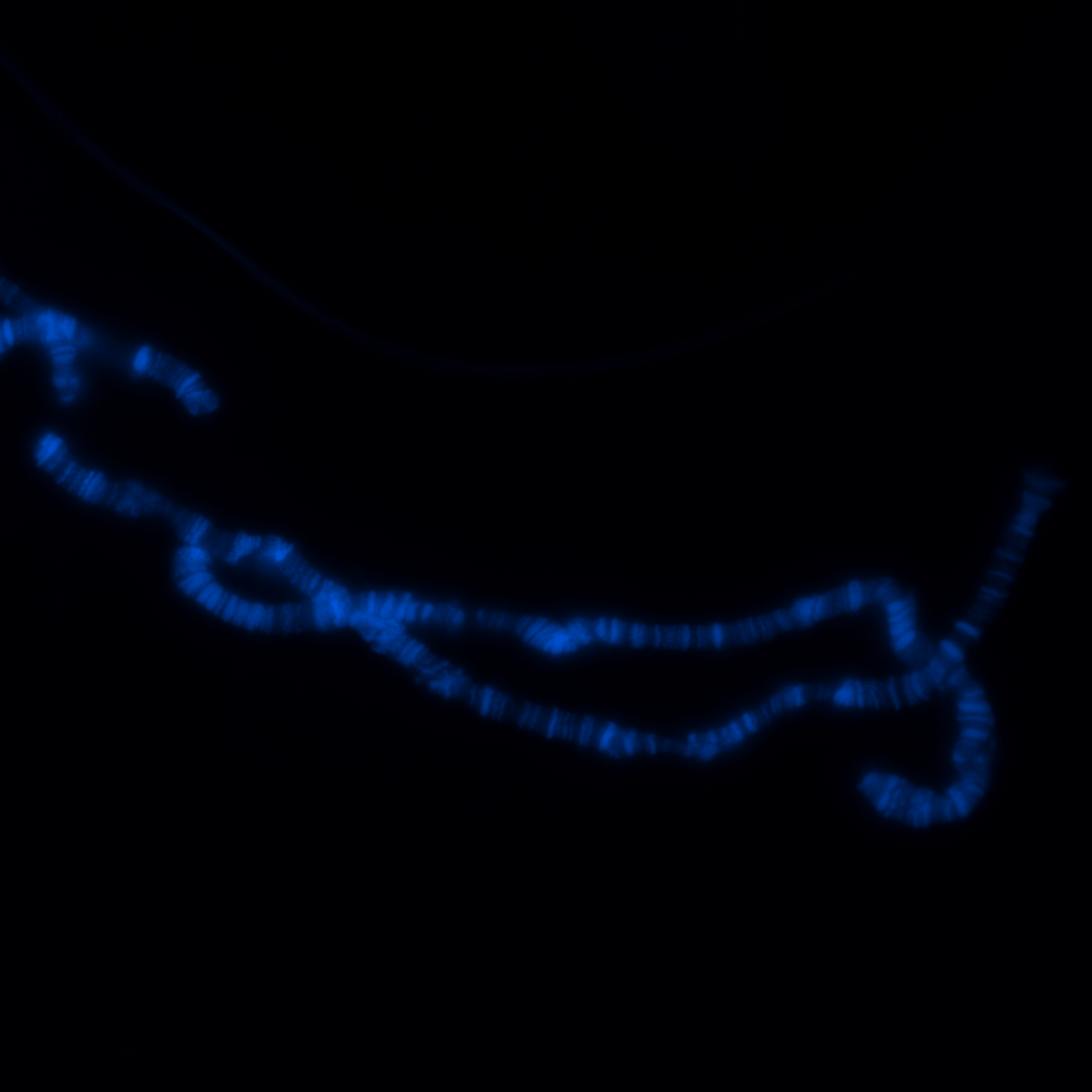

Supplement: Figure 2—source data 1. [file elife-93241-fig2-data1.zip › d(8-20)/2022-02-25-d(8-20)-3-1c1crop.tif]

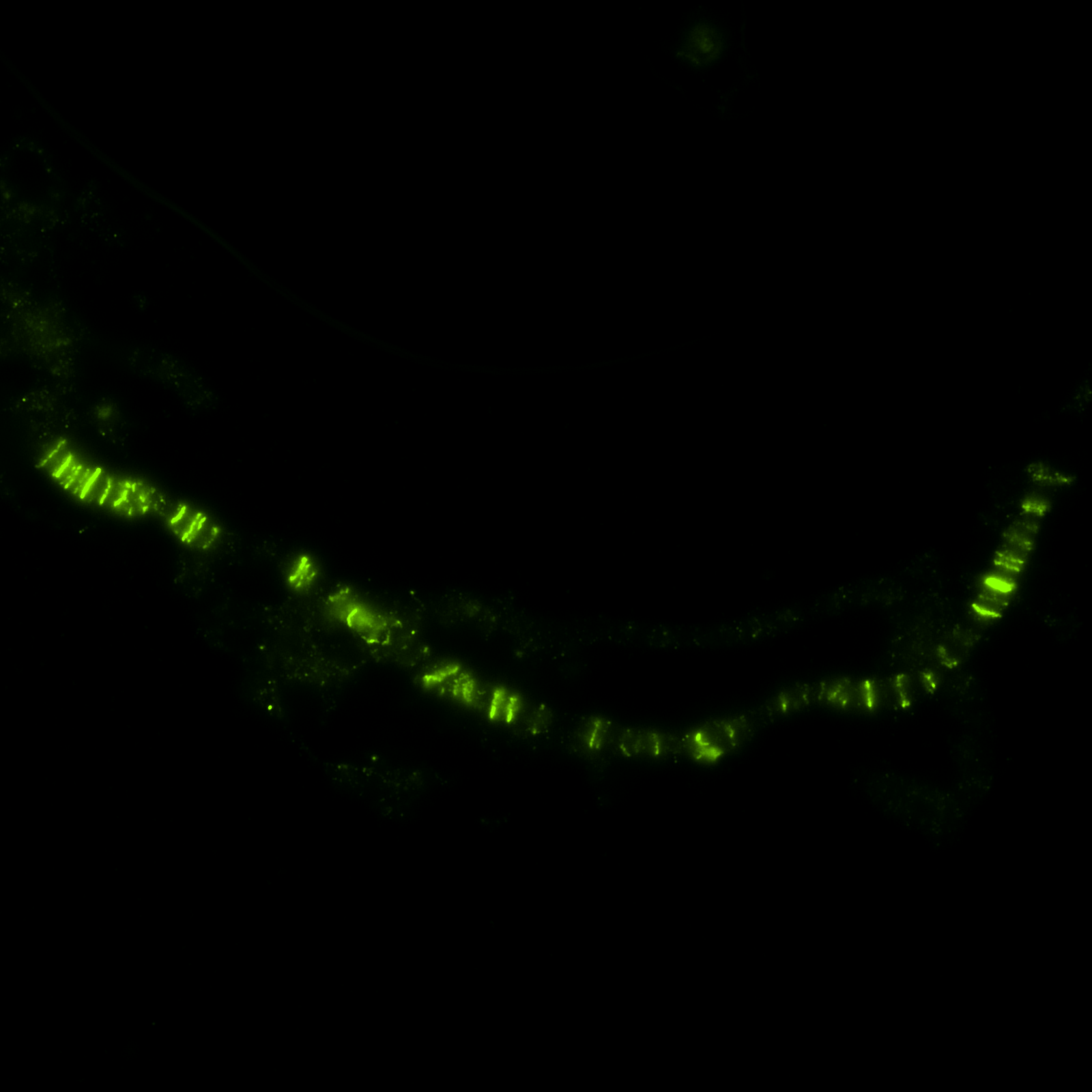

Supplement: Figure 2—source data 1. [file elife-93241-fig2-data1.zip › d(8-20)/2022-02-25-d(8-20)-3-1c2crop.tif]

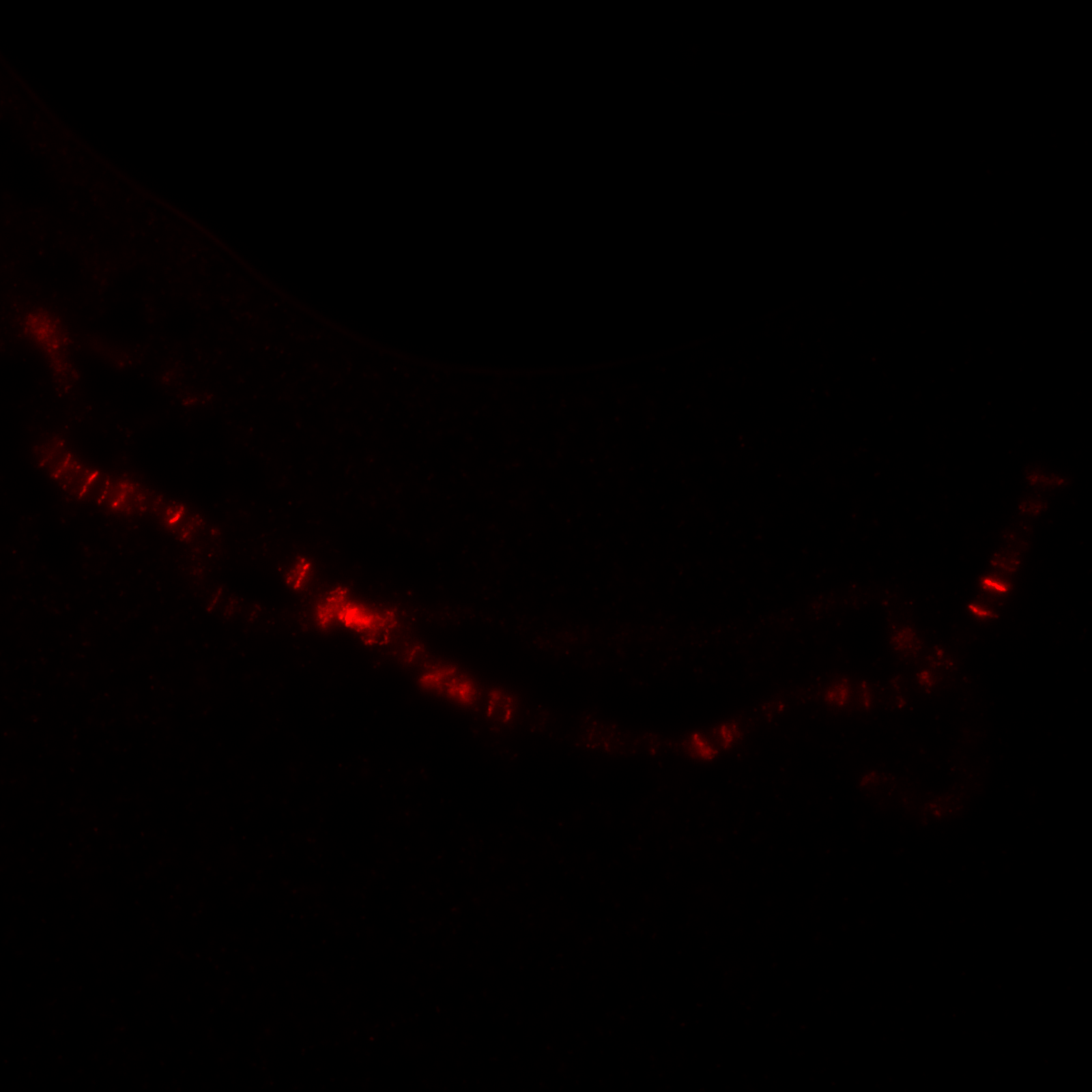

Supplement: Figure 2—source data 1. [file elife-93241-fig2-data1.zip › d(8-20)/2022-02-25-d(8-20)-3-1c3crop.tif]

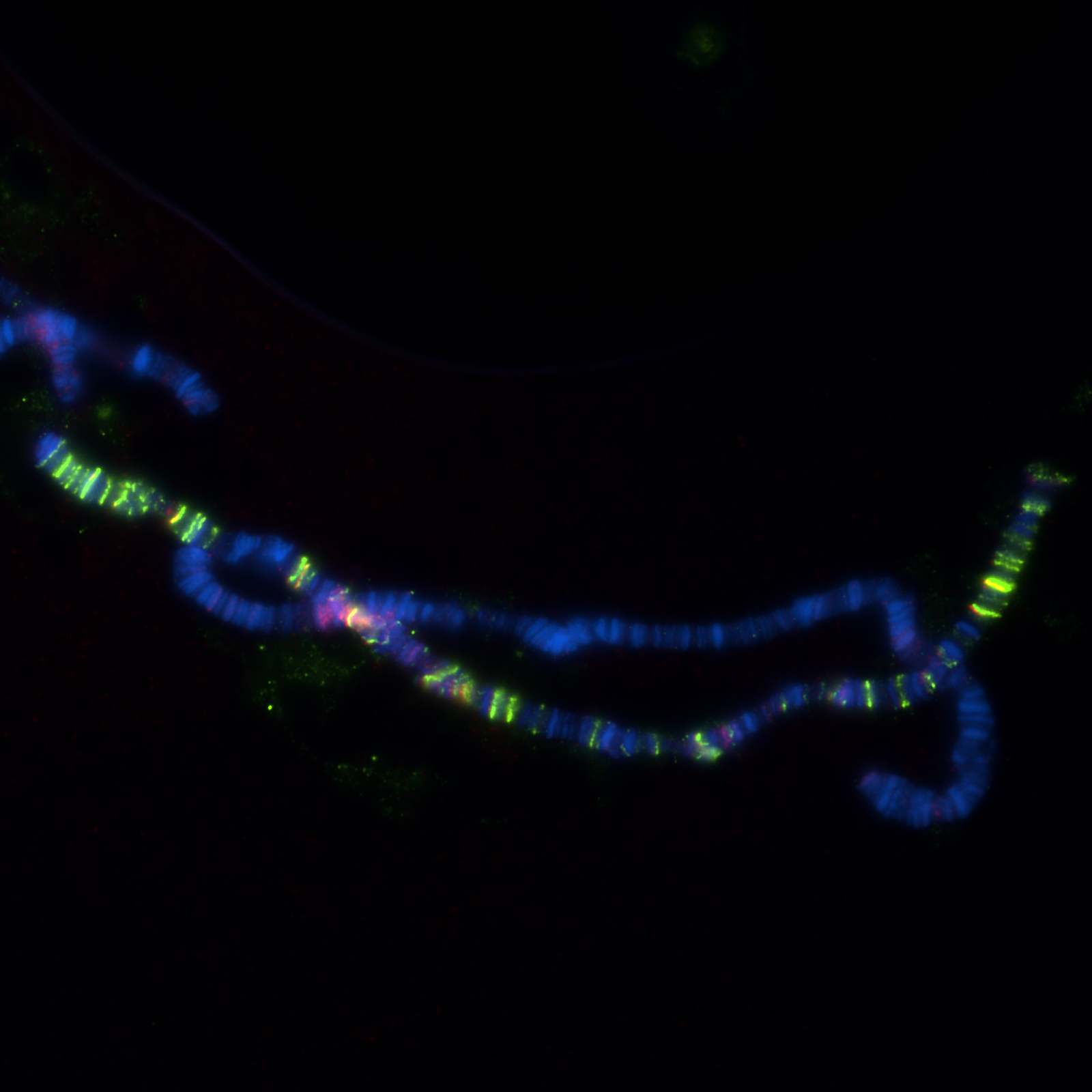

Supplement: Figure 2—source data 1. [file elife-93241-fig2-data1.zip › d(8-20)/Composite DFT.tif]

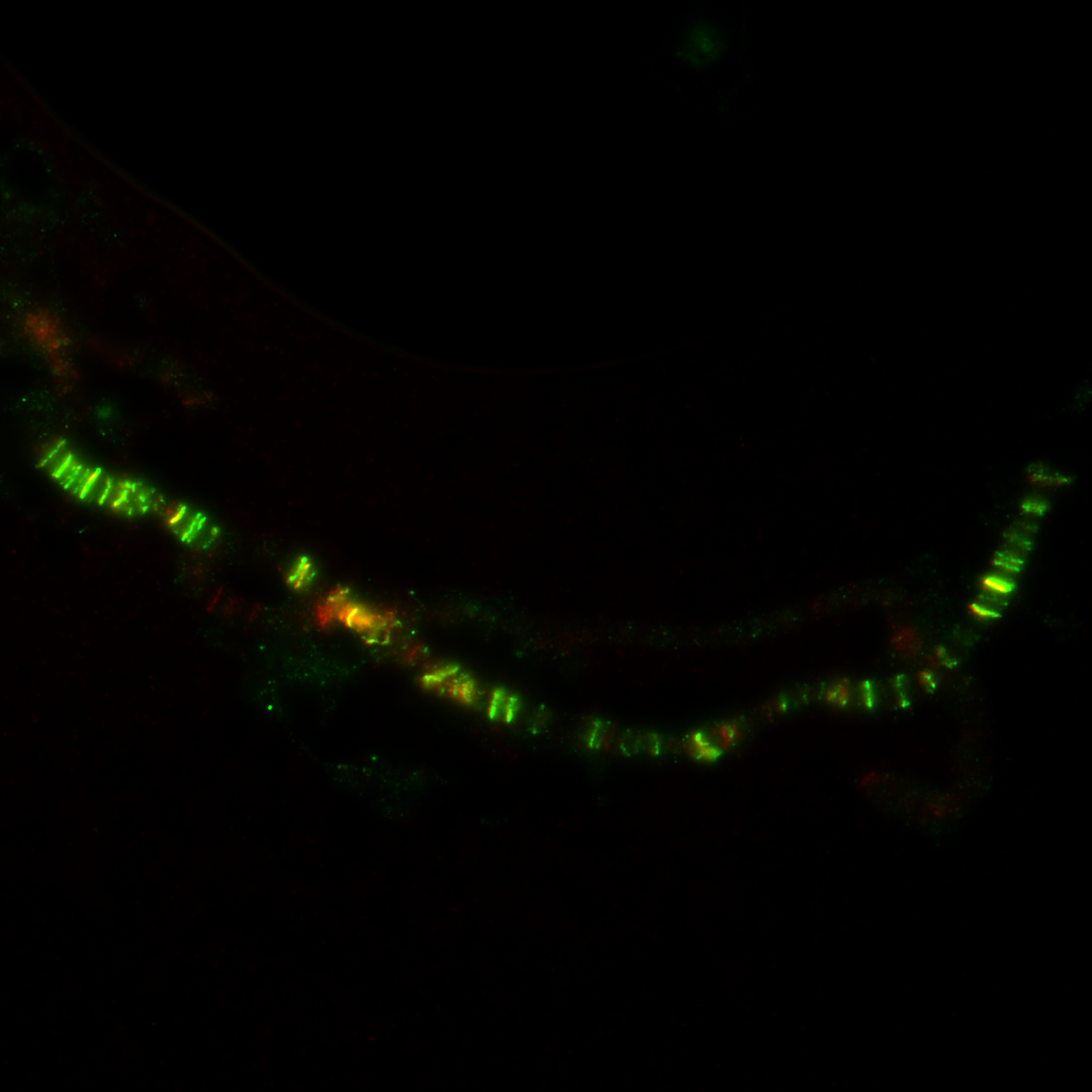

Supplement: Figure 2—source data 1. [file elife-93241-fig2-data1.zip › d(8-20)/Composite FT.tif]

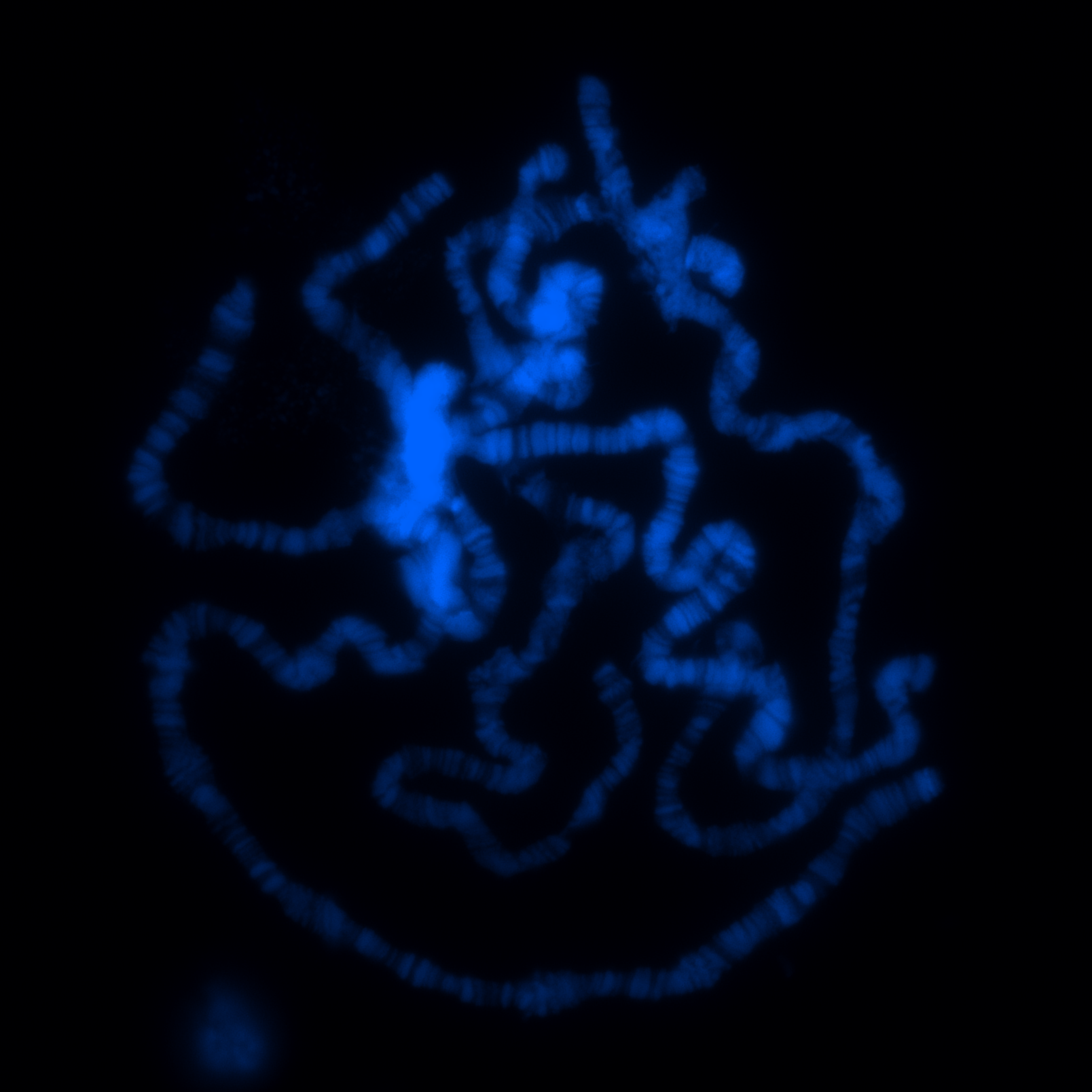

Supplement: Figure 2—source data 1. [file elife-93241-fig2-data1.zip › d15/20200310-msl1d15-2-3-D.tif (RGB).tif]

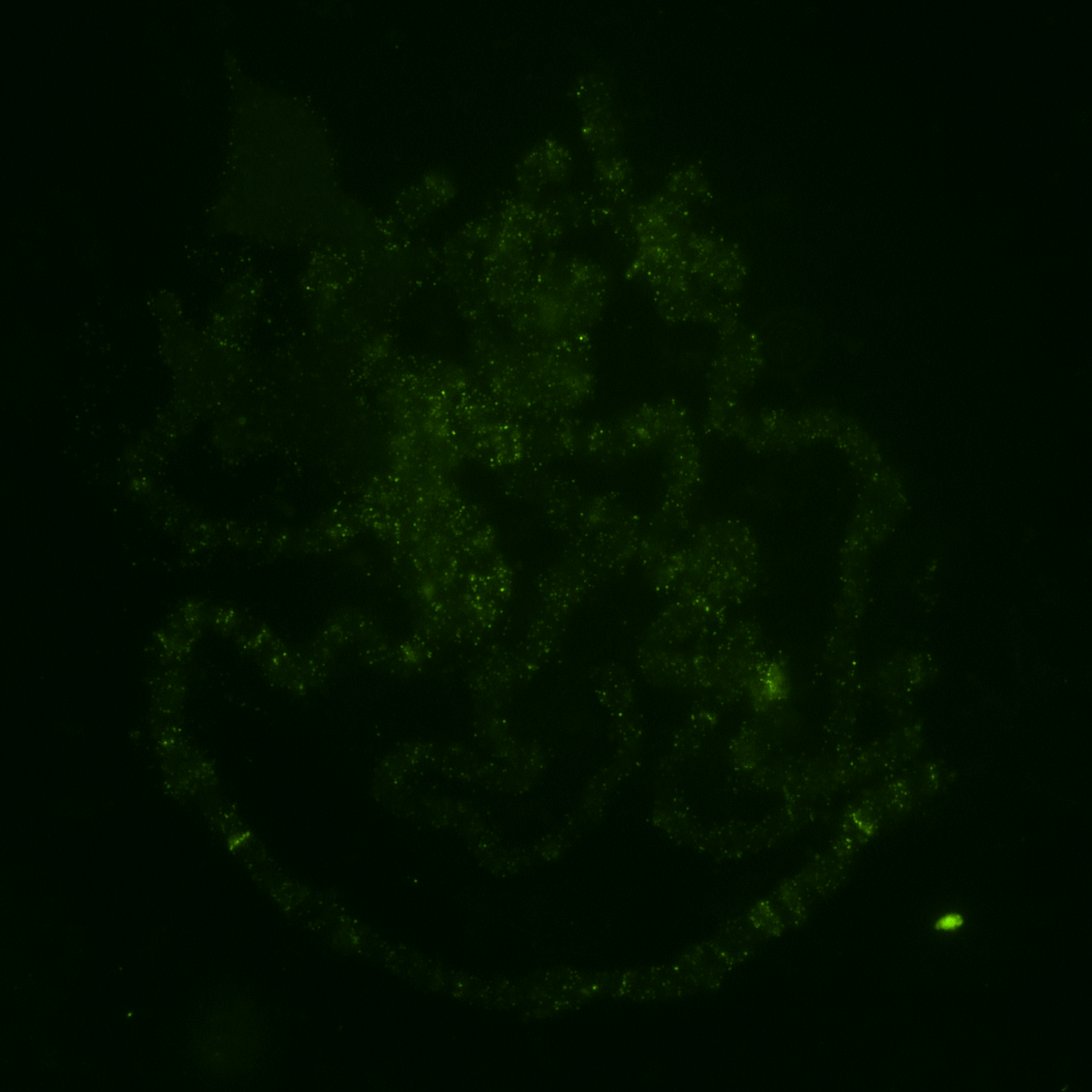

Supplement: Figure 2—source data 1. [file elife-93241-fig2-data1.zip › d15/20200310-msl1d15-2-3-F.tif (RGB).tif]

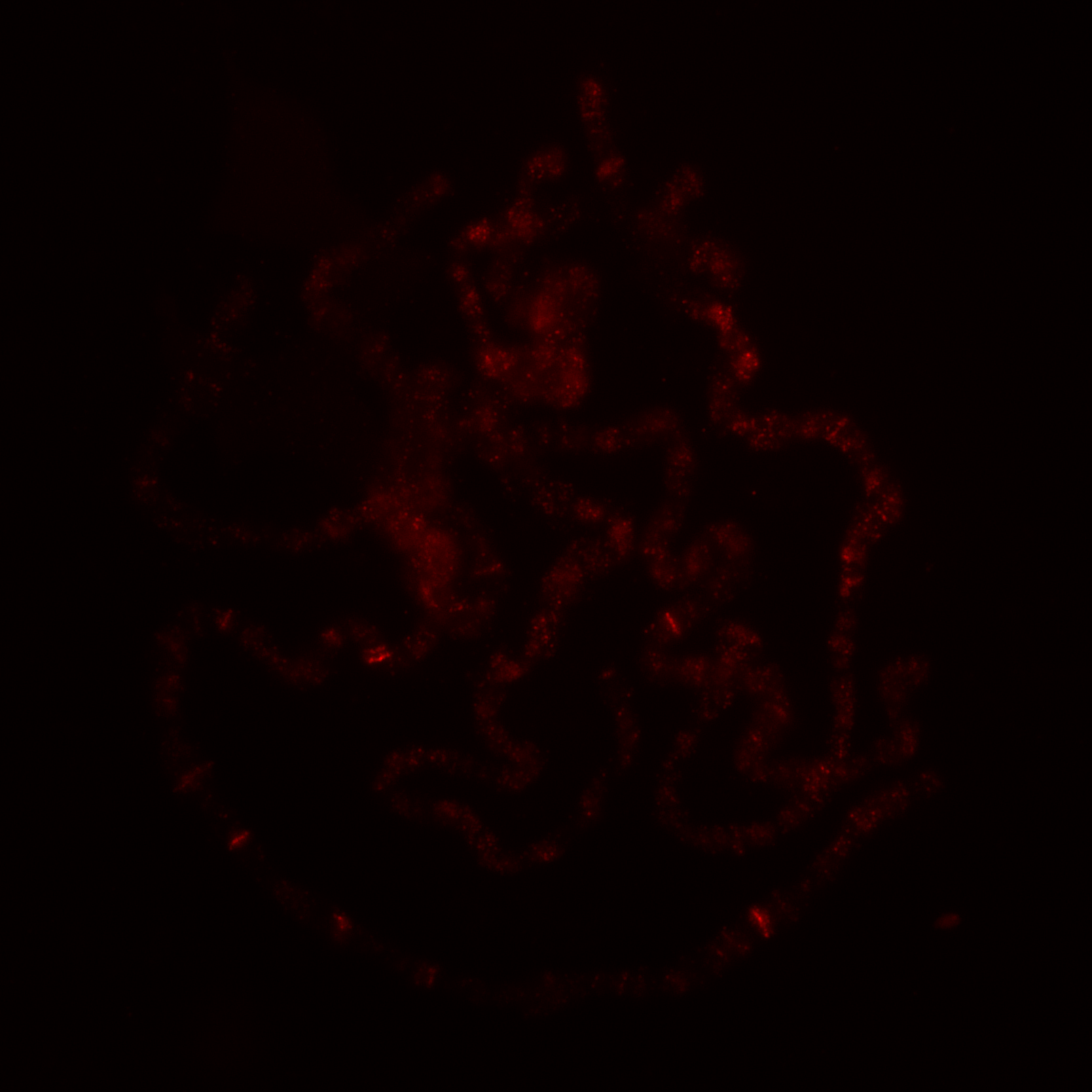

Supplement: Figure 2—source data 1. [file elife-93241-fig2-data1.zip › d15/20200310-msl1d15-2-3-T.tif (RGB).tif]

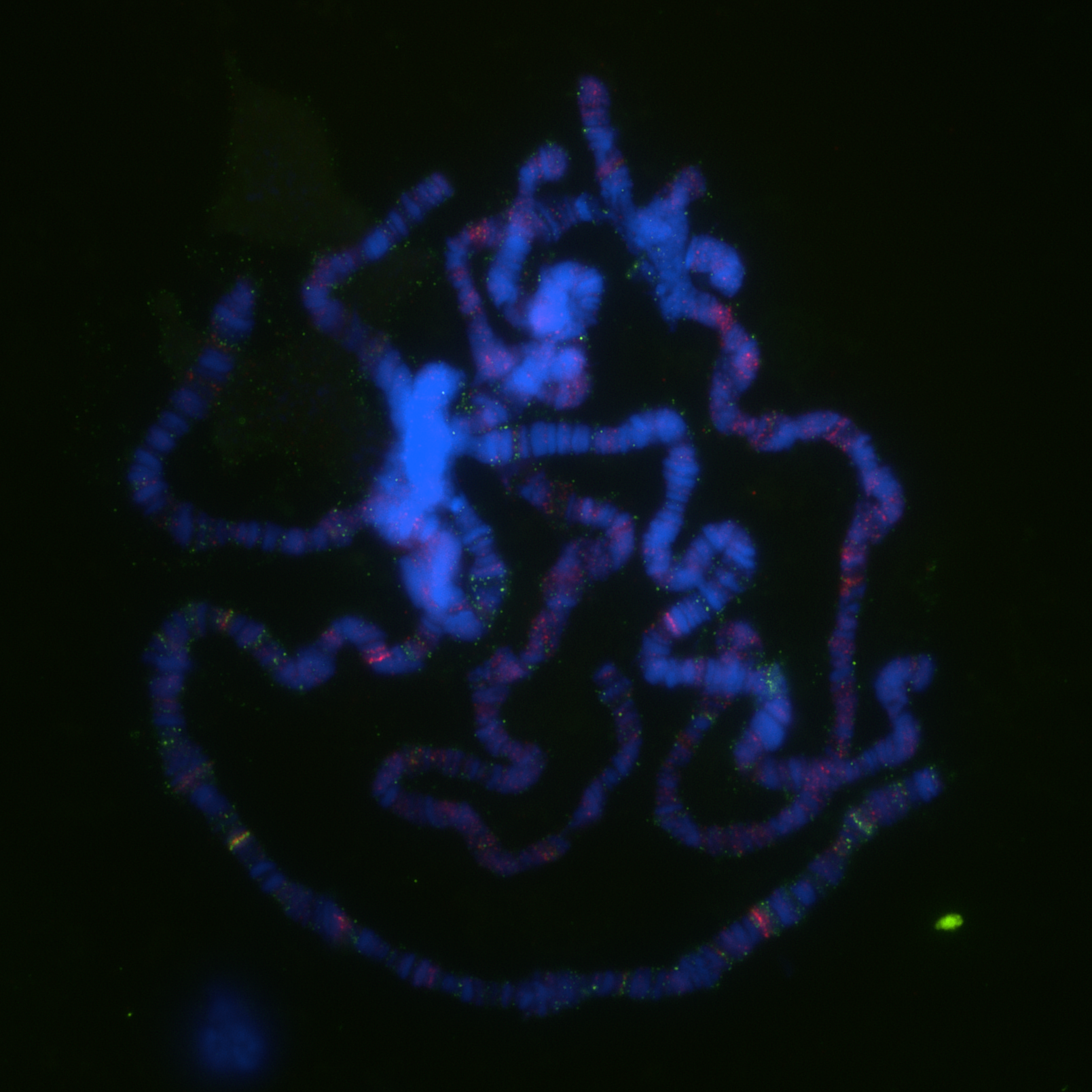

Supplement: Figure 2—source data 1. [file elife-93241-fig2-data1.zip › d15/Composite DFT.tif]

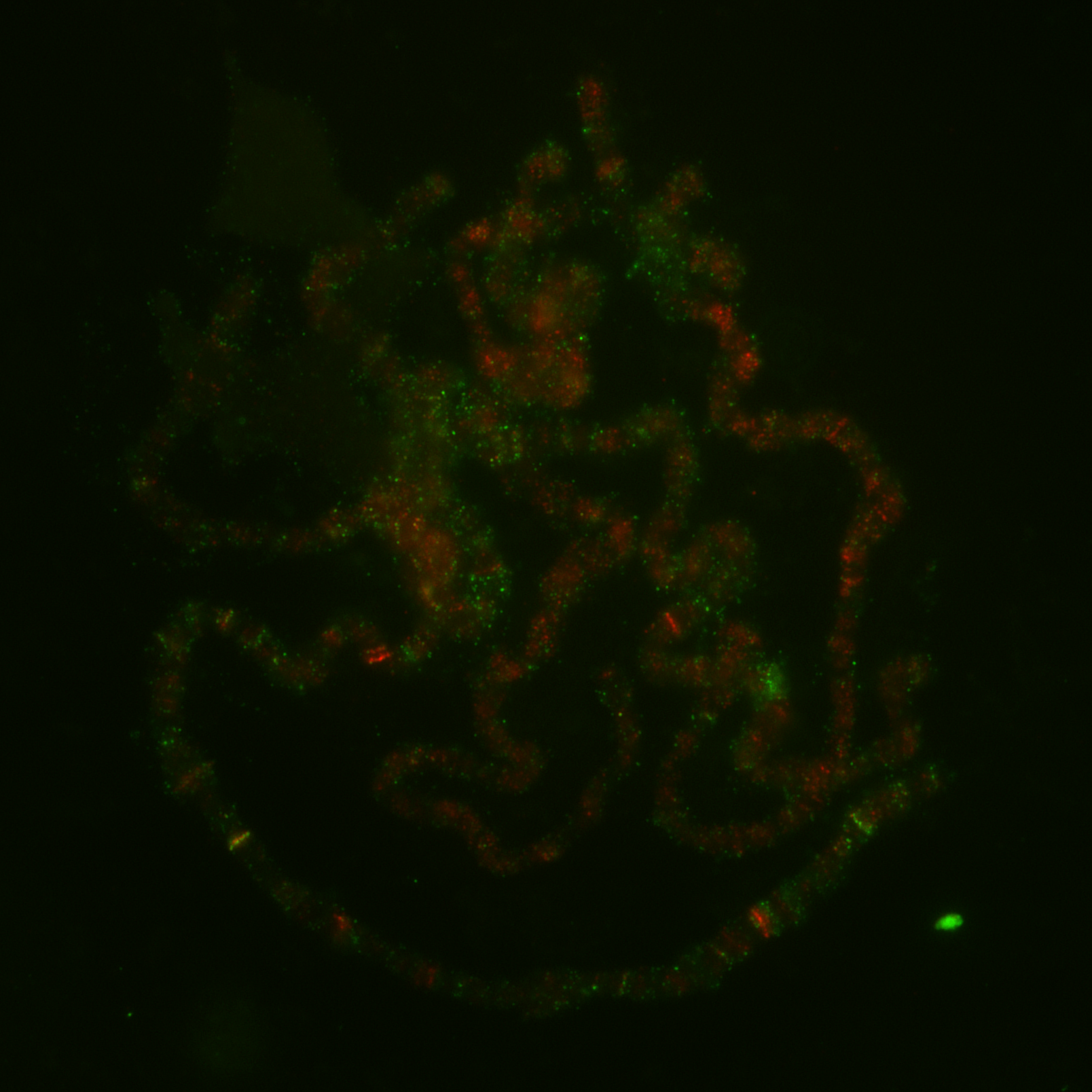

Supplement: Figure 2—source data 1. [file elife-93241-fig2-data1.zip › d15/Composite FT.tif]

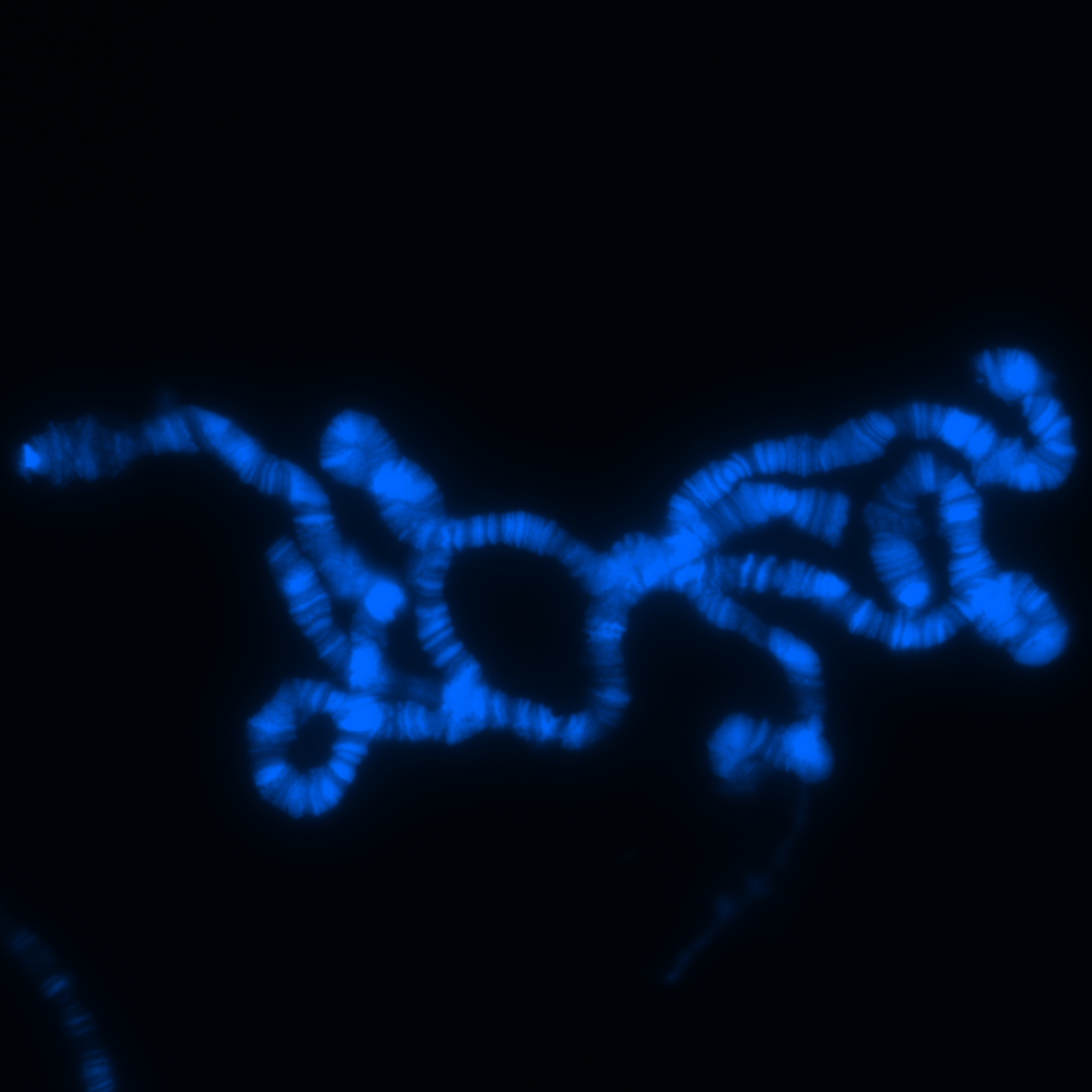

Supplement: Figure 2—source data 1. [file elife-93241-fig2-data1.zip › d85/2020-04-15-d84-2-1-Dcr.tif (RGB).tif]

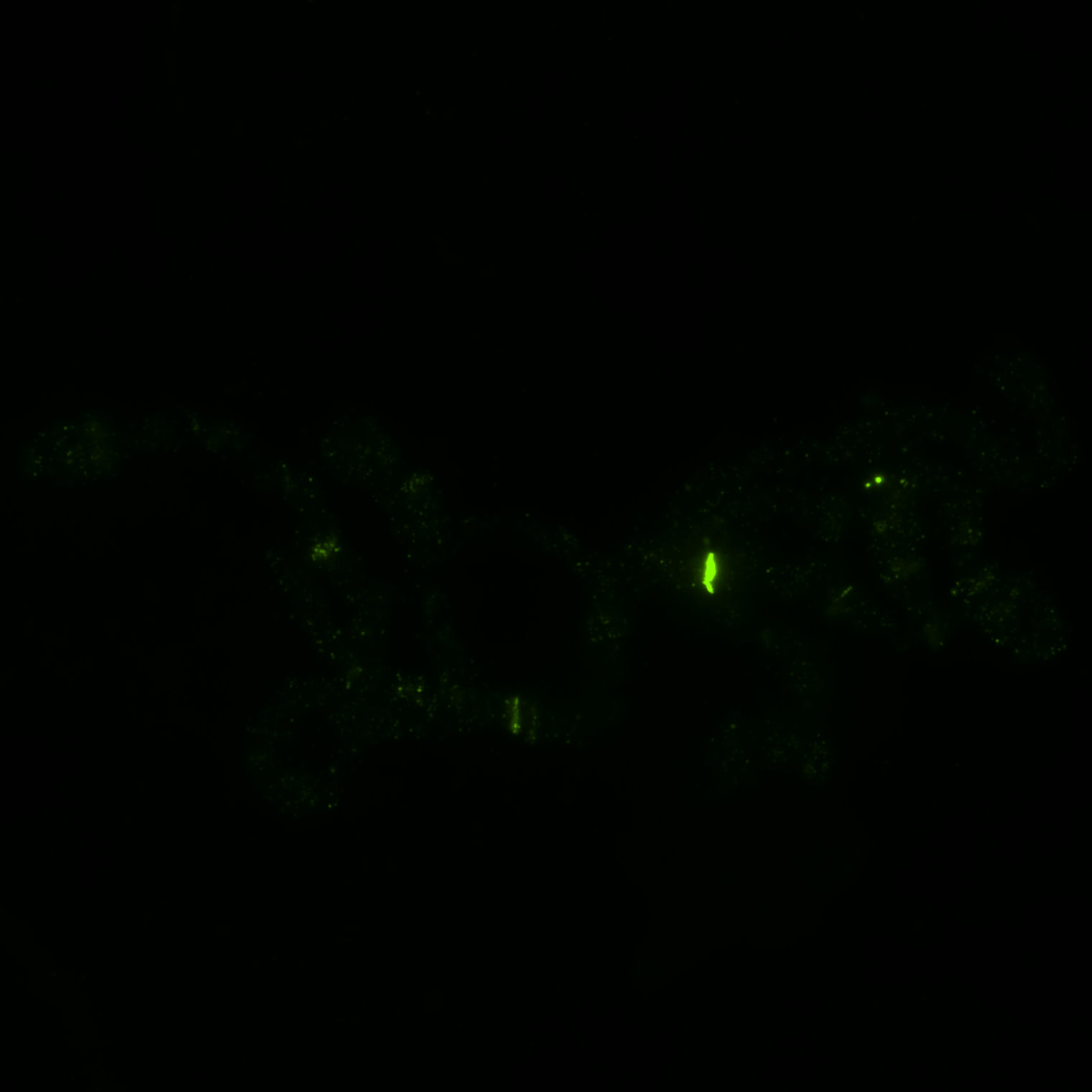

Supplement: Figure 2—source data 1. [file elife-93241-fig2-data1.zip › d85/2020-04-15-d84-2-1-Fcr.tif (RGB).tif]

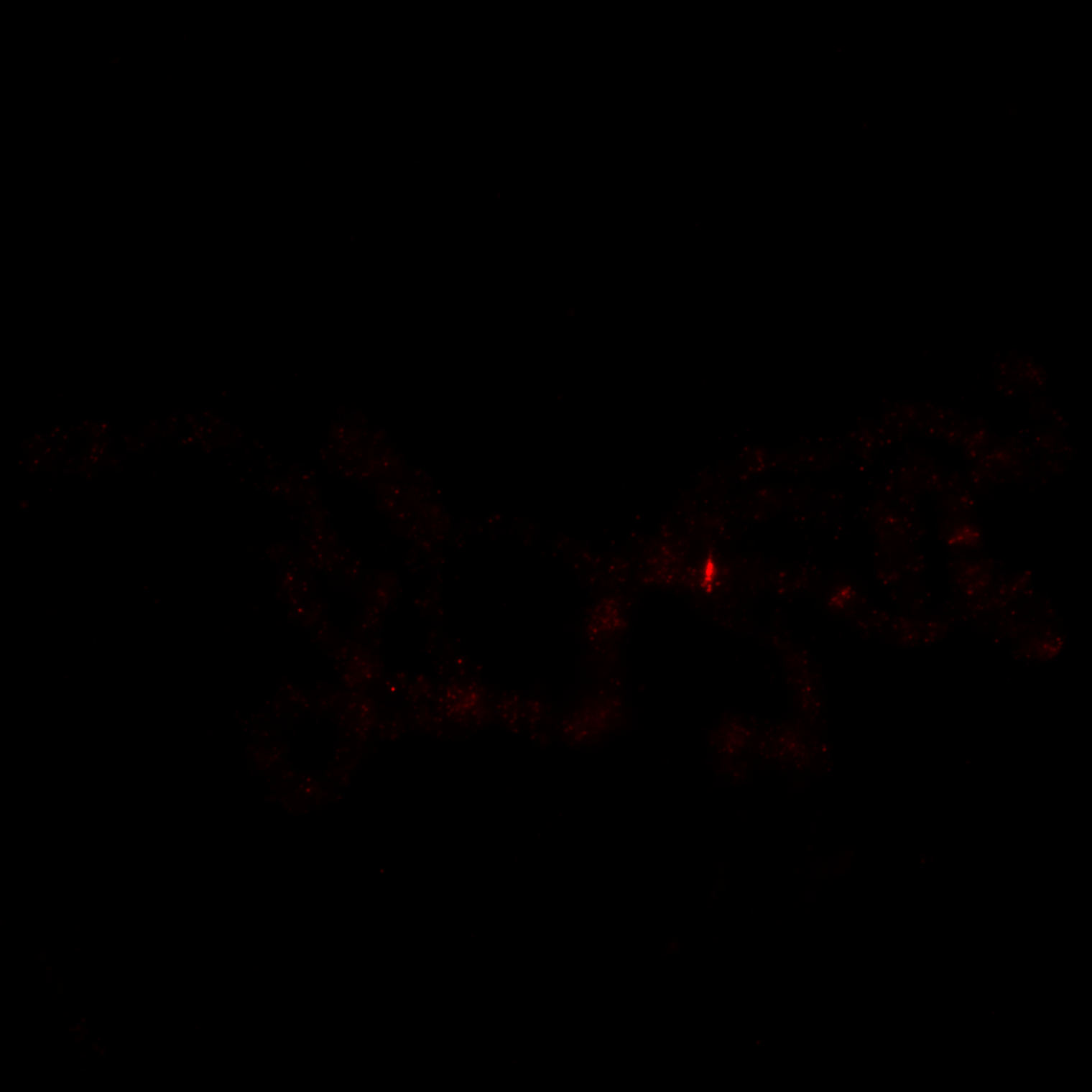

Supplement: Figure 2—source data 1. [file elife-93241-fig2-data1.zip › d85/2020-04-15-d84-2-1-Tcr.tif (RGB).tif]

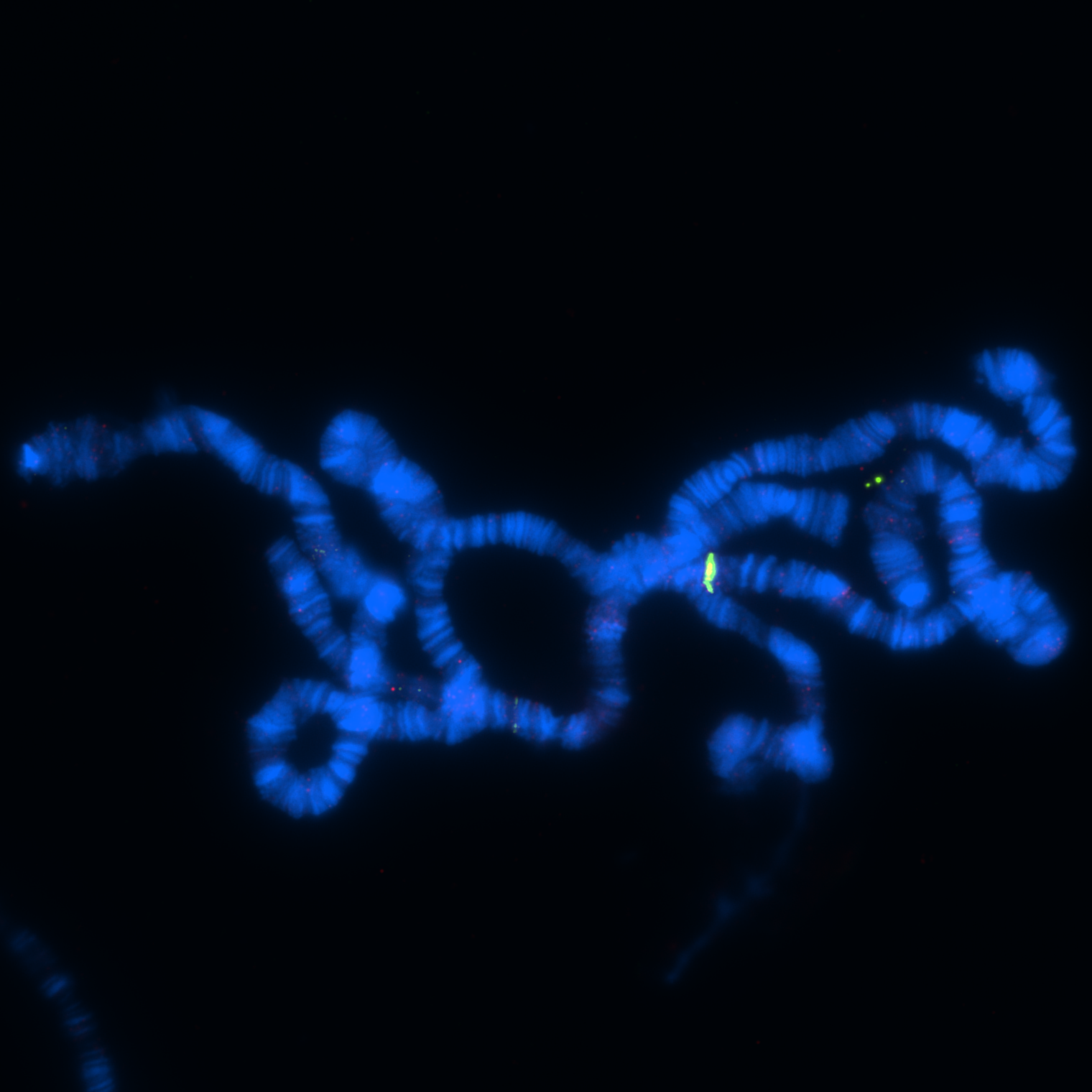

Supplement: Figure 2—source data 1. [file elife-93241-fig2-data1.zip › d85/Composite DFT.tif]

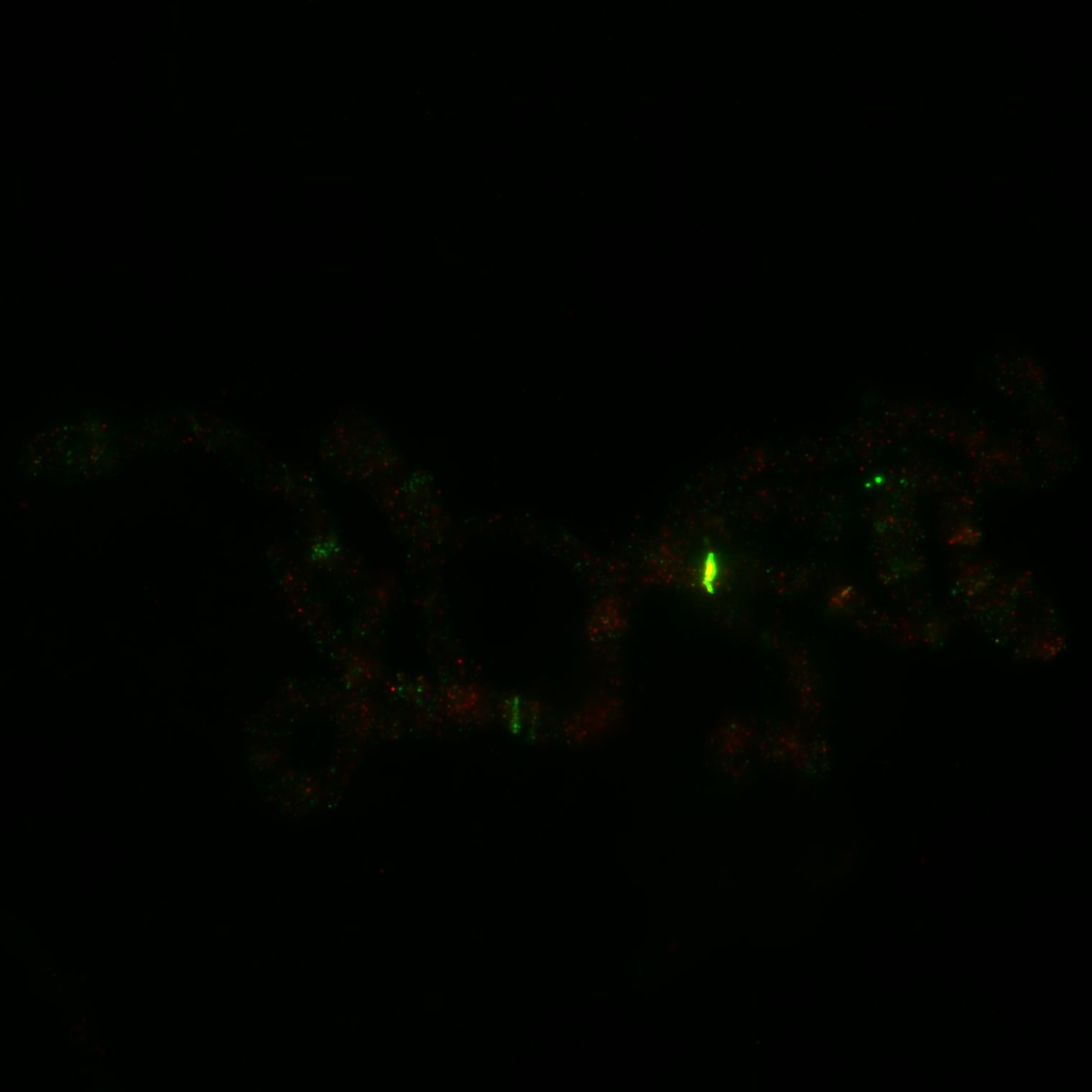

Supplement: Figure 2—source data 1. [file elife-93241-fig2-data1.zip › d85/Composite FT.tif]

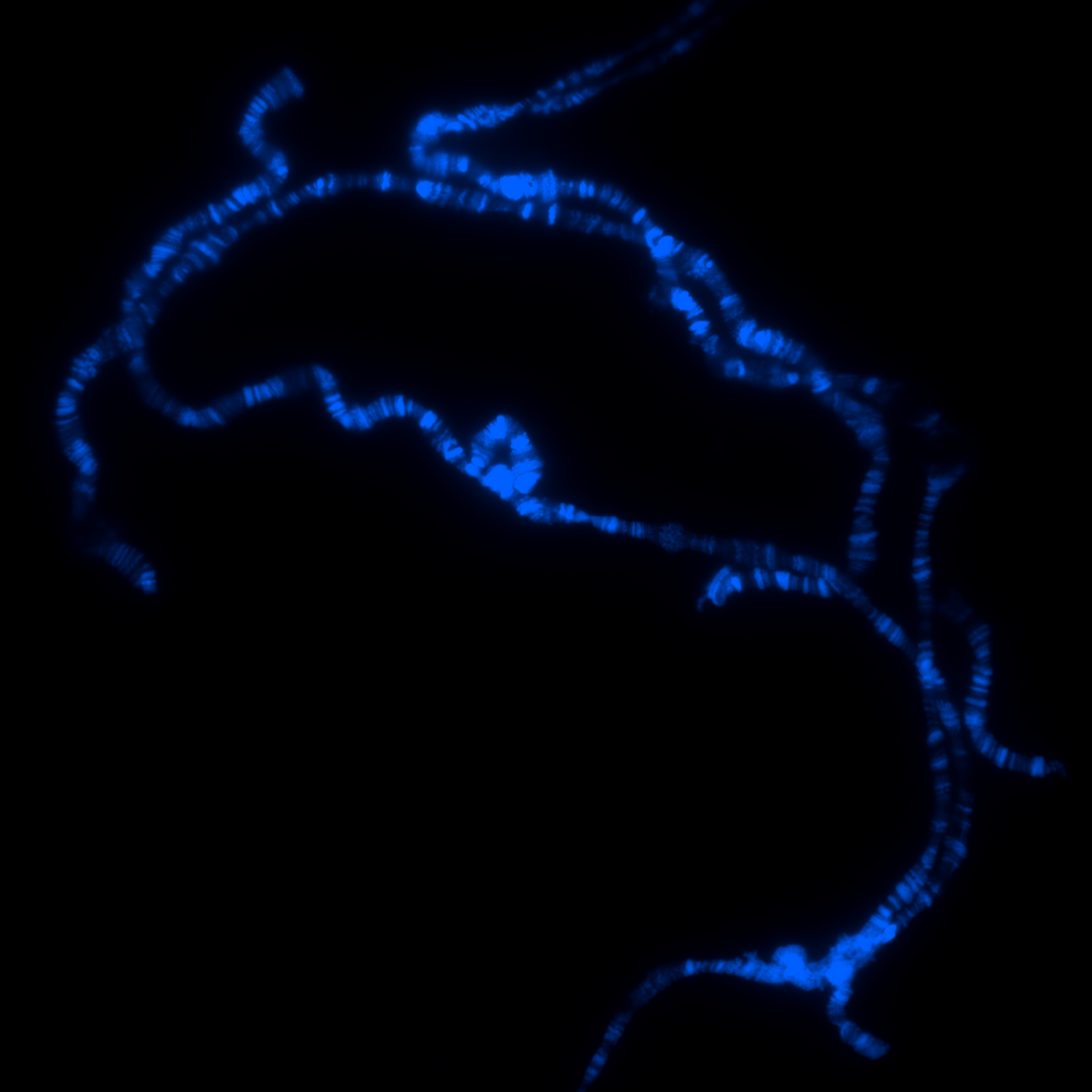

Supplement: Figure 2—source data 1. [file elife-93241-fig2-data1.zip › WT/20200310-msl1wt-11-4-D.tif (RGB).tif]

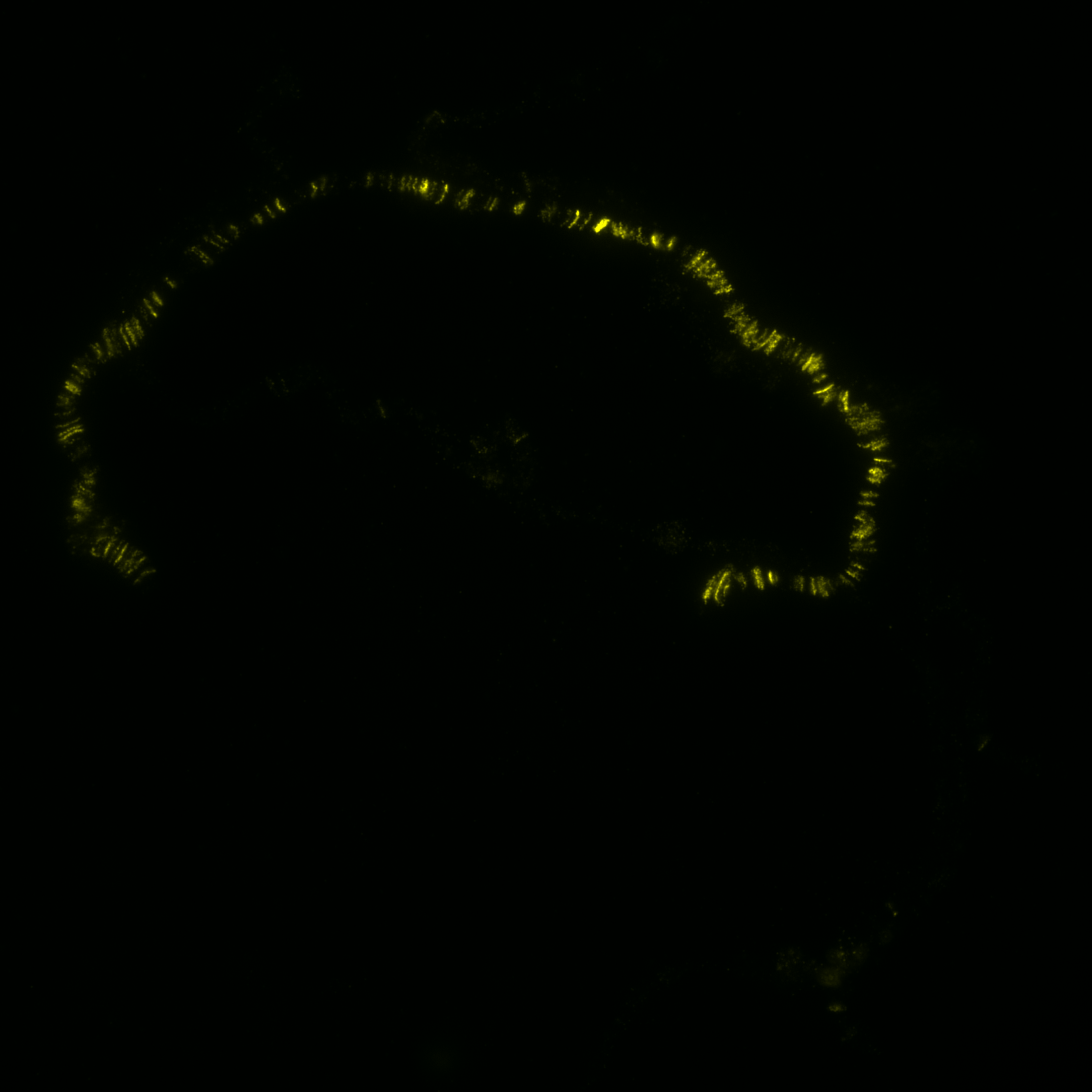

Supplement: Figure 2—source data 1. [file elife-93241-fig2-data1.zip › WT/20200310-msl1wt-11-4-F.tif (RGB).tif]

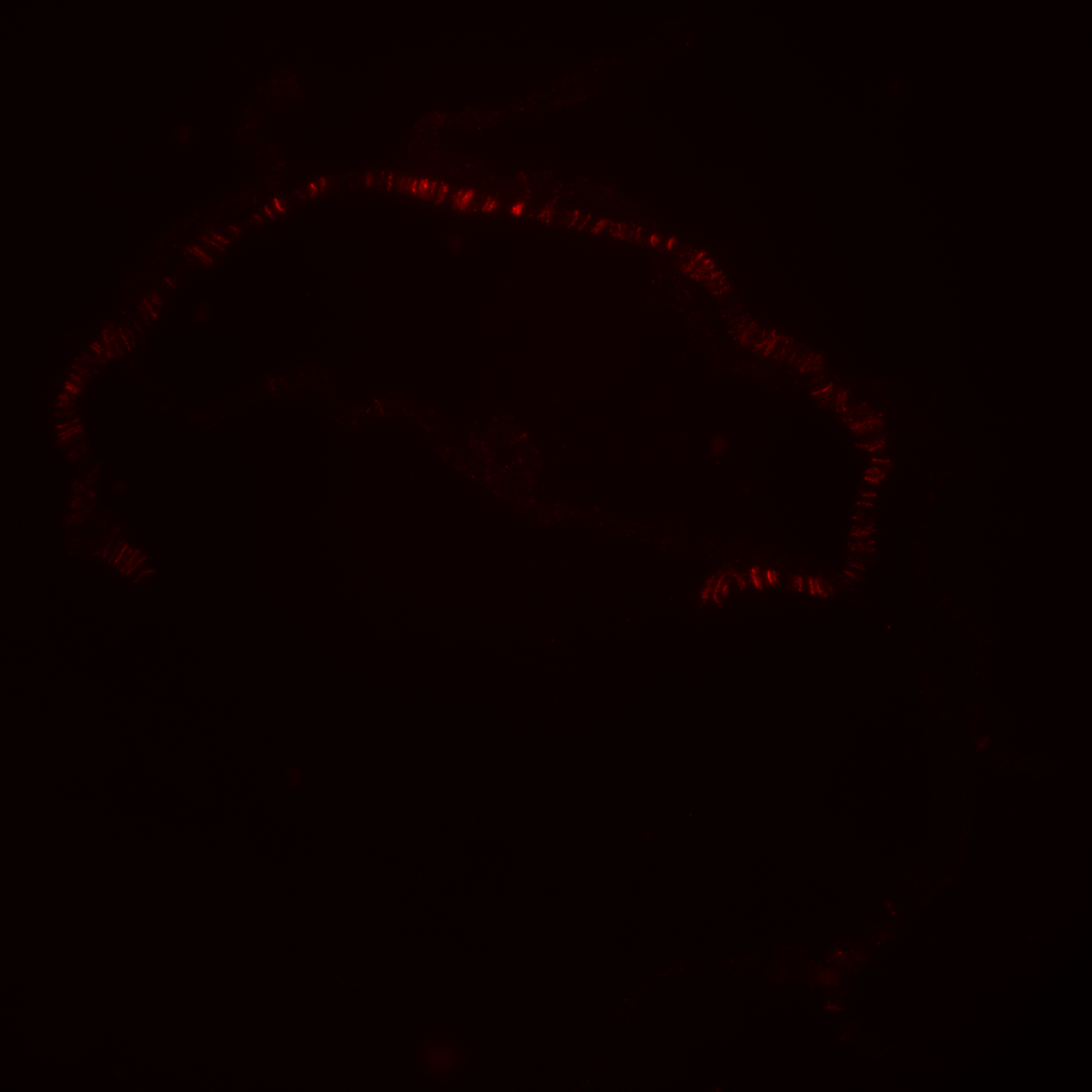

Supplement: Figure 2—source data 1. [file elife-93241-fig2-data1.zip › WT/20200310-msl1wt-11-4-T.tif (RGB).tif]

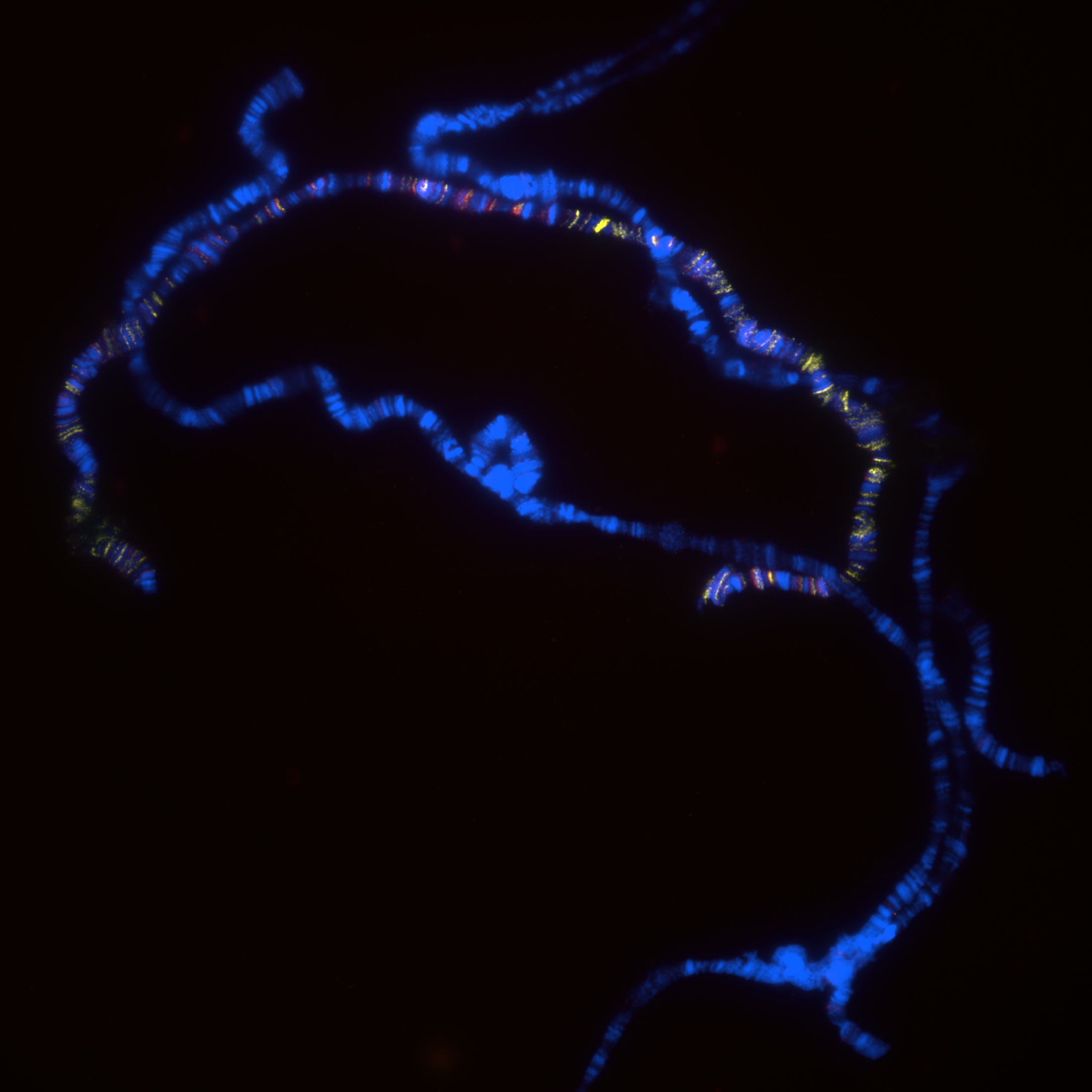

Supplement: Figure 2—source data 1. [file elife-93241-fig2-data1.zip › WT/Composite DFT.tif]

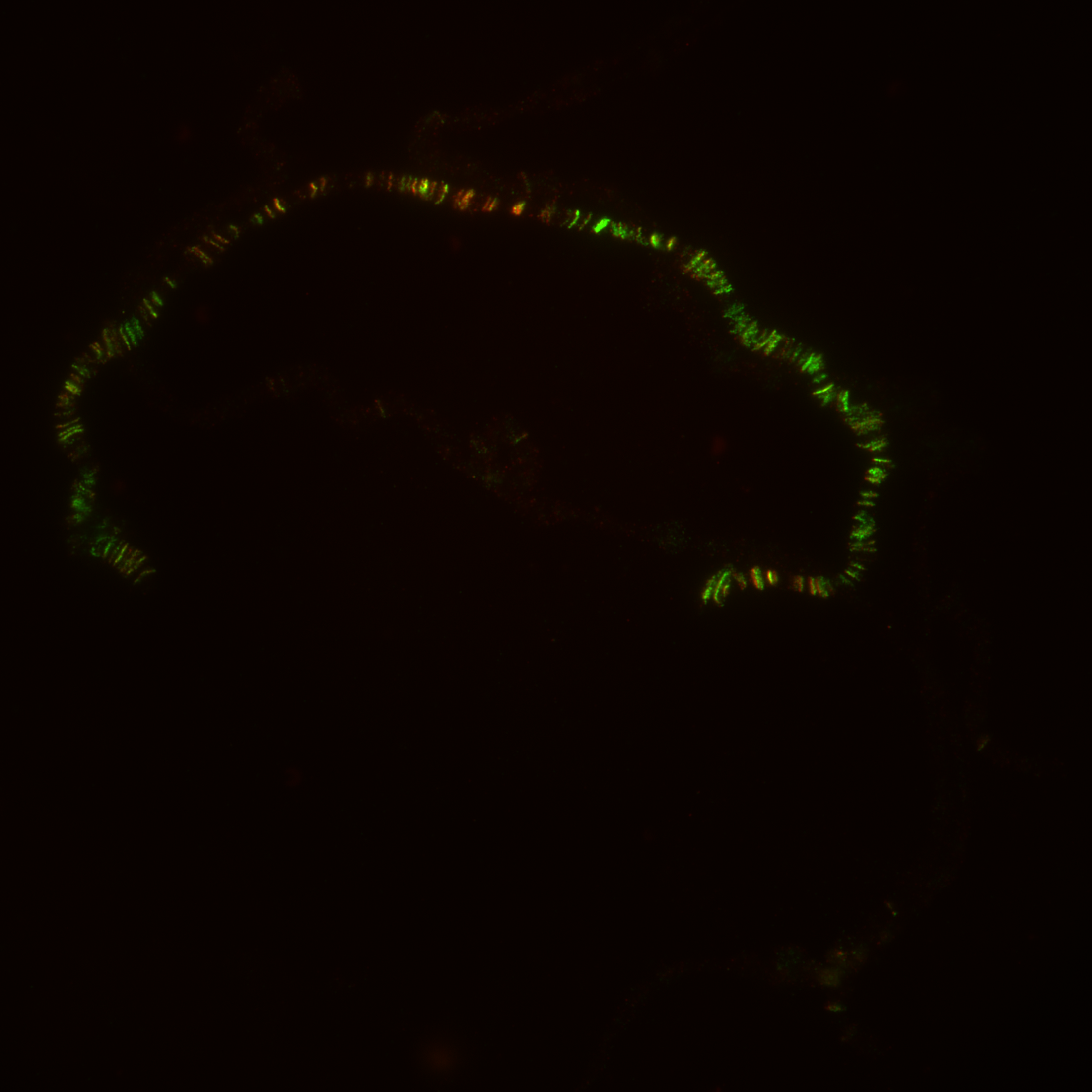

Supplement: Figure 2—source data 1. [file elife-93241-fig2-data1.zip › WT/Composite FT.tif]

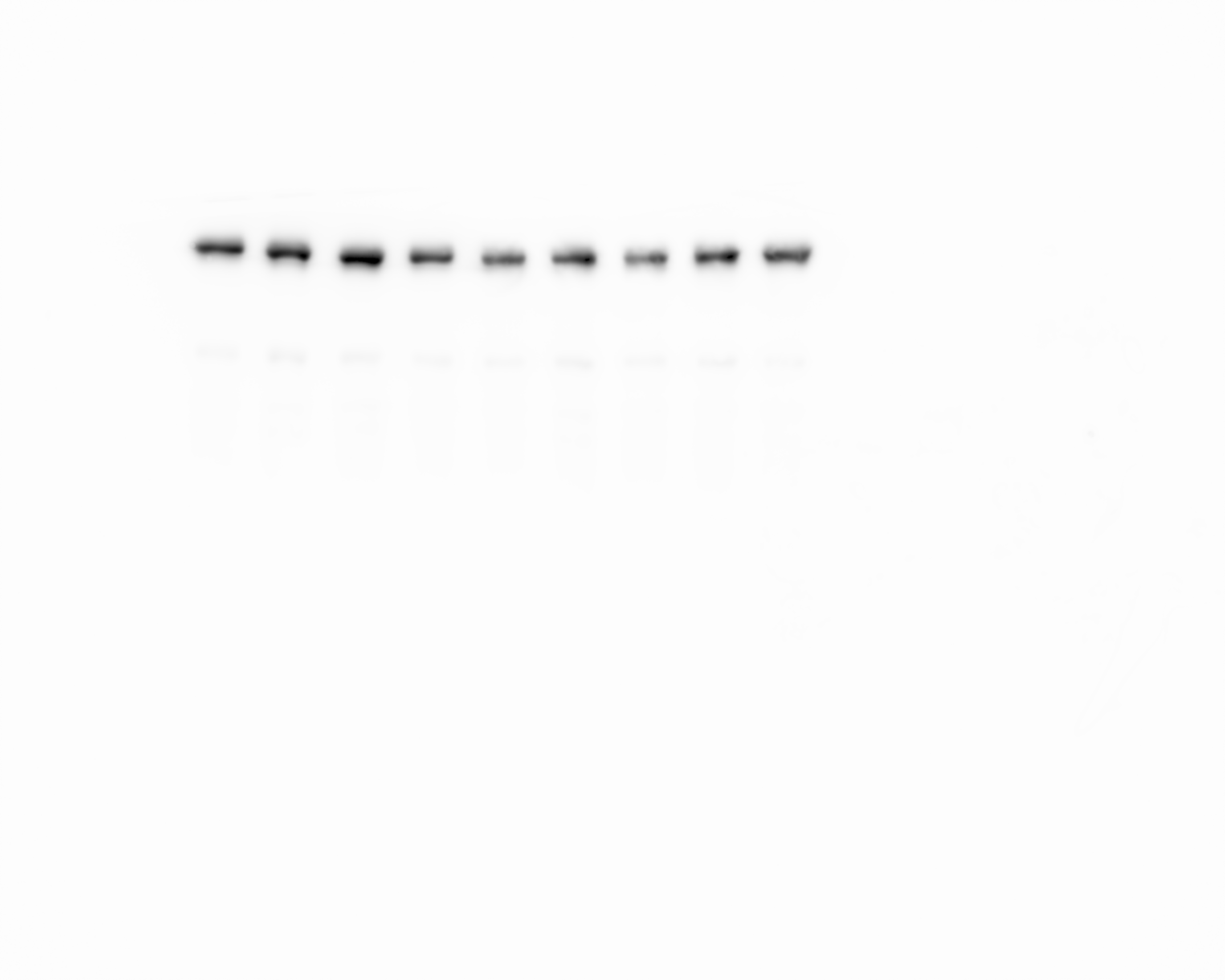

Supplement: Figure 3—source data 1. [file elife-93241-fig3-data1.zip › B_lamin_m1_wtgs2a3s_4.tif]

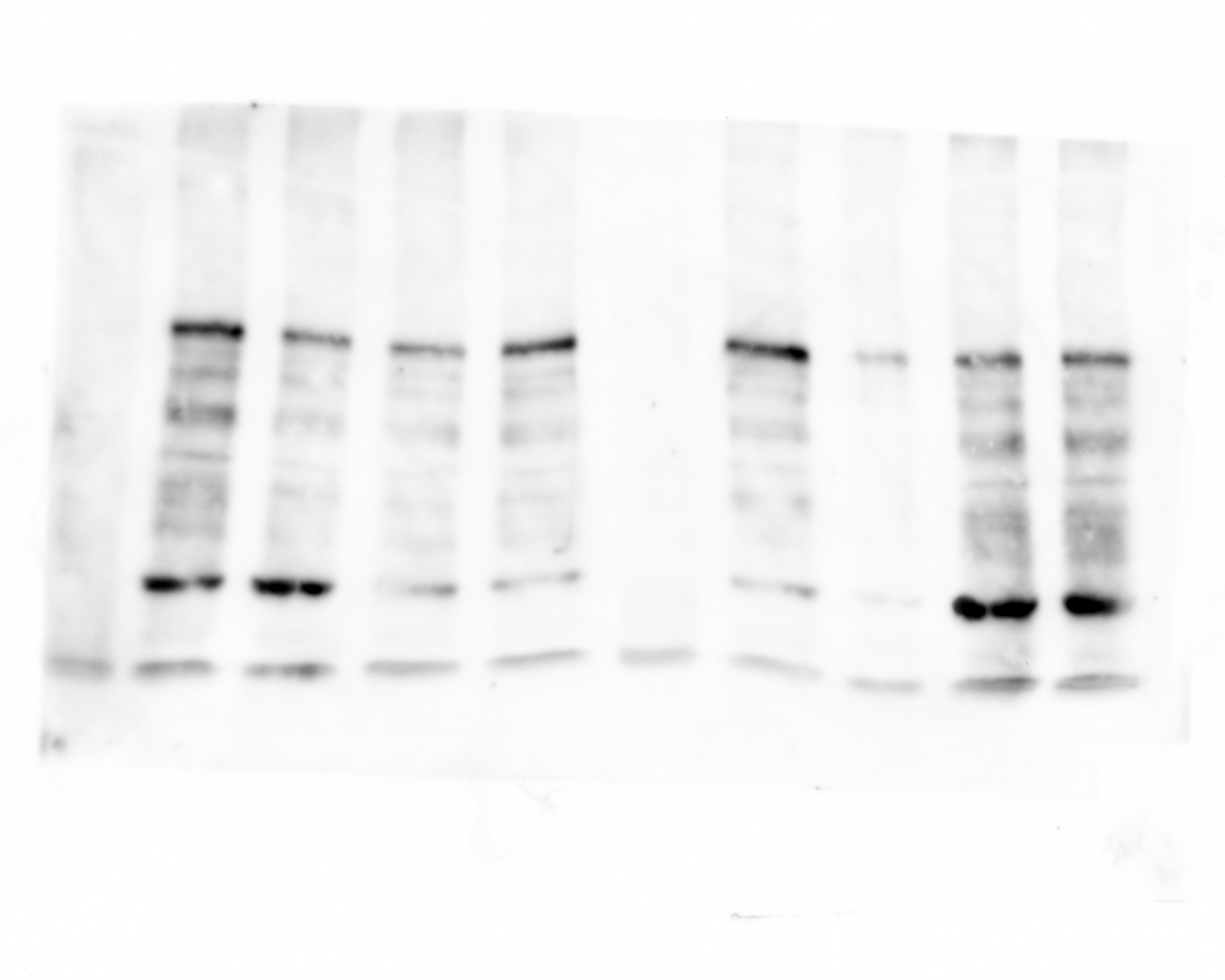

Supplement: Figure 3—source data 1. [file elife-93241-fig3-data1.zip › BC_msl1_wt2a3sgs.tif]

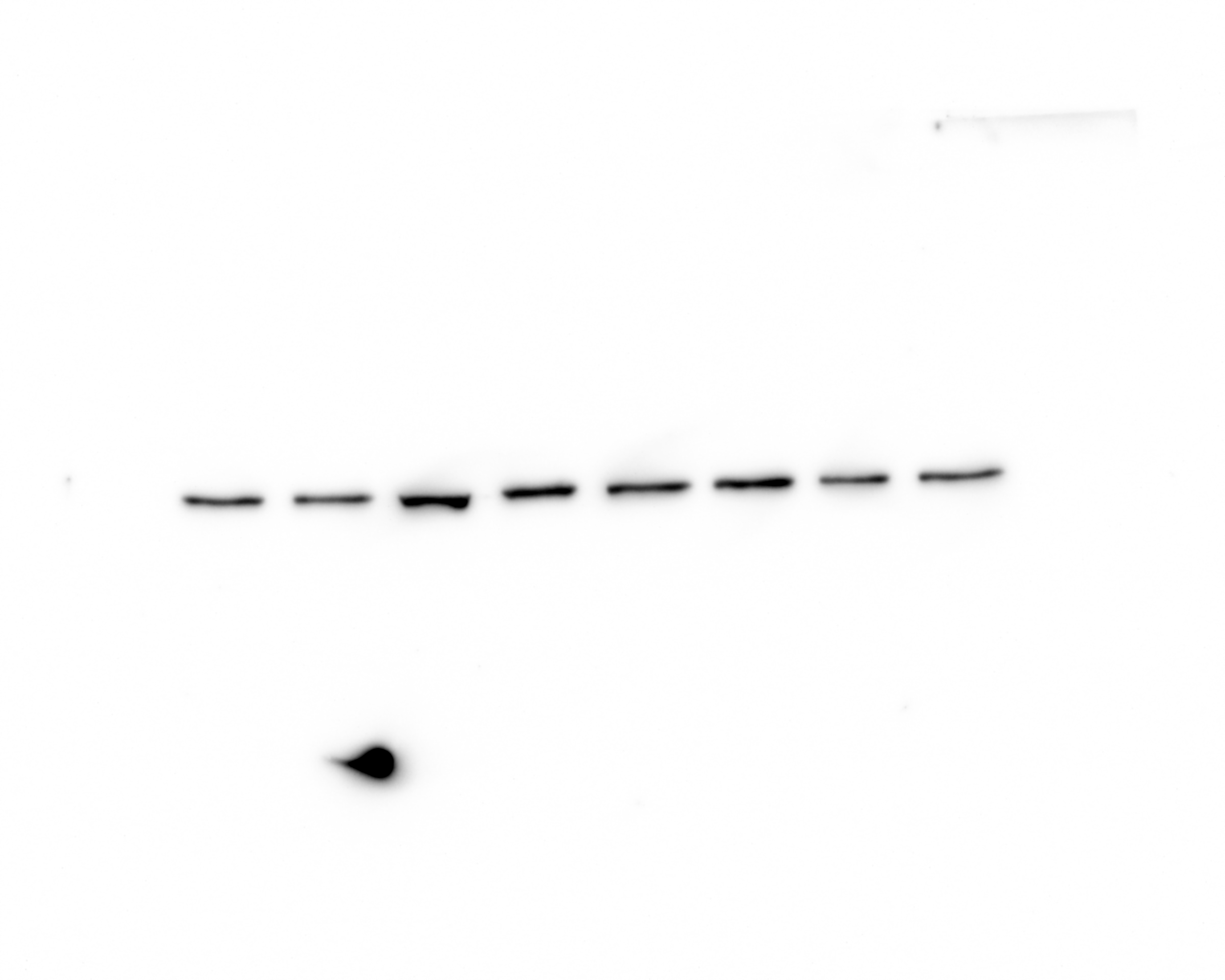

Supplement: Figure 3—source data 1. [file elife-93241-fig3-data1.zip › C_lamin_m1m2_wtgs2a3s_4(Composite).tif]

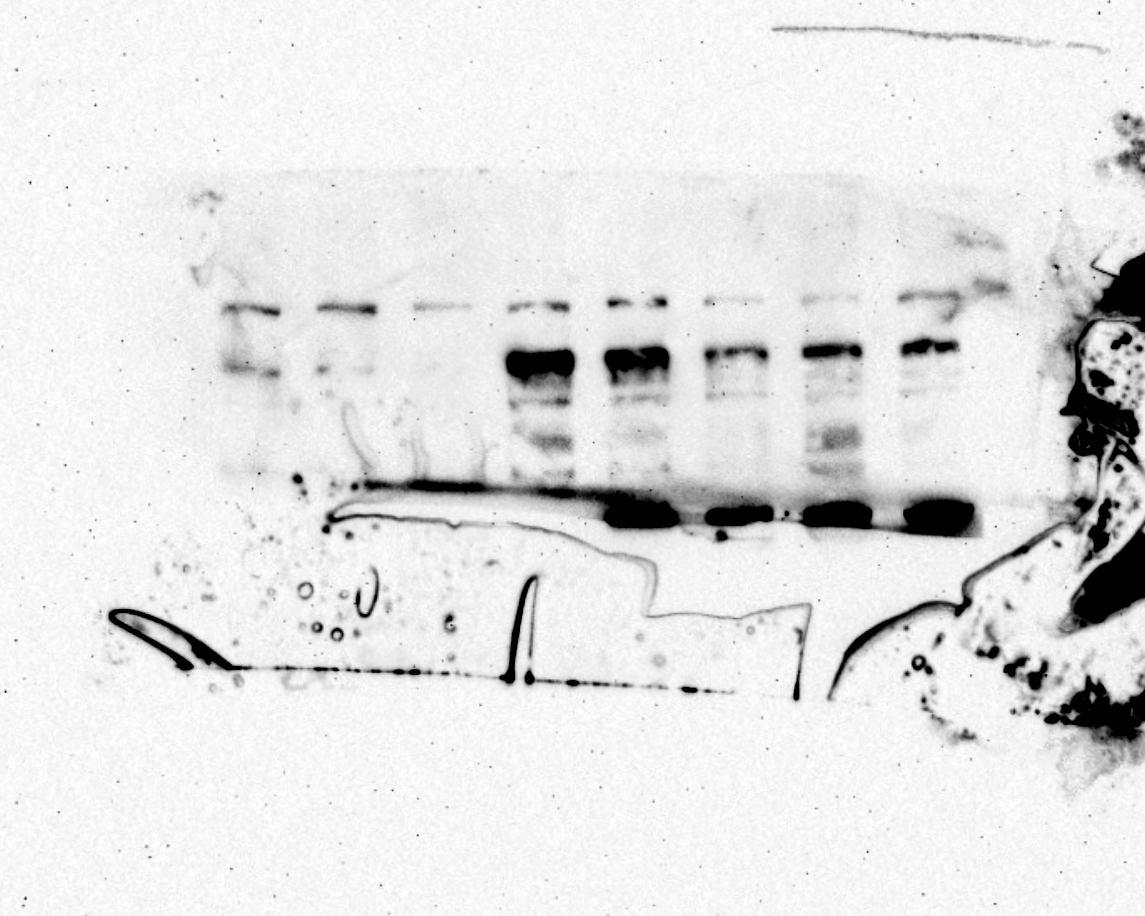

Supplement: Figure 3—source data 1. [file elife-93241-fig3-data1.zip › C_msl2_m1m2_wt2a3sgs.tif]

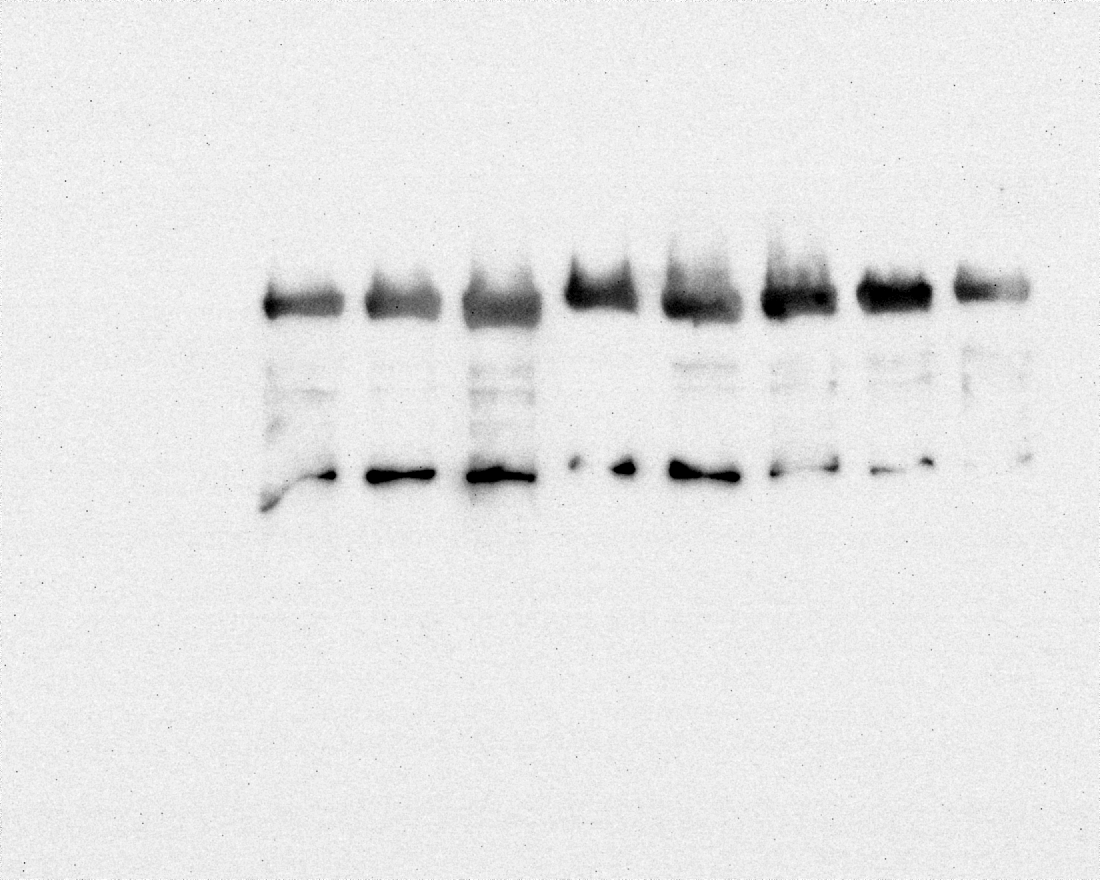

Supplement: Figure 3—source data 1. [file elife-93241-fig3-data1.zip › C_msl3_m1m2_wt2a3sgs.tif]

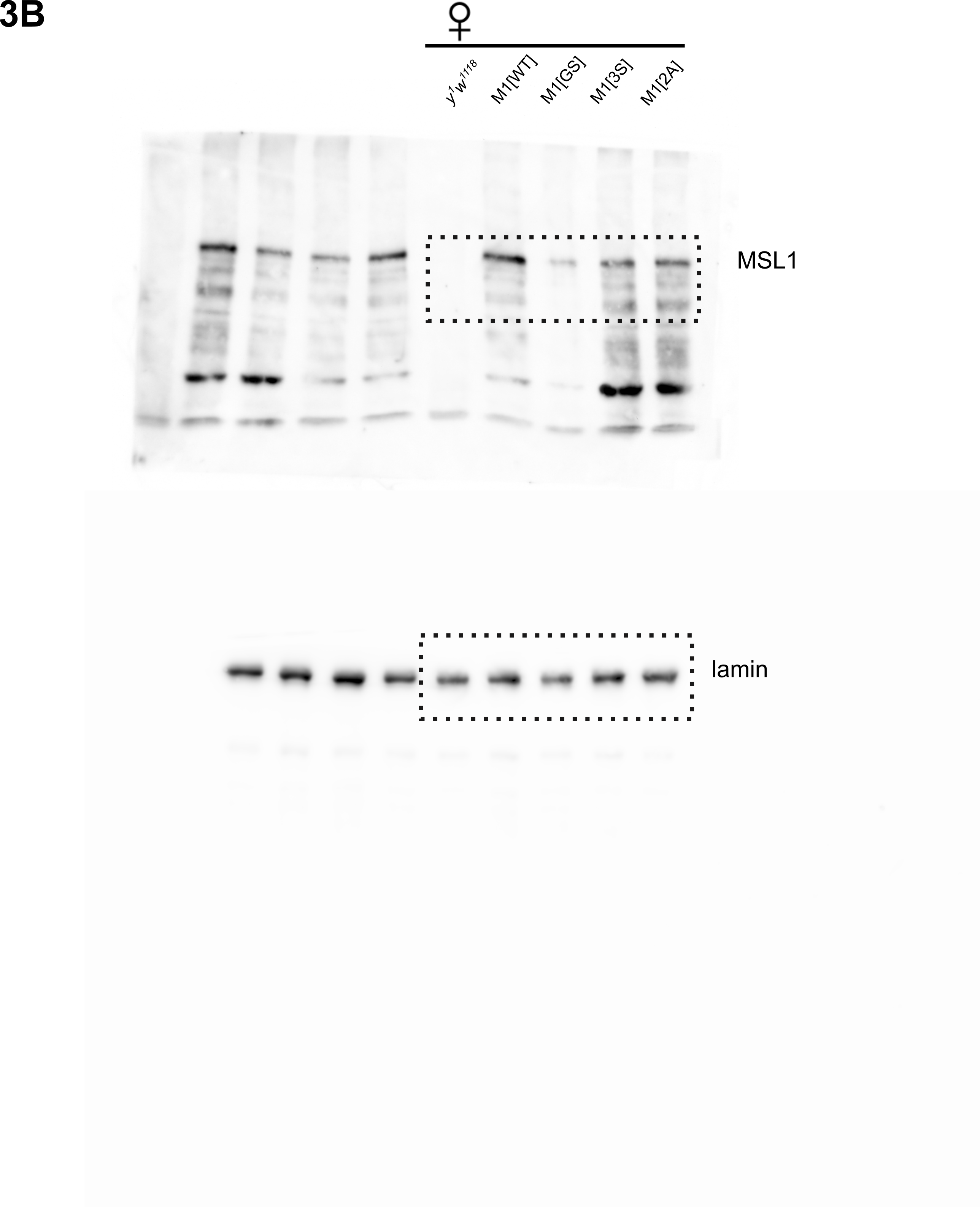

Supplement: Figure 3—source data 2. [file elife-93241-fig3-data2.zip › 3B.png]

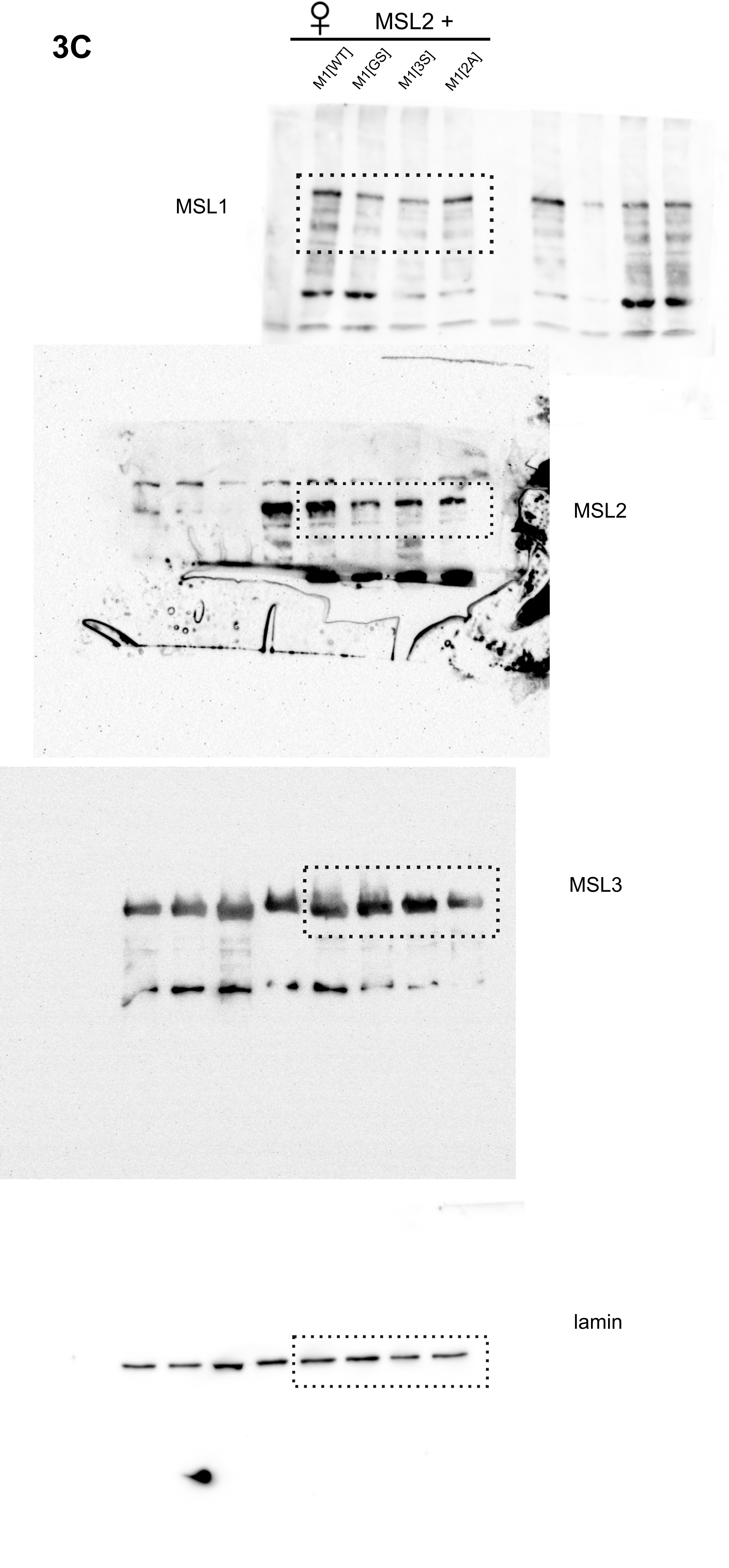

Supplement: Figure 3—source data 2. [file elife-93241-fig3-data2.zip › 3C.png]

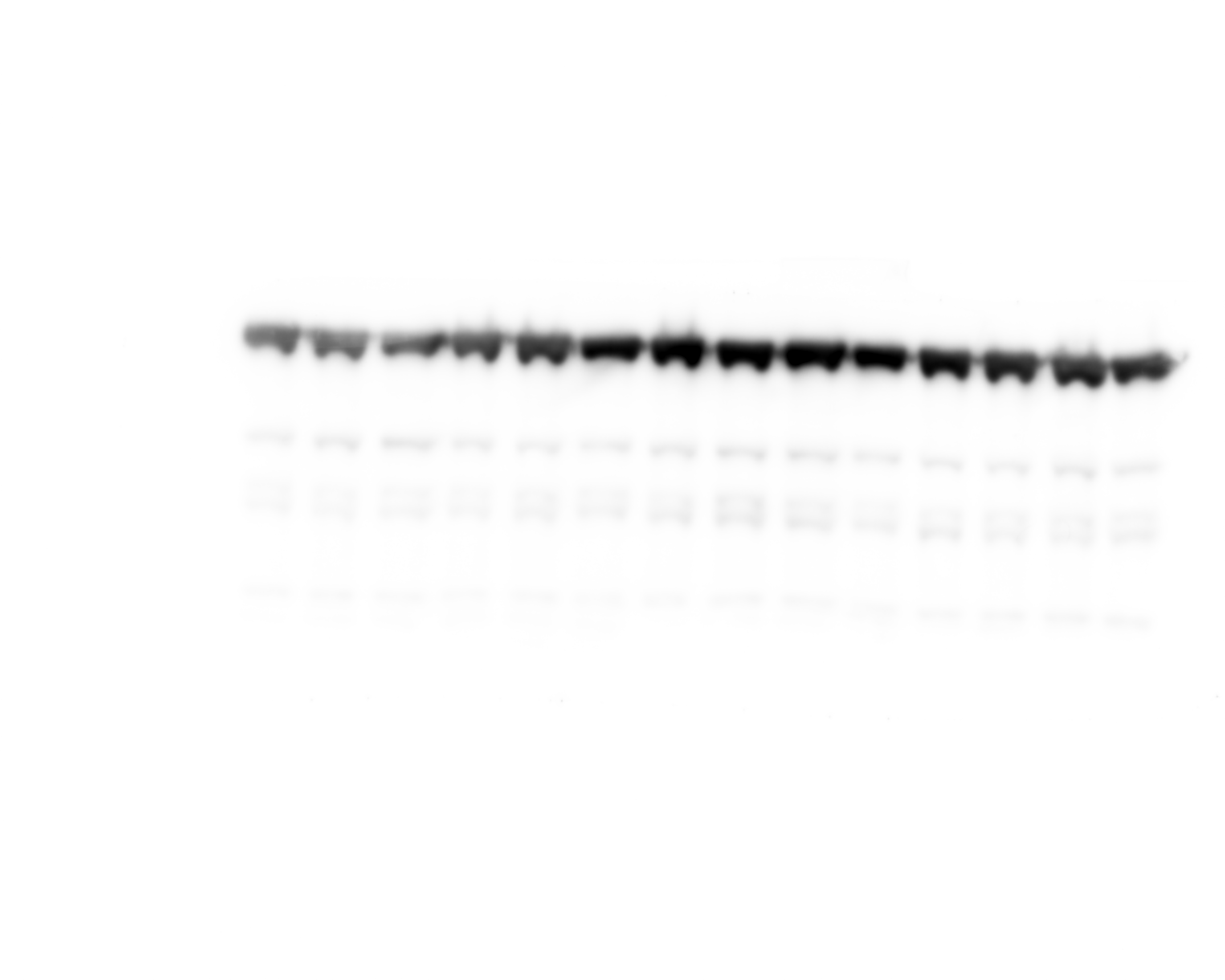

Supplement: Figure 4—source data 1. [file elife-93241-fig4-data1.zip › A_ lamin.tif]

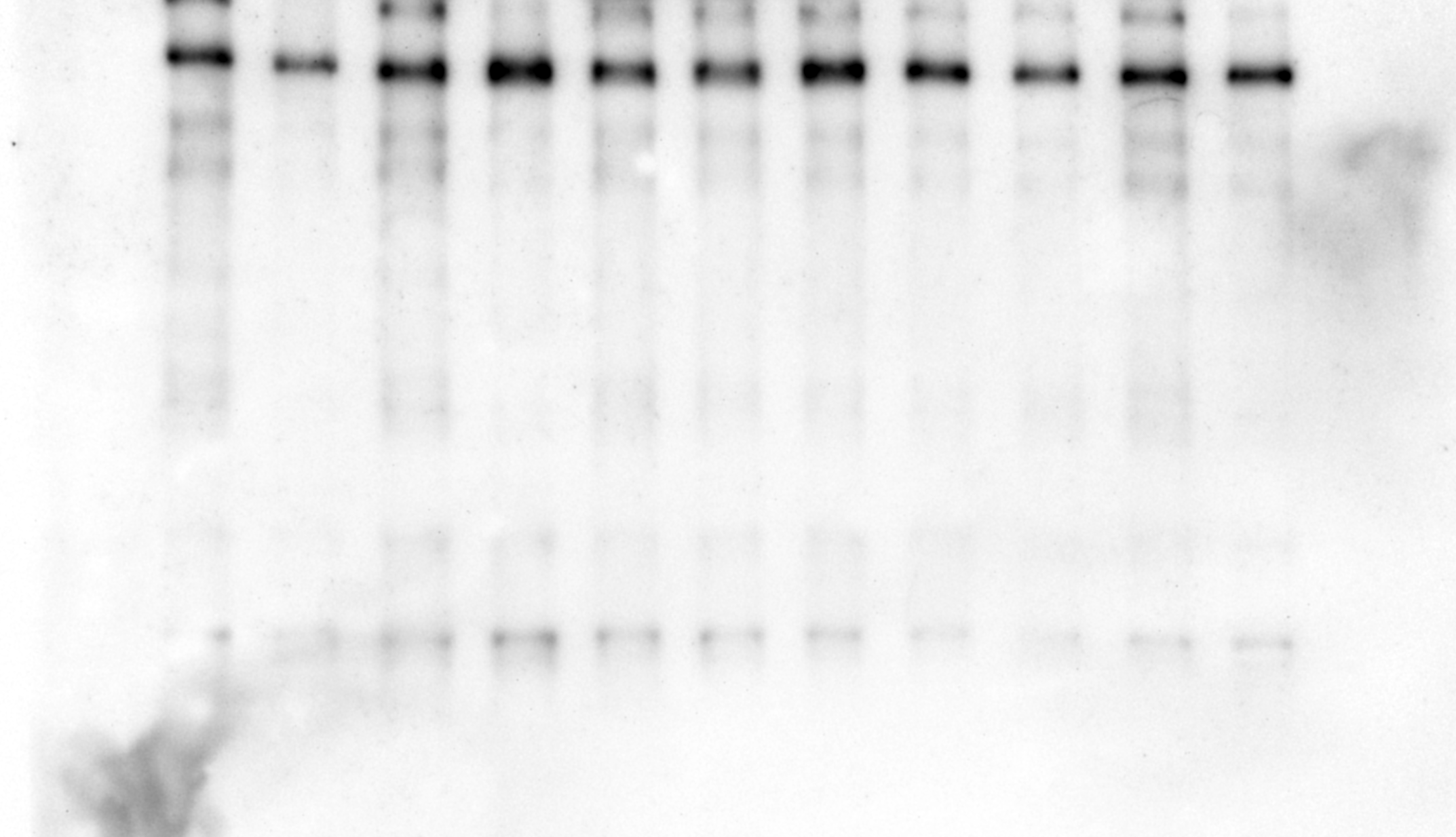

Supplement: Figure 4—source data 1. [file elife-93241-fig4-data1.zip › A_FLAG msl2 S2.tif]

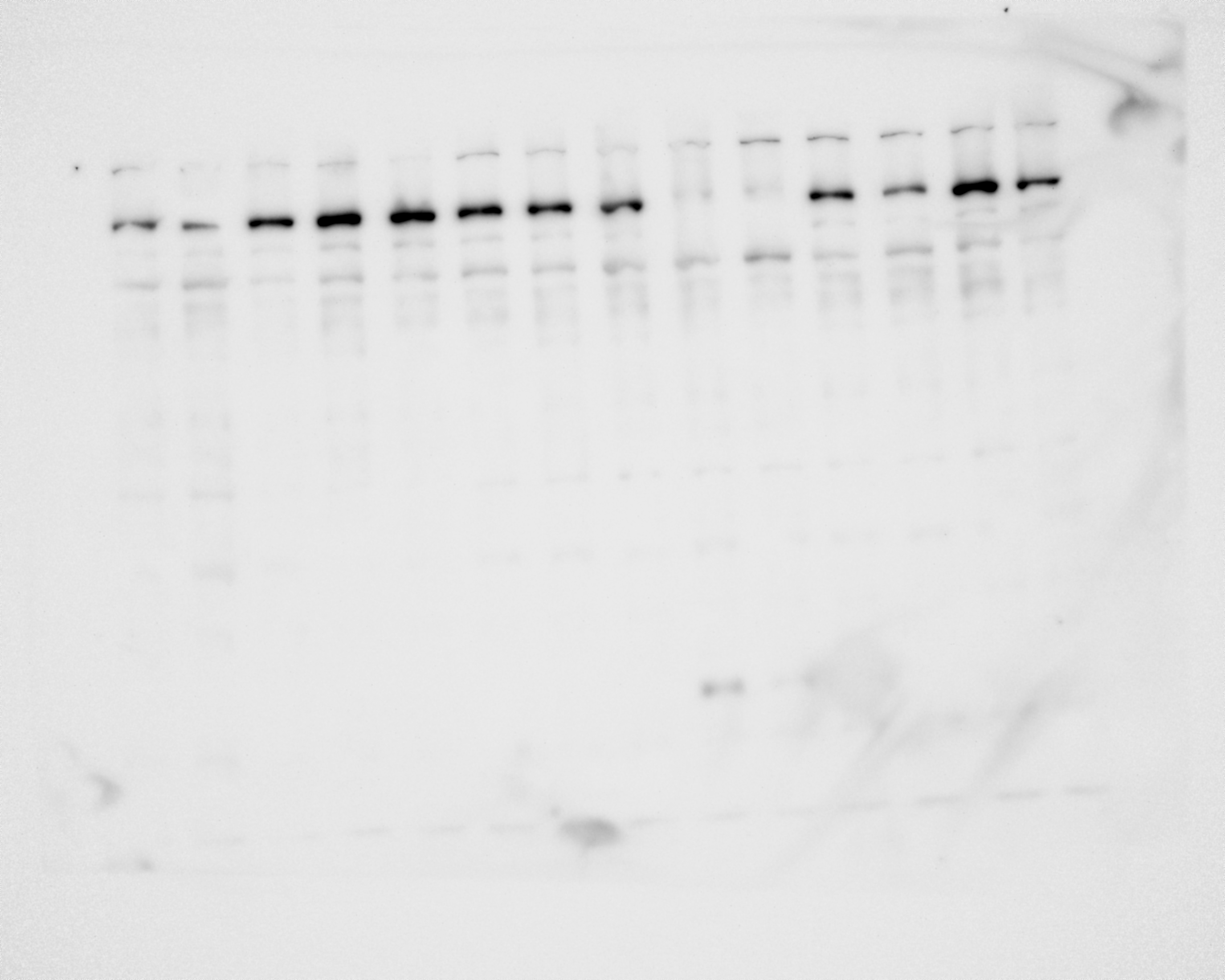

Supplement: Figure 4—source data 1. [file elife-93241-fig4-data1.zip › A_HA msl1 S2.tif]

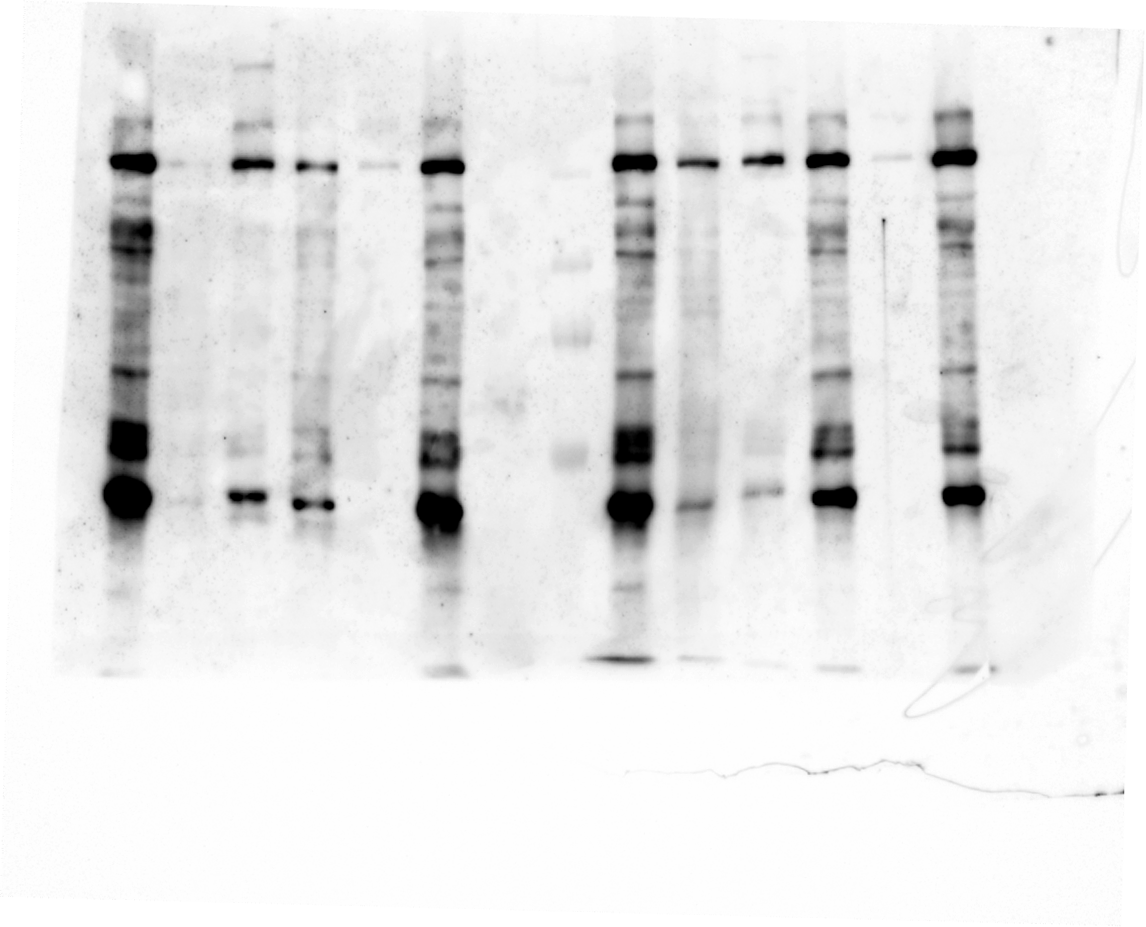

Supplement: Figure 4—source data 1. [file elife-93241-fig4-data1.zip › B_flag ip 2mut gs(Chemiluminescence).tif]

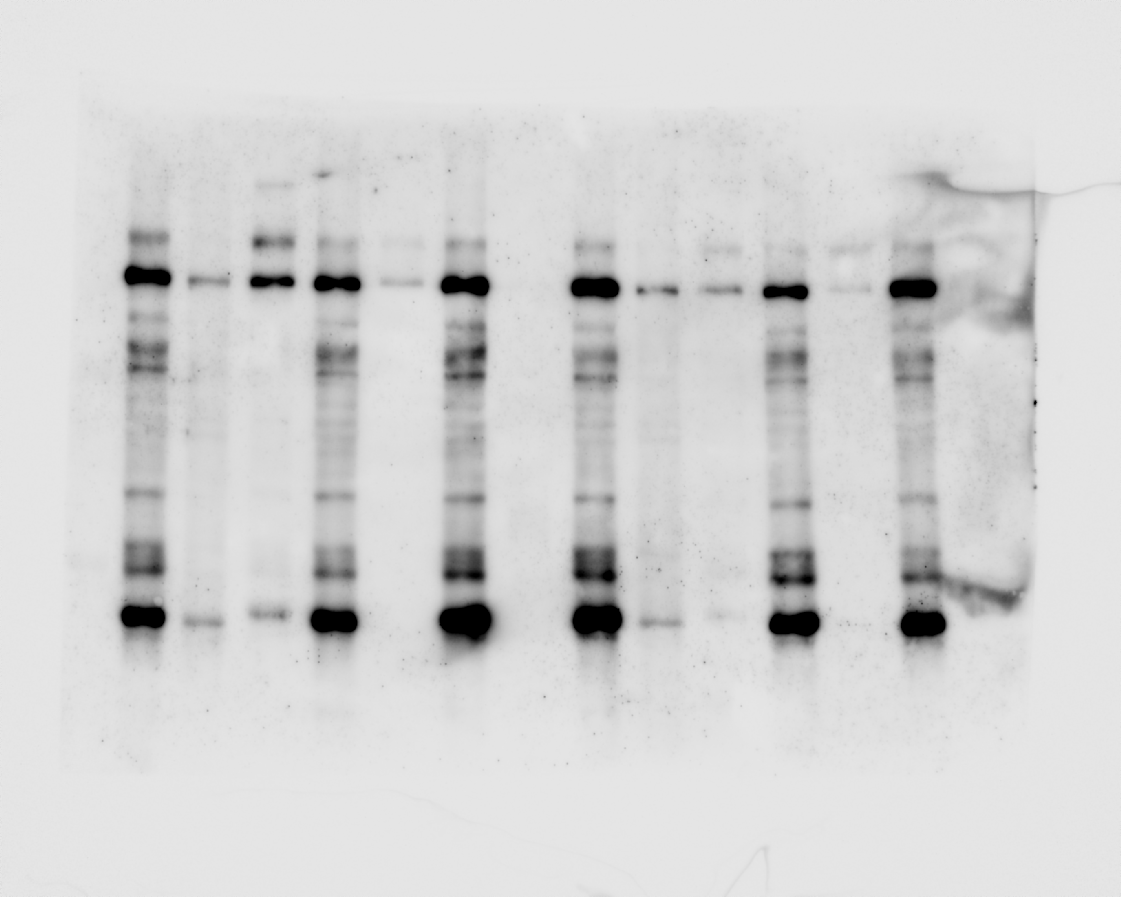

Supplement: Figure 4—source data 1. [file elife-93241-fig4-data1.zip › B_flag ip 24-39 8-20(Chemiluminescence).tif]

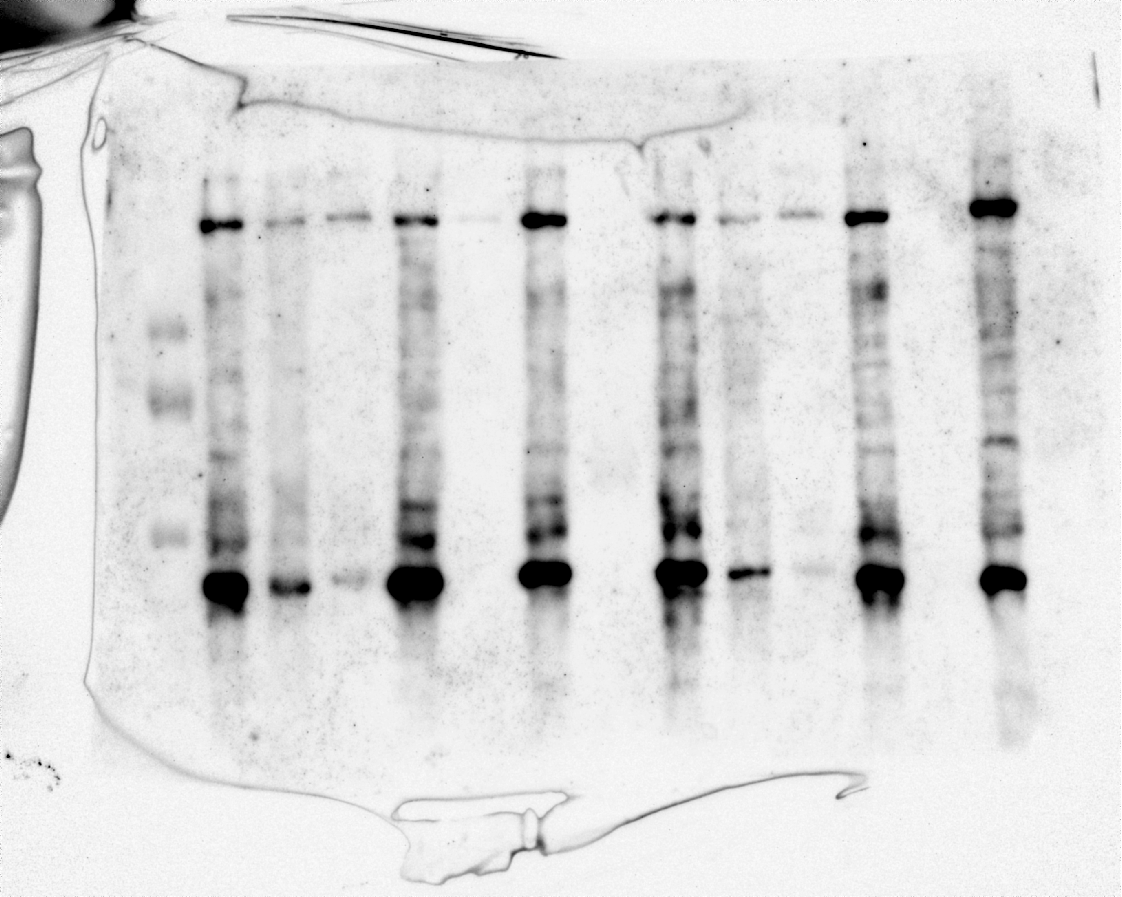

Supplement: Figure 4—source data 1. [file elife-93241-fig4-data1.zip › B_flag ip 3mut 3mutA 2(Chemiluminescence).tif]

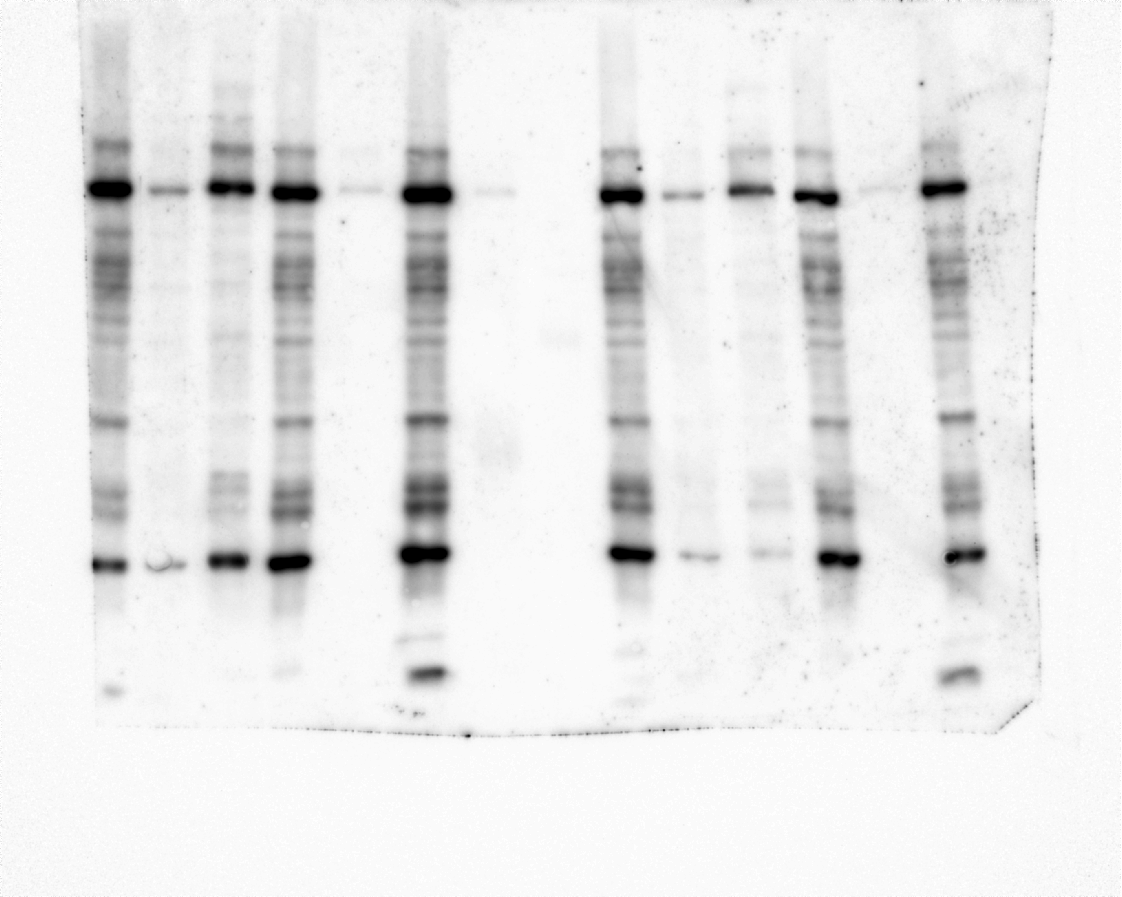

Supplement: Figure 4—source data 1. [file elife-93241-fig4-data1.zip › B_flag ip 3mut gs(Chemiluminescence).tif]

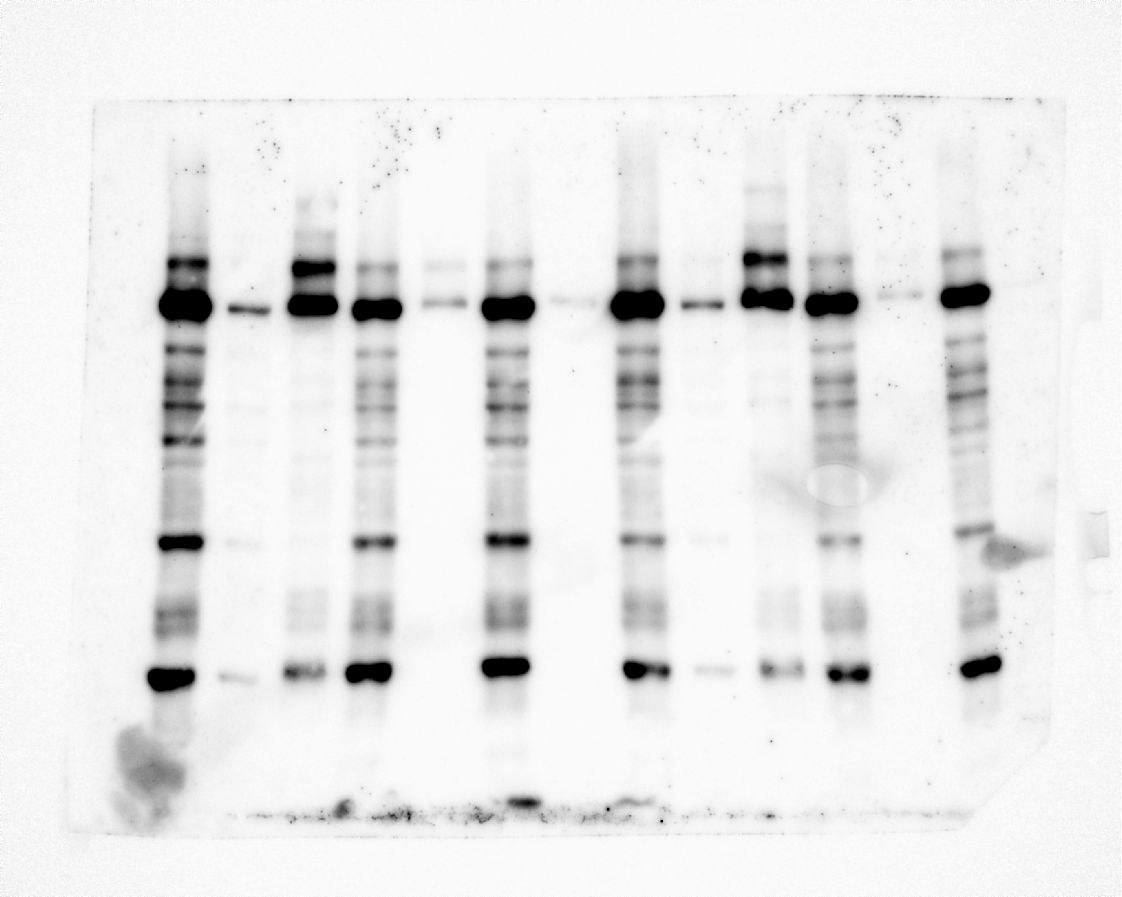

Supplement: Figure 4—source data 1. [file elife-93241-fig4-data1.zip › B_flag ip 41-85(Chemiluminescence).tif]

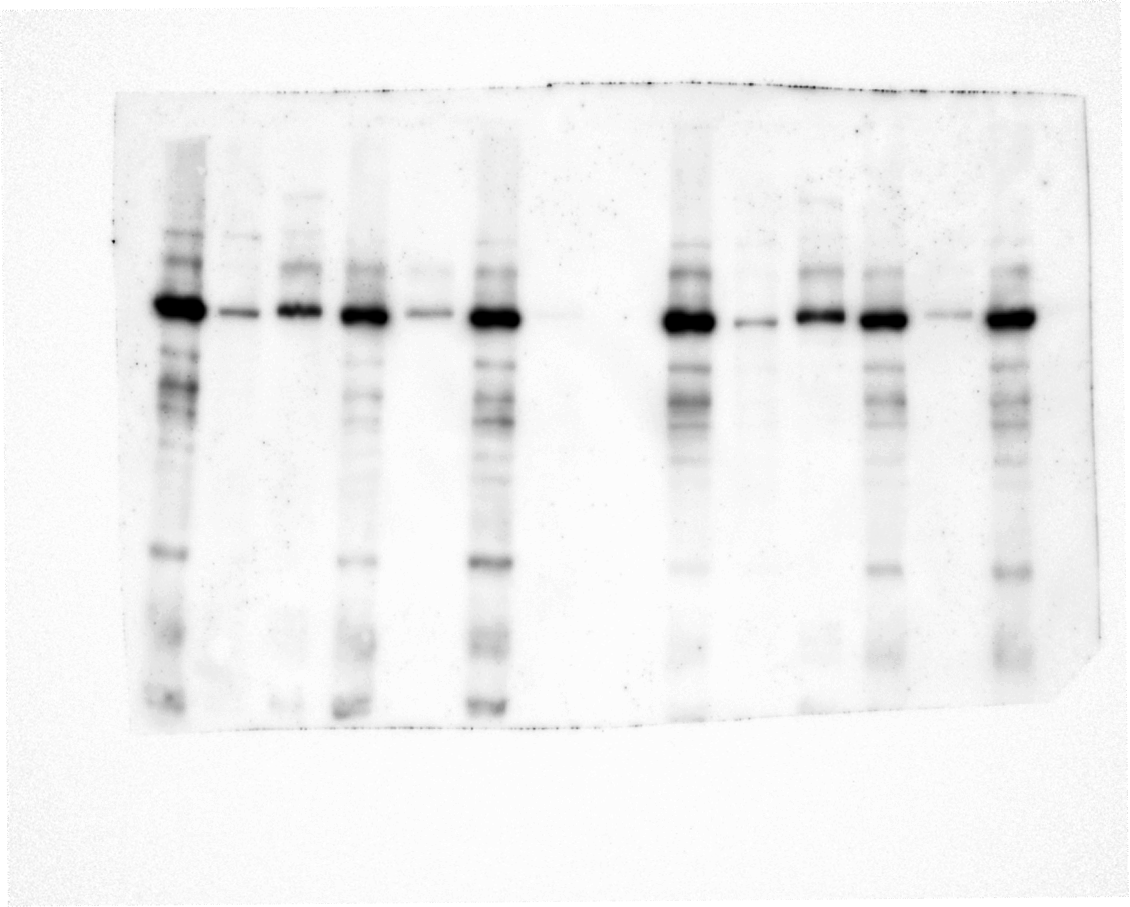

Supplement: Figure 4—source data 1. [file elife-93241-fig4-data1.zip › B_flag ip 8-20 24-39(Chemiluminescence).tif]

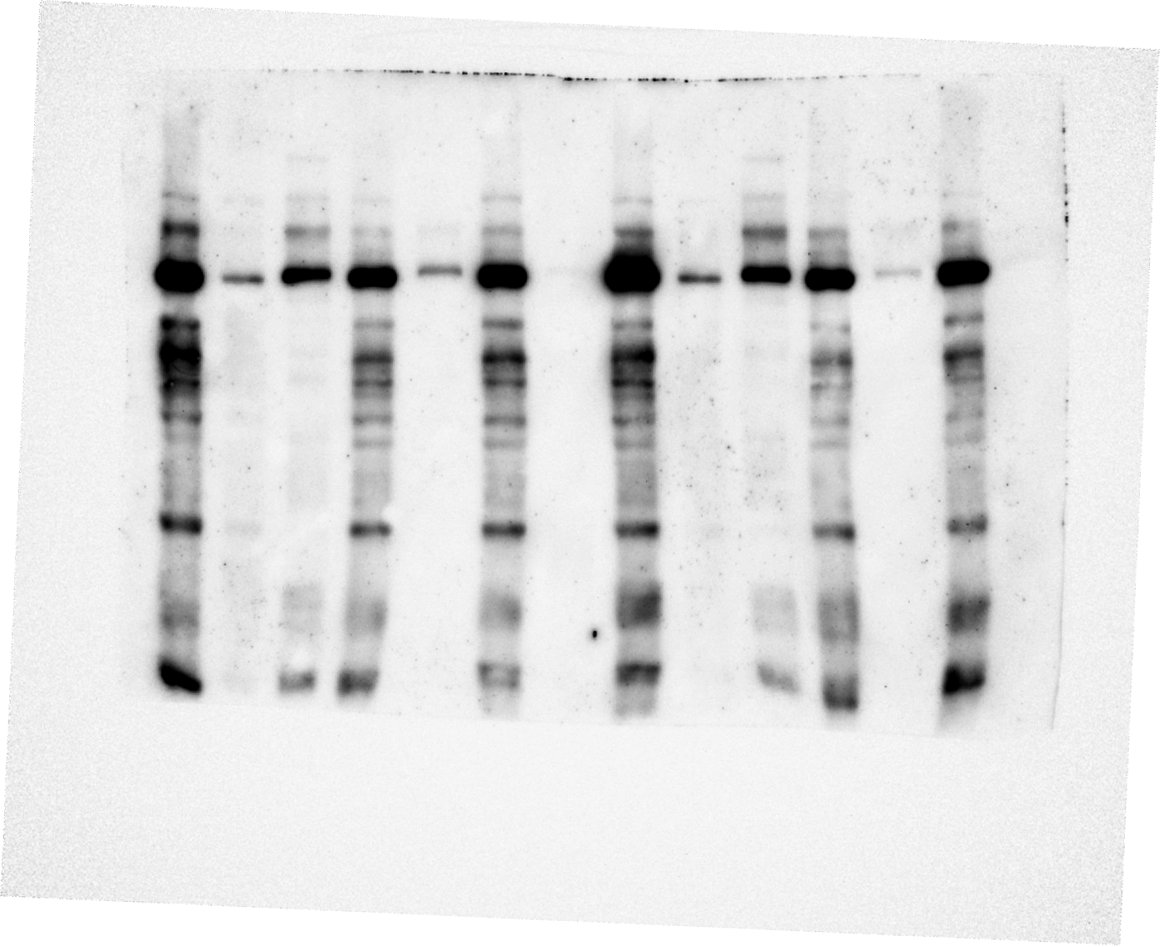

Supplement: Figure 4—source data 1. [file elife-93241-fig4-data1.zip › B_flag ip wt 3mut_2(Chemiluminescence).tif]

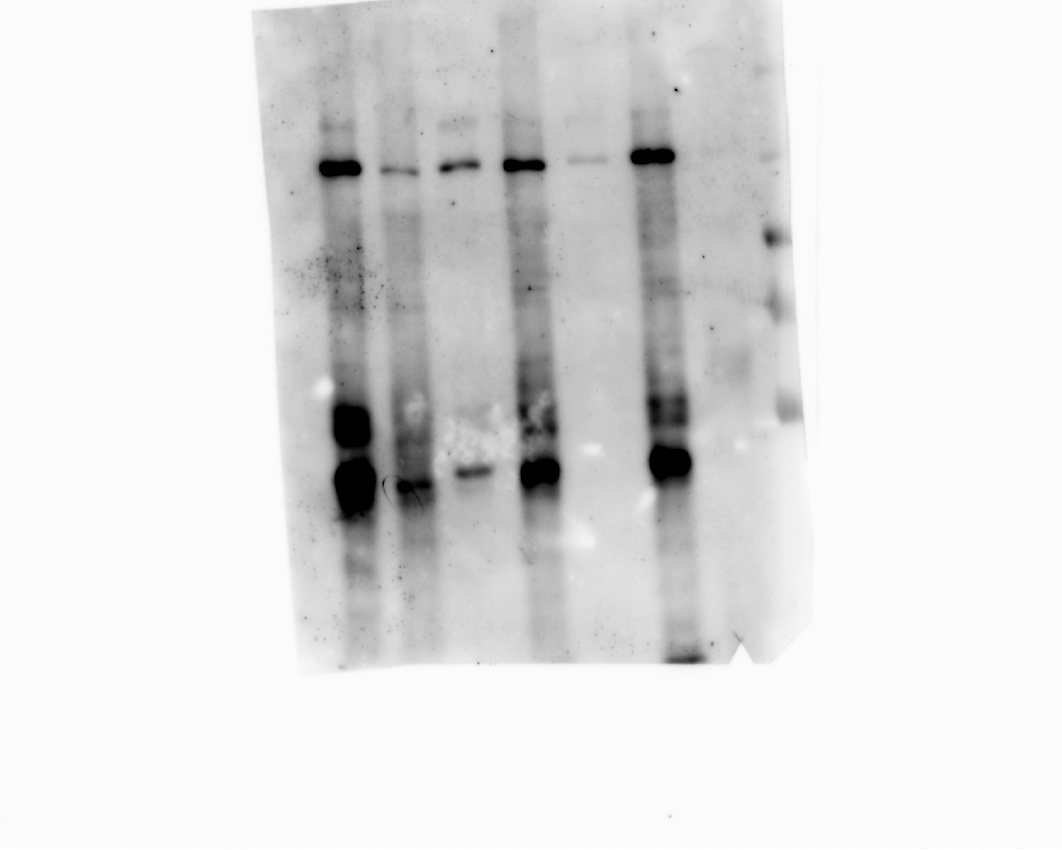

Supplement: Figure 4—source data 1. [file elife-93241-fig4-data1.zip › B_flag ip wt(Chemiluminescence).tif]

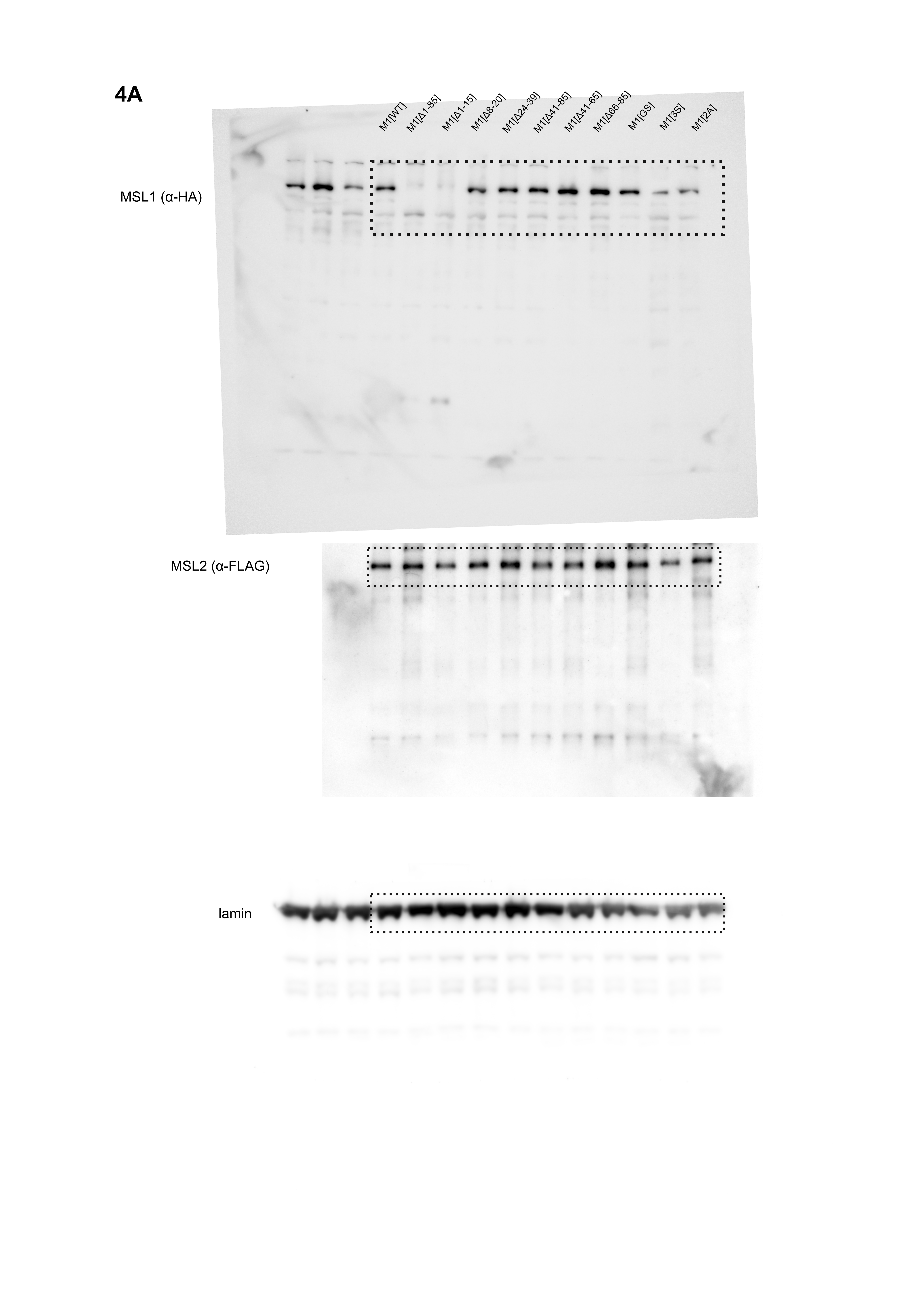

Supplement: Figure 4—source data 2. [file elife-93241-fig4-data2.zip › 4A.png]

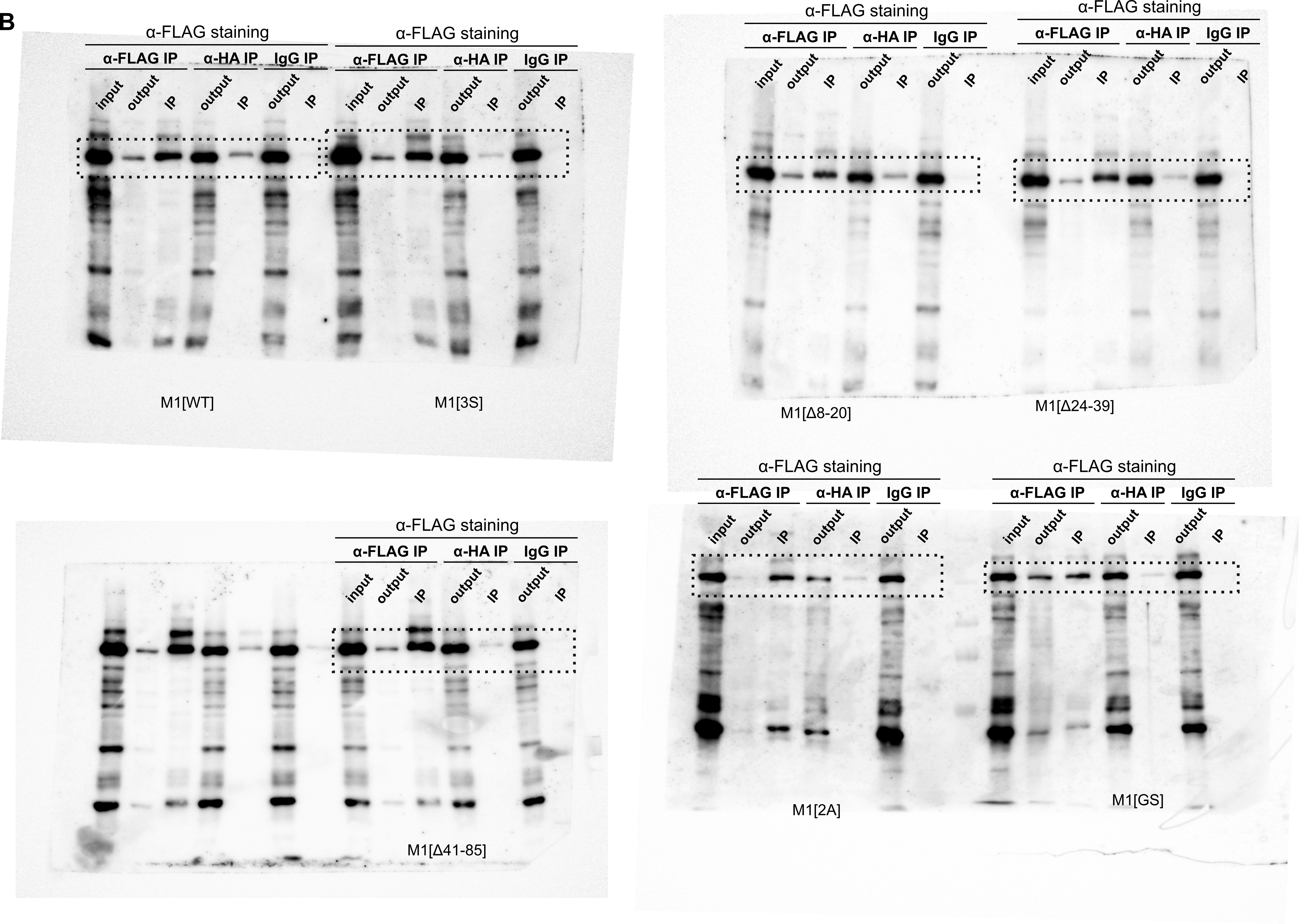

Supplement: Figure 4—source data 2. [file elife-93241-fig4-data2.zip › 4B.png]

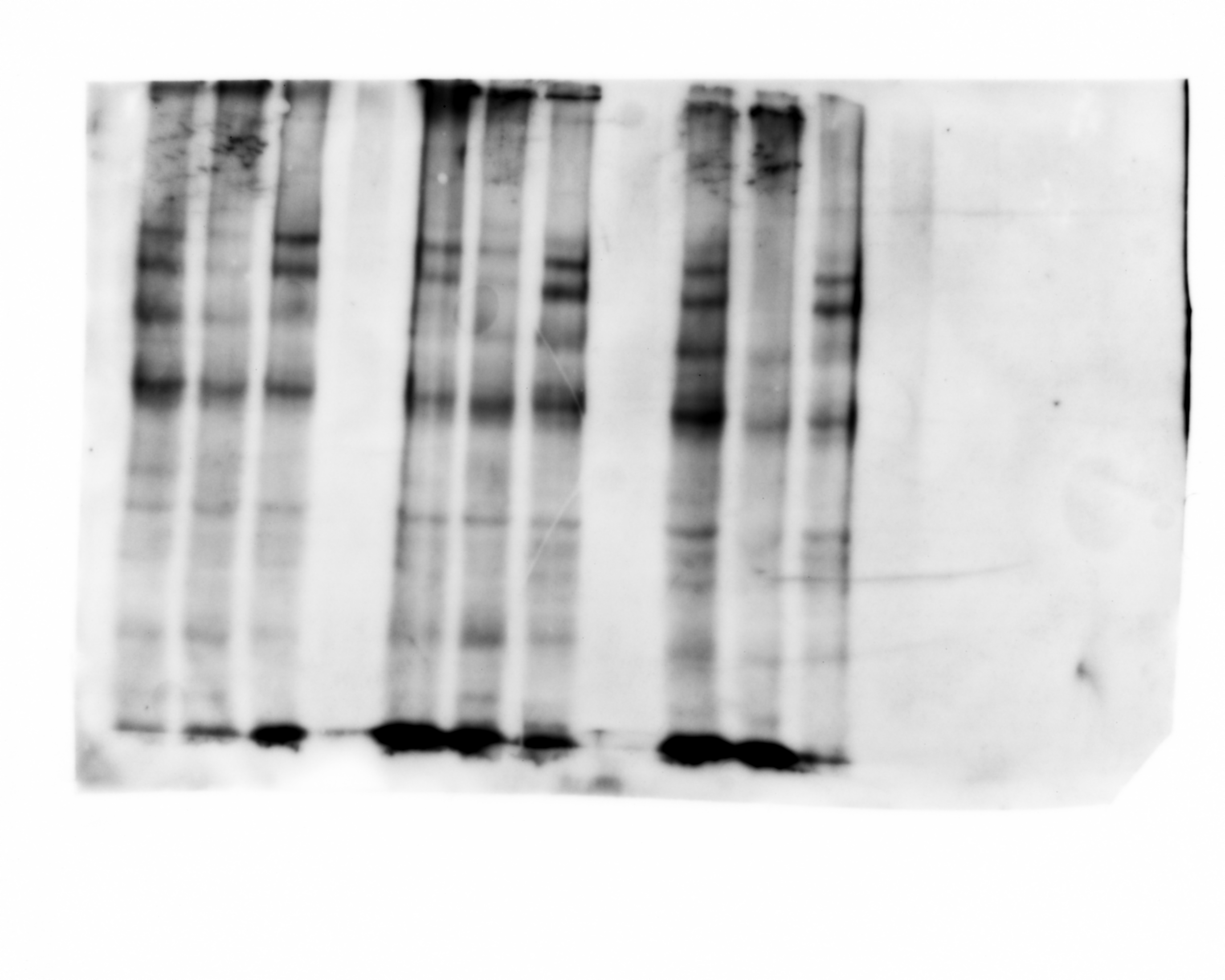

Supplement: Figure 7—source data 1. [file elife-93241-fig7-data1.zip › B_ip msl1_ha_6(Chemiluminescence).raw16.tif]

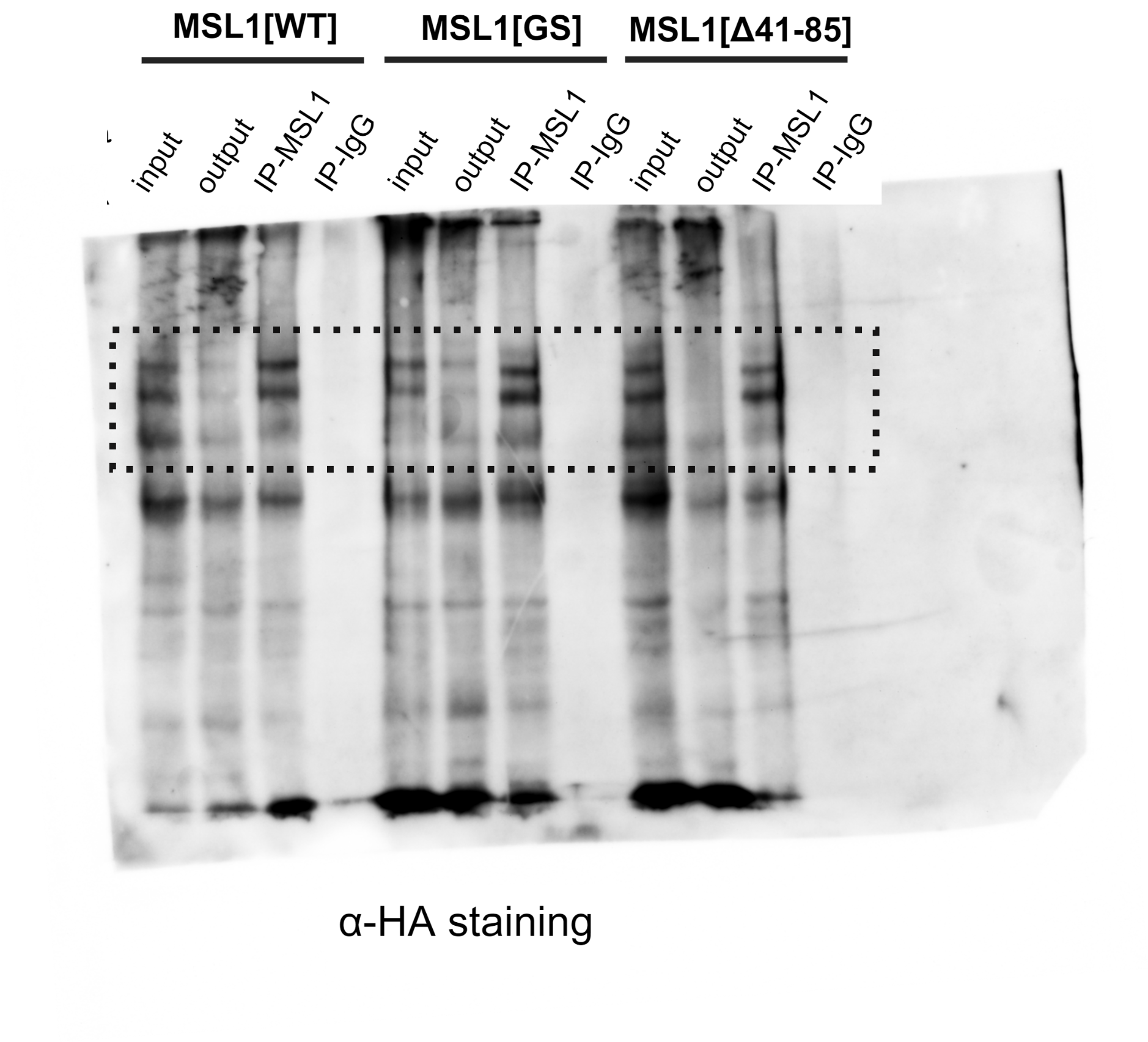

Supplement: Figure 7—source data 2. [file elife-93241-fig7-data2.zip › 5B.png]
